# A One-Pot Synthesis-Functionalization Strategy for Streamlined Access to 2,5-Disubstituted 1,3,4-Oxadiazoles from Carboxylic Acids

**DOI:** 10.1021/acs.joc.2c01669

**Published:** 2022-09-02

**Authors:** Daniel Matheau-Raven, Darren J. Dixon

**Affiliations:** Department of Chemistry, University of Oxford, Chemistry Research Laboratory, 12 Mansfield Road, Oxford OX1 3TA, U.K.

## Abstract

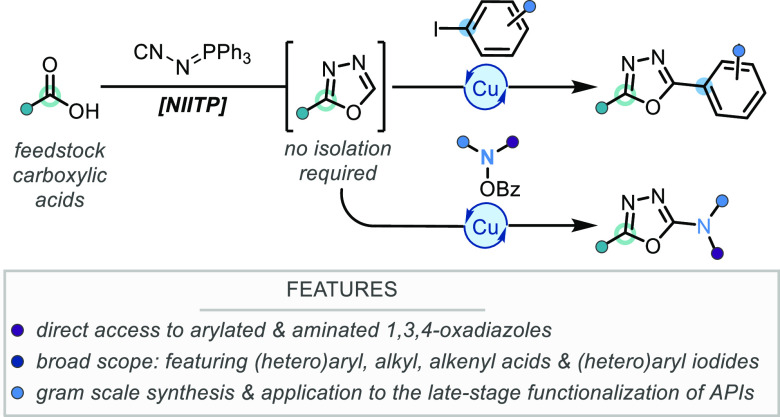

A one-pot 1,3,4-oxadiazole synthesis-arylation strategy for accessing 2,5-disubstituted 1,3,4-oxadiazoles, from carboxylic acids, *N*-isocyaniminotriphenylphosphorane (NIITP), and aryl iodides, is reported. The reaction sequence, featuring a second stage copper-catalyzed 1,3,4-oxadiazole arylation, was found to tolerate (hetero)aryl, alkyl, and alkenyl carboxylic acids, and (hetero)aryl iodide coupling partners. The effectiveness of the two-stage strategy was exemplified by the late-stage functionalization of five carboxylic acid-containing APIs, and an extension to the synthesis of aminated 1,3,4-oxadiazoles using *N*-benzoyloxy amine coupling partners was also demonstrated.

2,5-Disubstituted 1,3,4-oxadiazole motifs are prominent in materials and medicinal chemistry, imparting favorable pharmacokinetic properties and increased hydrolytic stability when applied as ester and amide bioisosteres.^1^ Highlights of their use in medicinal chemistry programs include current investigations into their activity as anticancer,^2^ antimicrobial,^3^ and antiviral agents,^4^ and appearance in the antiretroviral raltegravir, whose sales revenue exceeded $850 million in 2020.^5^

Commonly, the synthesis of 2,5-disubstituted 1,3,4-oxadiazoles has depended on carboxylic acids as feedstocks to produce 1,2-diacyl hydrazines, or *N*-acyl hydrazones—synthetic precursors poised to undergo oxadiazole synthesis via dehydrative or oxidative methods.^6^ These approaches necessitate that the choice and installation of oxadiazole substituents precede oxadiazole formation, thereby limiting their synthetic versatility. In comparison, the C–H functionalization of monosubstituted 1,3,4-oxadiazoles is an alternative approach imparting flexibility; however, it requires synthesis and isolation of monosubstituted 1,3,4-oxadiazole starting materials, which themselves are predominantly derived from the above dehydrative or oxidative strategies.^7^ In the past decade, access to α-heteroatom 1,3,4-oxadiazoles has been expanded, with an accompanying reduction in step count, due to discovery of the efficient reactivity of the functionalized isocyanide *N*-isocyaniminotriphenylphosphorane (NIITP) with aldehydes,^8^ ketones,^9^ imines,^10^ and iminium ions (Scheme 1).^11^ However, the need for such C=X electrophiles to trigger downstream reactivity has limited the impact of NIITP for general 2,5-disubstituted 1,3,4-oxadiazole synthesis.^12^

**Scheme 1:**
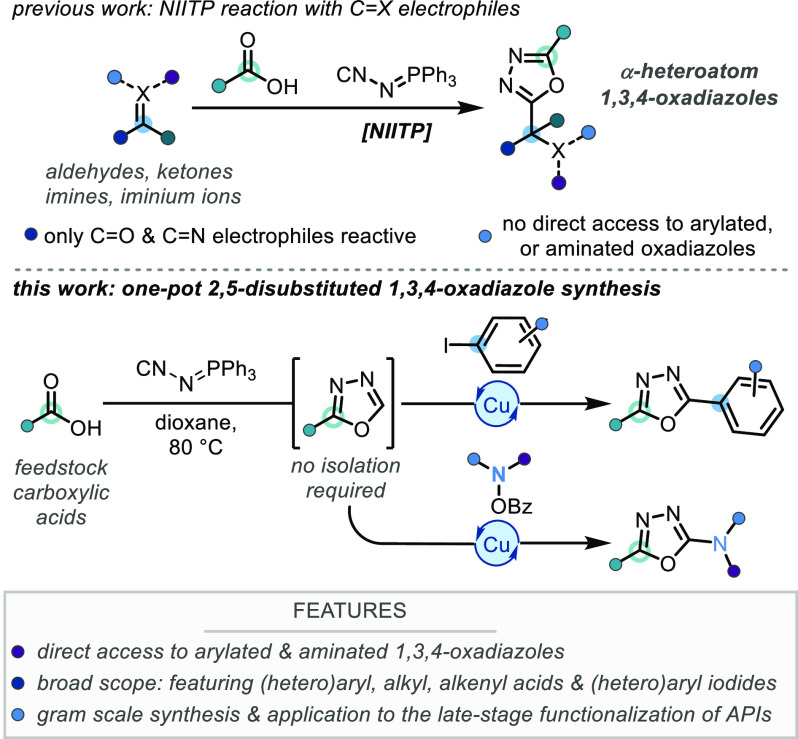
α-Heteroatom 1,3,4-Oxadiazole Synthesis Using NIITP and This Work

Building upon our recent work using NIITP for the synthesis of α-amino 1,3,4-oxadiazoles,^10c,11b^ a direct and general synthesis of 2,5-disubstituted 1,3,4-oxadiazoles from carboxylic acids and NIITP presented an attractive and unsolved challenge. We envisioned that a one-pot oxadiazole synthesis-functionalization strategy would be a synthetically empowering solution. Inspecting each step independently: first, a mild oxadiazole formation could be achieved using NIITP, and a carboxylic acid, with triphenylphosphine oxide as the only byproduct.^13^ Second, selecting a 1,3,4-oxadiazole functionalization method, C–H arylation using readily available aryl halides under copper catalysis, attracted our attention.^14^ Nevertheless, critical to the success of this C–H arylation step would be identification of a catalyst and ligand system that would tolerate the triphenylphosphine oxide generated in the first step, or residual NIITP. Finally, the combination of these two steps in sequence would allow for the judicious choice of both oxadiazole substituents to occur contemporaneously, from feedstock carboxylic acids and aryl iodides, greatly reducing the synthetic investment required from current approaches to 2,5-disubstituted 1,3,4-oxadiazoles. Herein we wish to report our findings.

Our investigation began with the optimization of the synthesis of monosubstituted 1,3,4-oxadiazole **1** from 4-fluorobenzoic acid, and NIITP (Scheme 2, entries 1–4). Focusing on delivering a practical two-stage, one-pot protocol, we decided to limit the reaction time for the oxadiazole synthesis step to 3 h to avoid unnecessary overnight reactions. Under previously reported conditions (entry 1) we found only 37% of **1** was afforded after this time.^13^ Changing the solvent to 1,4-dioxane, frequently used for 1,3,4-oxadiazole C–H functionalizations, had a deleterious effect on conversion (entry 2); however, an increase in the reaction temperature to 50 °C increased the conversion to 78% (entry 3). Further increasing the reaction temperature to 80 °C gave quantitative conversion to the desired monosubstituted 1,3,4-oxadiazole **1** after 3 h using only a small excess (1.1 equiv) of NIITP, with no side-products observed by ^19^F NMR analysis (entry 4). With the 1,3,4-oxadiazole synthesis step optimized, an oxadiazole C–H arylation using iodobenzene was added in sequence to study the efficacy of a one-pot two-stage protocol (entries 5–11).^14a^ Initially, a copper(I) iodide/1,10-phenanthroline catalytic system, at 50 mol %/100 mol % loading, was investigated, and we were encouraged to find that the desired product **2** was observed in 51% ^19^F NMR yield (entry 5), providing a firm proof-of-concept for our synthetic strategy. Increasing the loading of copper(I) iodide and ligand, to 100 and 200 mol %, respectively, resulted in diminished conversion and yield (entry 6). Further investigations revealed a loading of 20 mol % copper(I) iodide and 40 mol % 1,10-phenanthroline to be optimal (entry 7), with lower catalyst and ligand loadings giving reduced yields of **2** (entry 8). Finally, an evaluation of the equivalents of cesium carbonate used showed 1.5 equiv to be optimal (entries 9–11). These optimized conditions allowed for synthesis of 2,5-disubstituted 1,3,4-oxadiazole **2** directly from 4-fluorobenzoic acid, and iodobenzene, without isolation of the intermediate monosubstituted 1,3,4-oxadiazole **1**, in 78% isolated yield (entry 11).

**Scheme 2:**
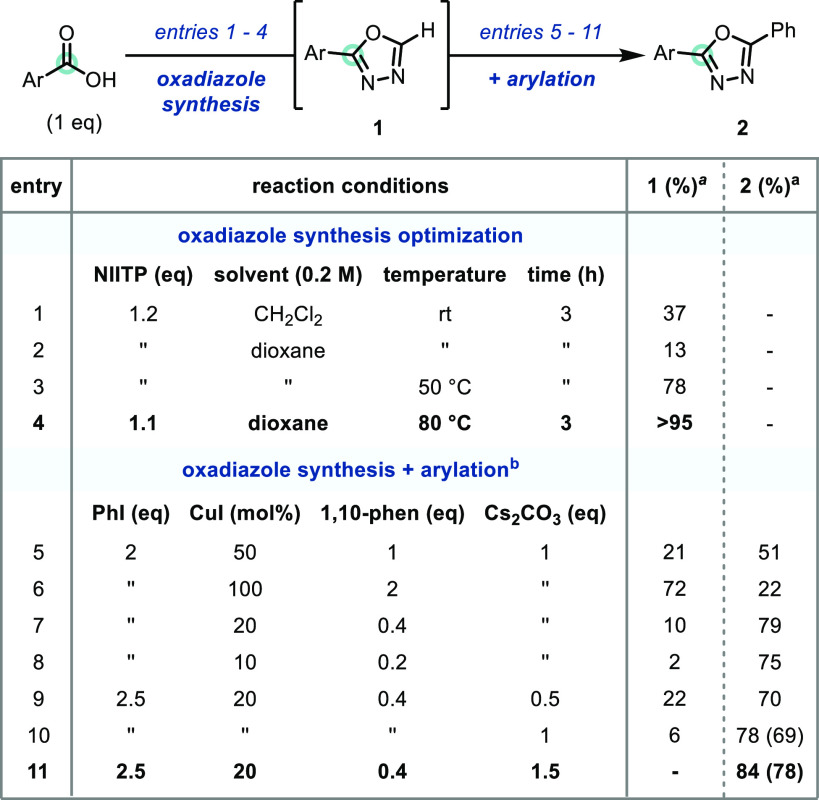
Reaction Discovery and Optimization ^19^F{^1^H} NMR yield determined by direct conversion between starting carboxylic acid, **1**, and **2** including byproducts; isolated yields in parentheses. Reaction conditions: 4-F-C_6_H_4_-CO_2_H (1 equiv), NIITP (1.1 equiv), dioxane (0.3 M, entries 5–8; or 0.4 M, entries 9–11), 80 °C, 3 h; then Phl, Cul, 1,10-Phen, Cs_2_CO_3_, dioxane (0.3 M, entries 5–8; or 0.2 M, entries 9–11), 110 °C, 16 h. One-pot 1,3,4-oxadlazole synthesis and arylation. Ar = 4-F-C_6_H_4_-.

Having established an efficient and practical oxadiazole synthesis-arylation protocol, the scope of the reaction was then investigated, and both carboxylic acid and aryl iodide components were varied in parallel to enhance the diversity of products generated (Scheme 3). Examining the scope of the aryl iodide coupling partner, we found that appended electron-donating groups such as a *tert*-butyl (**3**), and a methoxy group (**4**) gave good yields of the desired 1,3,4-oxadiazoles. Pleasingly, fluoro- (**5**), chloro- (**6**), and bromo-substituted (**7**) aryl iodides did not undergo competitive dehalogenative side-reactions, allowing the products to have aryl halide handles for further functionalization. The arylation reaction was insensitive to steric effects with 2-iodotoluene reacting to give an excellent 82% yield of 1,3,4-oxadiazole **8**. Electron-withdrawing substituents such as a trifluoromethyl (**9**), or an ethyl ester (**10**) group were also well tolerated under the optimized reaction conditions. Furthermore, the incorporation of heteroaryl motifs, essential to medicinal chemistry programs,^15^ was explored, and revealed that all regioisomers of iodopyridine could be readily coupled and integrated into the products **11**–**13**. Continuing our exploration of aryl iodides with diverse substitutions, we found that a range of coupling partners including 4-iodobenzonitrile (**14**), 3,5-dimethylbenzene (**15**), 2-methoxyiodobenzene (**16**), 1-iodonaphthalene (**17**), 2-iodonaphthalene (**18**), and 3-methoxyiodobenzene (**19**) underwent successful couplings, giving good (60%) to excellent (80%) yields of the desired 2,5-disubstituted 1,3,4-oxadiazoles.

**Scheme 3:**
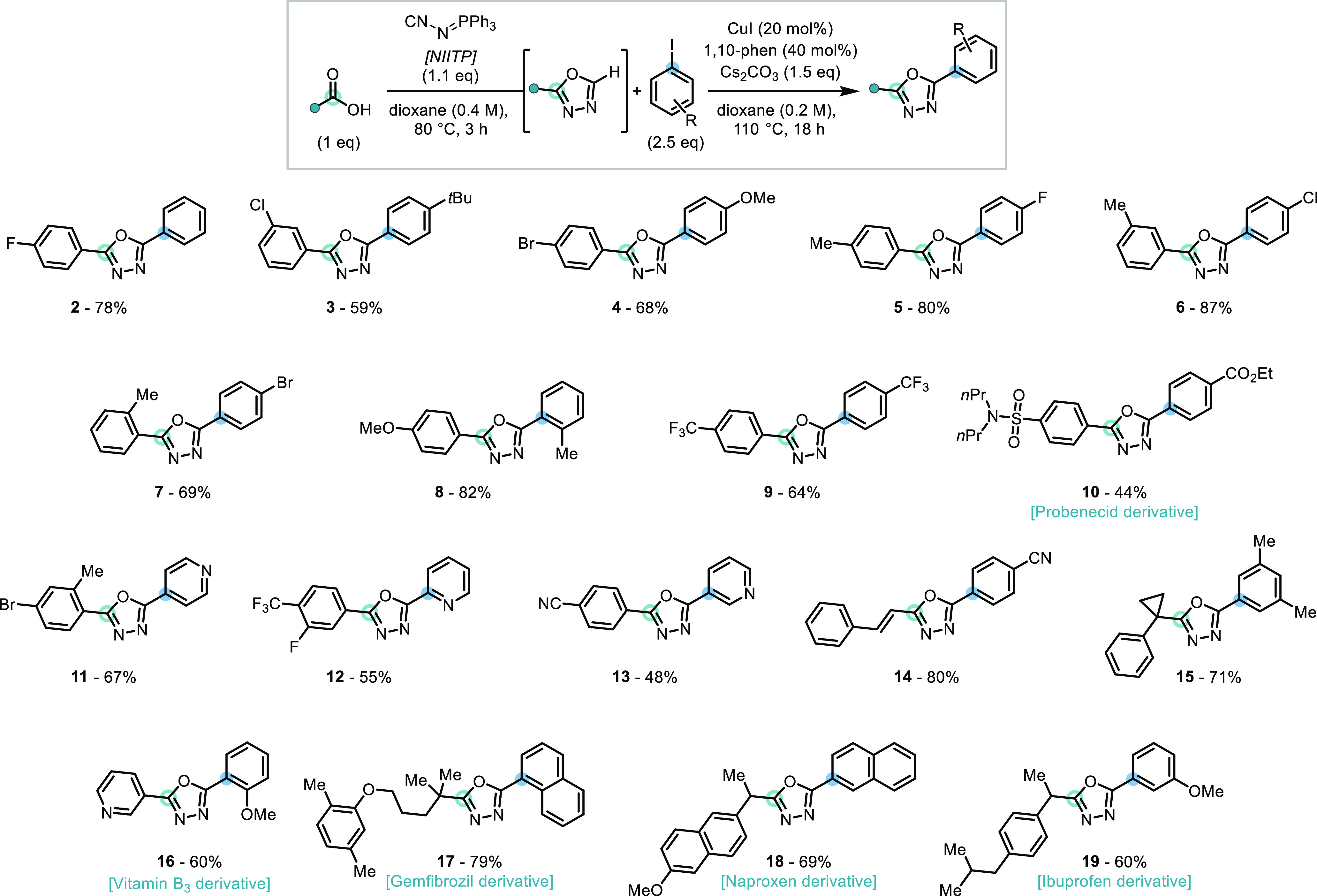
Reaction Scope Isolated yields (0.20 mmol scale).

Focusing on the scope with respect to the carboxylic acid coupling partner, we found that fluoro- (**2**), chloro- (**3**), and bromo-containing carboxylic acids (**4**) gave good yields of the desired products. The reaction’s sensitivity to steric effects was examined using 4- (**5**), 3- (**6**), and 2-methylbenzoic acid (**7**) coupling partners and resulted in 2,5-disubstituted 1,3,4-oxadiazole products **5**–**7** being synthesized in 69–87% yield. Carboxylic acids containing either a strongly electron-donating methoxy group (**8**), or an electron-withdrawing trifluoromethyl group (**9**) were found to react productively. The carboxylic acid API probenecid was subjected to the protocol and gave the sulfonamide-containing 1,3,4-oxadiazole **10** in 44% yield.^16^ The disubstituted carboxylic acids 4-bromo-2-methylbenzoic acid (**11**) and 3-fluoro-4-(trifluoromethyl)benzoic acid (**12**) could be successfully employed yielding products with complex-substitution patterns. The use of 4-cyanobenzoic acid (**13**) further showcased the reaction’s tolerance to electron-withdrawing groups. Venturing away from aromatic carboxylic acids, we were pleased to find that simple alkenyl (**14**) and alkyl (**15**) carboxylic acids gave excellent product yields, as these substrates classes are routinely underrepresented in 1,3,4-oxadiazole C–H functionalization methodologies.^7,14^ Finally, the late-stage functionalization of the APIs Vitamin B_3_ (**16**), which features a heteroaryl carboxylic acid, gemfibrozil (**17**), naproxen (**18**), and ibuprofen (**19**) was successfully performed and gave diverse 1,3,4-oxadiazole products in good yields, using the developed one-pot protocol.

Translation of the newly developed reaction sequence into a preparative gram-scale synthesis of **6** was realized, in 74% yield, highlighting its efficiency and attractiveness for 2,5-disubstituted 1,3,4-oxadiazole synthesis, from commercially available starting materials (Scheme 4A).

**Scheme 4:**
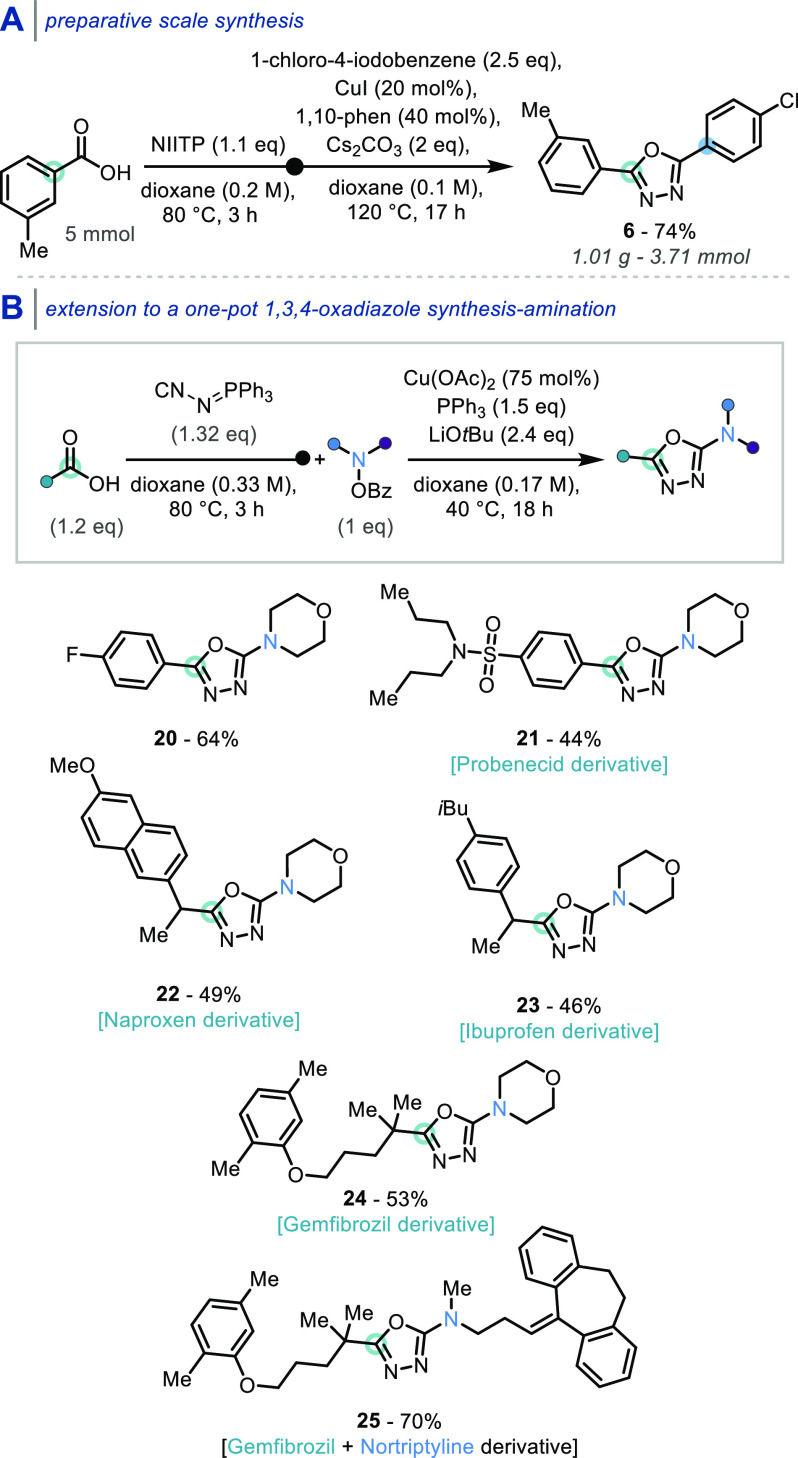
(A) Preparative Scale Synthesis Example; (B) Extension to a One-Pot 1,3,4-Oxadiazole Synthesis-Amination Isolated yields (0.17 mmol scale).

With a practical and scalable one-pot oxadiazole synthesis-arylation protocol established, the versatility of the strategy was further demonstrated by its extension to a one-pot synthesis of aminated 1,3,4-oxadiazoles (Scheme 4B). The desired transformation could indeed be achieved using a *N*-benzoyloxy amine as an electrophilic aminating reagent, in combination with copper(II) acetate, as previously detailed by Hirano and Miura.^17^ Merging this 1,3,4-oxadiazole C–H amination system with our optimized oxadiazole synthesis conditions, allowed for the one-pot synthesis of the aminated 1,3,4-oxadiazole **20** from 4-fluorobenzoic acid, and *N*-(benzoyloxy)morpholine, in 64% yield. The identified one-pot oxadiazole synthesis-amination protocol was applied to the late-stage functionalization of the APIs probenecid (**21**), naproxen (**22**), ibuprofen (**23**), and gemfibrozil (**24**), which feature both aryl and alkyl carboxylic acids, leading to the successful synthesis of compounds **21**–**24** from these APIs. Furthermore, the late-stage functionalization of both the carboxylic acid API gemfibrozil and the secondary amine API nortipyline, using its readily prepared *N*-benzoyloxy derivative,^18^ was performed and provided the 1,3,4-oxadiazole-fused drug–drug conjugate **25** in a 70% yield. Notably, this approach offers a reduced step-count compared to previous methods for the synthesis of aminated 1,3,4-oxadiazoles, which require the synthesis and isolation of monosubstituted 1,3,4-oxadiazole starting materials.^19^

In conclusion, a one-pot, two stage oxadiazole synthesis-functionalization protocol allowing for the synthesis of diverse 2,5-disubstituted 1,3,4-oxadiazoles from feedstock carboxylic acids, NIITP, and aryl iodide or *N*-benzyloxy amine coupling partners has been developed. The scope of the arylative method was found to be broad with respect to both the carboxylic acid and aryl iodide coupling partners, and included the successful utilization of heteroaromatic acids and iodides—desirable motifs in medicinal chemistry efforts. The application of the arylative and aminative protocols to the late-stage functionalization of carboxylic acid containing APIs was demonstrated and featured the synthesis of a 1,3,4-oxadiazole-fused drug–drug conjugate of gemfibrozil and nortipyline. The presented strategy significantly reduces the step-count of traditional approaches, which we believe will greatly enhance the accessibility of 2,5-disubstituted 1,3,4-oxadiazoles. Continuing work to uncover new broad scope access to 1,3,4-oxadiazole motifs is ongoing in our laboratory, and the results will be disclosed in due course.

## Experimental Section

### General Information

Reactions were carried out under a nitrogen atmosphere unless stated otherwise. Glassware was oven-dried and cooled under a vacuum and then purged with nitrogen before use. Room temperature refers to 22 ± 2 °C. Reactions carried out at high temperatures were heated using an oil bath. Reaction temperatures refer to external temperatures of an oil bath.

### Nomenclature and Numbering

Compounds are named following IUPAC nomenclature as generated by ChemDraw.

### Solvents

Anhydrous solvents were either obtained from Sure/Seal bottles purchased from Sigma-Aldrich, or an MBRAUN-SPS solvent purification system in which solvent is passed through an activated alumina column under nitrogen. Reagents were used as obtained without further purification unless stated otherwise.

### Chromatography

Thin layer chromatography (TLC) was carried out using Merck aluminum backed DC60 F254 plates (particle size 0.2 mm). TLC sheets were visualized by UV light, and then developed by staining with potassium permanganate. Purification by flash column chromatography (FCC) was carried out using Merck silica gel 60 F254 (particle size 43–60 μm).

### Characterization

Proton (^1^H), fluorine (^19^F), and carbon (^13^C) spectra were recorded on Bruker AVANCE NEO600, Bruker AVG400, Bruker AVH400, Bruker AVF400, Bruker AVB500, Bruker AVC500, and Bruker DPX200 NMR spectrometers. Spectra are referenced to the residual solvent peak. Chemical shifts (δ) are given in parts per million (ppm, ±0.01), and coupling constants (*J*) are given in Hertz (Hz, ±0.1 as measured on Mestrenova, without rounding). The following convention is used to report chemical shifts: δ (multiplicity, coupling constant(s), number of protons), with chemical shifts reported in descending order. Peak multiplicities are described as singlet (s), doublet (d), triplet (t), quartet (q), pentet (p), heptet (h), nonuplet (n), a combination, e.g., doublet of doublets (dd), or as a multiplet (m) over a peak range. Infrared spectra were recorded using a Bruker Tensor 27 FT-IR spectrometer. Selected diagnostic absorption maxima (ν_max_) are reported in wavenumbers (cm^–1^). High resolution mass spectra were recorded by Chemistry Research Laboratory staff using a Bruker Daltronics MicroTOF spectrometer (ESI). Mass to charge ratios (*m*/*z*) are reported in Daltons. Melting points were recorded using a Leica Galen III hot-stage microscope apparatus and are reported uncorrected in degrees Celsius (°C).

### Starting Materials

Carboxylic acids and aryl iodides were obtained from commercial chemical suppliers and used as received. Copper(I) iodide (43153, Puratronic, 99.998% metal basis) and copper(II) acetate (44355, 99.999% metal basis) were purchased from Alfa Aesar and used as received. (*N*-Isocyanimino) triphenylphosphorane (NIITP) was synthesized according to Bio’s method.^20^ Morpholino benzoate was synthesized according to a literature procedure.^21^

### General Procedure A: One-Pot 1,3,4-Oxadiazole Synthesis-Arylation (**2**–**19**)

To a dry Schlenk tube under nitrogen was added carboxylic acid (0.20 mmol, 1.0 equiv) and NIITP (66.5 mg, 0.22 mmol, 1.1 equiv). The Schlenk tube was then evacuated and backfilled with nitrogen (×4), and then anhydrous 1,4-dioxane (0.50 mL, 0.40 M) was added. The Schlenk tube was then sealed and put into an oil bath preheated at 80 °C and stirred for 3 h. After this time the reaction was cooled to rt and aryl iodide (0.50 mmol, 2.5 equiv), 1,10-phenanthroline (14.4 mg, 0.08 mmol, 40 mol %), cesium carbonate (97.7 mg, 0.30 mmol, 1.5 equiv), copper(I) iodide (7.6 mg, 0.04 mmol, 20 mol %), and anhydrous 1,4-dioxane (0.50 mL, 0.20 M total) were added sequentially. The Schlenk tube was then sealed and put into an oil bath preheated at 110 °C and stirred for 18 h. After this time the reaction was cooled to rt and filtered through a silica plug, washing with EtOAc, and then concentrated in vacuo to afford the crude product. The crude product was purified by flash column chromatography (FCC), and subsequent preparative thin-layer chromatography (PTLC) when required, to afford the pure 2,5-disubstituted 1,3,4-oxadiazole product.

#### 2-(4-Fluorophenyl)-5-phenyl-1,3,4-oxadiazole (**2**)

Following general procedure A (using 4-fluorobenzoic acid (28.0 mg, 0.20 mmol, 1.0 equiv), and iodobenzene (56 μL, 0.50 mmol, 2.5 equiv)): after FCC (20% Et_2_O/Pentane), **2** (37.3 mg, 0.156 mmol, 78%) was afforded as a white solid. mp 154–156 °C; ^1^H NMR (400 MHz, CDCl_3_) δ 8.19–8.09 (m, 4H), 7.60–7.50 (m, 3H), 7.28–7.19 (m, 2H); ^19^F NMR (376 MHz, CDCl_3_) δ −106.8. ^13^C{^1^H} NMR (101 MHz, CDCl_3_) δ 164.8 (d, *J* = 253.2), 164.6, 163.8, 131.8, 129.2 (d, *J* = 8.9), 129.1, 126.9, 123.8, 120.3 (*J* = 3.6), 116.5 (d, *J* = 22.3). Data were consistent with that found in the literature.^14a^

#### 2-(4-(*tert*-Butyl)phenyl)-5-(3-chlorophenyl)-1,3,4-oxadiazole (**3**)

Following general procedure A (using 3-chlorobenzoic acid (31.3 mg, 0.20 mmol, 1.0 equiv), and 1-(*tert*-butyl)-4-iodobenzene (89 μL, 0.50 mmol, 2.5 equiv)): after FCC (20% Et_2_O/Pentane), **3** (36.6 mg, 0.118 mmol, 59%) was afforded as a beige powder. mp 112–114 °C; ^1^H NMR (400 MHz, CDCl_3_) δ 8.11 (t, *J* = 1.6, 1H), 8.08–8.01 (m, 3H), 7.58–7.53 (m, 2H), 7.53–7.49 (m, 1H), 7.49–7.44 (m, 1H), 1.37 (s, 9H); ^13^C{^1^H} NMR (101 MHz, CDCl_3_) δ 165.0, 163.2, 155.6, 135.2, 131.6, 130.4, 126.9, 126.8, 126.1, 125.7, 125.0, 120.8, 35.1, 31.1; IR ν_max_/cm^–1^ 2964, 1615, 1575, 1546, 1494, 1413, 1271, 1018, 839, 749, 724; (ESI-TOF) *m*/*z* [M + H]^+^ calcd for C_18_H_18_ON_2_^35^Cl 313.1102, found 313.1104.

#### 2-(4-Bromophenyl)-5-(4-methoxyphenyl)-1,3,4-oxadiazole (**4**)

Following general procedure A (using 4-bromobenzoic acid (40.2 mg, 0.20 mmol, 1.0 equiv), and 1-iodo-4-methoxybenzene (117 mg, 0.50 mmol, 2.5 equiv)): after FCC (40% → 50% Et_2_O/Pentane), **4** (44.8 mg, 0.136 mmol, 68%) was afforded as a beige powder. mp 144–146 °C; ^1^H NMR (400 MHz, CDCl_3_) δ 8.08–8.01 (m, 2H), 7.99–7.94 (m, 2H), 7.68–7.62 (m, 2H), 7.04–6.98 (m, 2H), 3.87 (s, 3H); ^13^C{^1^H} NMR (101 MHz, CDCl_3_) δ 164.7, 163.4, 162.5, 132.4, 128.7, 128.2, 126.2, 123.0, 116.2, 114.6, 55.5; IR ν_max_/cm^–1^ 2917, 1611, 1495, 1477, 1258, 1172, 1074, 832, 723; (ESI-TOF) *m*/*z* [M + H]^+^ calcd for C_15_H_12_O_2_N_2_^79^Br 331.0077, found 331.0078.

#### 2-(4-Fluorophenyl)-5-(*p*-tolyl)-1,3,4-oxadiazole (**5**)

Following general procedure A (using 4-methylbenzoic acid (27.2 mg, 0.20 mmol, 1.0 equiv), and 1-fluoro-4-iodobenzene (58 μL, 0.50 mmol, 2.5 equiv)): after FCC (25% Et_2_O/Pentane), **5** (40.6 mg, 0.160 mmol, 80%) was afforded as a white powder. mp 166–168 °C; ^1^H NMR (400 MHz, CDCl_3_) δ 8.16–8.09 (m, 2H), 7.99 (d, *J* = 8.0, 2H), 7.32 (d, *J* = 8.0, 2H), 7.24–7.17 (m, 2H), 2.43 (s, 3H); ^19^F NMR (376 MHz, CDCl_3_) δ −107.0; ^13^C{^1^H} NMR (101 MHz, CDCl_3_) δ 164.8, 164.7 (d, *J* = 253.1), 163.5, 142.4, 129.8, 129.1 (d, *J* = 8.9), 126.9, 121.1, 120.4 (d, *J* = 3.4), 116.4 (d, *J* = 22.3), 21.7; IR ν_max_/cm^–1^ 1608, 1494, 1418, 1227, 1159, 1096, 1069, 1013, 843, 824, 740; (ESI-TOF) *m*/*z* [M + H]^+^ calcd for C_15_H_12_ON_2_F 255.0928, found 255.0929.

#### 2-(4-Chlorophenyl)-5-(*m*-tolyl)-1,3,4-oxadiazole (**6**)

Following general procedure A (using 3-methylbenzoic acid (27.2 mg, 0.20 mmol, 1.0 equiv), and 1-chloro-4-iodobenzene (119 mg, 0.50 mmol, 2.5 equiv)): after FCC (25% Et_2_O/Pentane), **6** (47.0 mg, 0.174 mmol, 87%) was afforded as a beige powder. 5 mmol scale procedure: To an oven-dried round-bottom flask (rbf) was added 3-methylbenzoic acid (681 mg, 5.0 mmol, 1.0 equiv), and NIITP (1.67 g, 5.5 mmol, 1.1 equiv). The rbf was then evacuated and backfilled with nitrogen (×4) before the addition of anhydrous 1,4-dioxane (25 mL, 0.20 M). The rbf was put into an oil bath, preheated to 80 °C, and stirred for 3 h. After this time the reaction was cooled to rt and anhydrous 1,4-dioxane (15 mL, 0.14 M total), 1-chloro-4-iodobenzene (2.98 g, 12.5 mmol, 2.5 equiv), 1,10-phenanthroline (360 mg, 2.0 mmol, 40 mol %), cesium carbonate (2.44 g, 7.5 mmol, 1.5 equiv), copper(I) iodide (190 mg, 1.0 mmol, 20 mol %), and anhydrous 1,4-dioxane (10 mL, 0.10 M total) were added sequentially. The rbf was then put into an oil bath preheated at 120 °C and stirred for 17 h. After this time, the reaction was cooled to rt and filtered through a silica plug, washing with EtOAc (200 mL), and then concentrated in vacuo to afford the crude product. The crude product was purified by FCC (25% → 35% Et_2_O/pentane) to afford the **6** (1.01 g, 3.71 mmol, 74%) as a beige powder. mp 130–132 °C; ^1^H NMR (400 MHz, CDCl_3_) δ 8.09–8.02 (m, 2H), 7.94–7.91 (m, 1H), 7.90 (d, *J* = 7.6, 1H), 7.51–7.46 (m, 2H), 7.40 (t, *J* = 7.6, 1H), 7.34 (d, *J* = 7.6, 1H), 2.44 (s, 3H); ^13^C{^1^H} NMR (101 MHz, CDCl_3_) δ 164.9, 163.7, 139.0, 137.9, 132.7, 129.5, 129.0, 128.2, 127.5, 124.1, 123.6, 122.5, 21.3; IR ν_max_/cm^–1^ 1597, 1543, 1481, 1405, 1089, 1012, 908, 839, 790, 734, 687; (ESI-TOF) *m*/*z* [M + H]^+^ calcd for C_15_H_12_O_2_N^35^Cl 271.0633, found 271.0633.

#### 2-(4-Bromophenyl)-5-(*o*-tolyl)-1,3,4-oxadiazole (**7**)

Following general procedure A (using 2-methylbenzoic acid (27.2 mg, 0.20 mmol, 1.0 equiv), and 1-bromo-4-iodobenzene (141 mg, 0.50 mmol, 2.5 equiv)): after FCC (15% Et_2_O/Pentane), **7** (43.2 mg, 0.138 mmol, 69%) was afforded as a white powder. mp 138–140 °C; ^1^H NMR (400 MHz, CDCl_3_) δ 8.04–7.96 (m, 3H), 7.70–7.64 (m, 2H), 7.46–7.40 (m, 1H), 7.39–7.31 (m, 2H), 2.76 (s, 3H); ^13^C{^1^H} NMR (101 MHz, CDCl_3_) δ 165.0, 163.4, 138.5, 132.5, 131.9, 131.4, 129.0, 128.3, 126.4, 126.2, 122.9, 122.8, 22.1; IR ν_max_/cm^–1^ 2980, 1600, 1538, 1452, 1097, 1068, 1009, 959, 730; (ESI-TOF) *m*/*z* [M + H]^+^ calcd for C_15_H_12_ON_2_^79^Br 315.0128, found 315.0128.

#### 2-(4-Methoxyphenyl)-5-(*o*-tolyl)-1,3,4-oxadiazole (**8**)

Following general procedure A (using 4-methoxybenzoic acid (30.4 mg, 0.20 mmol, 1.0 equiv), and 1-iodo-2-methylbenzene (64 μL, 0.50 mmol, 2.5 equiv)): after FCC (30% → 50% Et_2_O/Pentane), **8** (43.8 mg, 0.164 mmol, 82%) was afforded as a white powder. mp 118–120 °C; ^1^H NMR (400 MHz, CDCl_3_) δ 8.09–8.03 (m, 2H), 8.03–7.99 (m, 1H), 7.45–7.37 (m, 1H), 7.37–7.29 (m, 2H), 7.06–6.98 (m, 2H), 3.87 (s, 3H), 2.75 (s, 3H); ^13^C{^1^H} NMR (101 MHz, CDCl_3_) δ 164.4, 164.1, 162.3, 138.3, 131.8, 131.0, 128.9, 128.7, 126.1, 123.2, 116.5, 114.5, 55.5, 22.1; IR ν_max_/cm^–1^ 1613, 1502, 1260, 1253, 1020, 844, 798, 723; (n-TOF) *m*/*z* [M + H]^+^ calcd for C_16_H_15_O_2_N_2_ 267.1128, found 267.1126.

#### 2,5-Bis(4-(trifluoromethyl)phenyl)-1,3,4-oxadiazole (**9**)

Following general procedure A (using 4-(trifluoromethyl)benzoic acid (38.0 mg, 0.20 mmol, 1.0 equiv), and 1-iodo-4-(trifluoromethyl)benzene (73 μL, 0.50 mmol, 2.5 equiv)): after FCC (12% Et_2_O/Pentane), **9** (45.9 mg, 0.128 mmol, 64%) was afforded as a white powder. mp 150 °C (decomp); ^1^H NMR (400 MHz, CDCl_3_) δ 8.27 (d, *J* = 8.1, 4H), 7.81 (d, *J* = 8.2, 4H); ^19^F NMR (376 MHz, CDCl_3_) δ −63.2; ^13^C{^1^H} NMR (101 MHz, CDCl_3_) δ 164.0, 133.7 (q, *J* = 33.0), 127.4, 126.8, 126.2 (q, *J* = 3.7), 123.5 (q, *J* = 272.5); IR ν_max_/cm^–1^ 1324, 1317, 1169, 1137, 1105, 1083, 1063, 1015, 850, 755, 713, 667; (ESI-TOF) *m*/*z* [M + H]^+^ calcd for C_16_H_9_ON_2_F_6_ 359.0614, found 359.0611.

#### Ethyl 4-(5-(4-(*N*,*N*-dipropylsulfamoyl)phenyl)-1,3,4-oxadiazol-2-yl)benzoate (**10**)

Following general procedure A (using probenecid (57.1 mg, 0.20 mmol, 1.0 equiv), and ethyl 4-iodobenzoate (84 μL, 0.50 mmol, 2.5 equiv)): after FCC (20% → 30% EtOAc/Pentane), **10** (39.8 mg, 0.088 mmol, 44%) was afforded as a yellow crystalline solid. mp 164–166 °C; ^1^H NMR (400 MHz, CDCl_3_) δ 8.31–8.25 (m, 2H), 8.21 (s, 4H), 8.01–7.94 (m, 2H), 4.42 (q, *J* = 7.1, 2H), 3.18–3.08 (m, 4H), 1.56 (h, *J* = 7.4, 4H), 1.42 (t, *J* = 7.1, 3H), 0.87 (t, *J* = 7.4, 6H); ^13^C{^1^H} NMR (101 MHz, CDCl_3_) δ 165.5, 164.5, 163.8, 143.5, 133.6, 130.3, 127.8, 127.6, 127.1, 127.0, 61.6, 49.9, 21.9, 14.3, 11.2; IR ν_max_/cm^–1^ 2969, 1718, 1341, 1273, 1158, 1074, 688, 612; (ESI-TOF) *m*/*z* [M + H]^+^ calcd for C_23_H_28_O_5_N_3_S 458.1744, found 458.1742.

#### 2-(4-Bromo-2-methylphenyl)-5-(pyridin-4-yl)-1,3,4-oxadiazole (**11**)

Following general procedure A (using 4-bromo-2-methylbenzoic acid (42.6 mg, 0.20 mmol, 1.0 equiv), and 4-iodopyridine (103 mg, 0.50 mmol, 2.5 equiv)): after FCC (20% → 30% Acetone/Pentane), **11** (42.4 mg, 0.134 mmol, 67%) was afforded as a white powder. mp 180–182 °C; ^1^H NMR (400 MHz, CDCl_3_) δ 8.86–8.79 (m, 2H), 7.98–7.92 (m, 2H), 7.88 (d, *J* = 8.4, 1H), 7.56–7.53 (m, 1H), 7.49 (dd, *J* = 8.4, 1.9, 1H), 2.73 (s, 3H); ^13^C{^1^H} NMR (101 MHz, CDCl_3_) δ 165.1, 162.4, 151.0, 140.7, 134.9, 130.8, 130.3, 129.6, 126.4, 121.4, 120.3, 22.0; IR ν_max_/cm^–1^ 2980, 1596, 1539, 1478, 1413, 827, 743, 704; (ESI-TOF) *m*/*z* [M + H]^+^ calcd for C_14_H_11_ON_2_^79^Br 316.0080, found 316.0081.

#### 2-(3-Fluoro-4-(trifluoromethyl)phenyl)-5-(pyridin-2-yl)-1,3,4-oxadiazole (**12**)

Following general procedure A (using 3-fluoro-4-(trifluoromethyl)benzoic acid (41.6 mg, 0.20 mmol, 1.0 equiv), and 2-iodopyridine (53 μL, 0.50 mmol, 2.5 equiv)): after FCC (40% EtOAc/Pentane), **12** (34.3 mg, 0.110 mmol, 55%) was afforded as a beige powder. mp 156–158 °C; ^1^H NMR (400 MHz, CDCl_3_) δ 8.82 (ddd, *J* = 4.8, 1.7, 0.9, 1H), 8.33 (dt, *J* = 7.9, 1.0, 1H), 8.11 (d, *J* = 8.2, 1H), 8.06 (d, *J* = 10.5, 1H), 7.92 (td, *J* = 7.8, 1.7, 1H), 7.78 (t, *J* = 7.6, 1H), 7.51 (ddd, *J* = 7.7, 4.8, 1.2, 1H); ^19^F NMR (376 MHz, CDCl_3_) δ −61.71 (d, *J* = 12.8, 3F), −111.95 to −112.13 (m, 1F); ^13^C{^1^H} NMR (101 MHz, CDCl_3_) δ 164.6, 163.4 (d, *J* = 3.1), 159.9 (d, *J* = 256.3), 150.5, 143.1, 137.4, 129.1 (d, *J* = 9.0), 128.3 (dd, *J* = 4.7, 2.0), 126.3, 123.6, 122.8 (d, *J* = 4.0), 122.0 (d, *J* = 272.5), 115.7 (d, *J* = 23.8); IR ν_max_/cm^–1^ 1556, 1444, 1324, 1127, 1046, 658, 741, 728; (ESI-TOF) *m*/*z* [M + H]^+^ calcd for C_14_H_8_ON_3_F_4_ 310.0598, found 310.0598.

#### 4-(5-(Pyridin-3-yl)-1,3,4-oxadiazol-2-yl)benzonitrile (**13**)

Following general procedure A (using 4-cyanobenzoic acid (29.4 mg, 0.20 mmol, 1.0 equiv), and 3-iodopyridine (103 mg, 0.50 mmol, 2.5 equiv)): after FCC (30% Acetone/Pentane), **13** (24.0 mg, 0.096 mmol, 48%) was afforded as a beige powder. mp 182–184 °C; ^1^H NMR (400 MHz, CDCl_3_) δ 9.37–9.33 (m, 1H), 8.82 (dd, *J* = 4.9, 1.6, 1H), 8.44 (dt, *J* = 8.0, 2.0, 1H), 8.30–8.24 (m, 2H), 7.89–7.82 (m, 2H), 7.51 (ddd, *J* = 8.0, 4.9, 0.8, 1H); ^13^C{^1^H} NMR (101 MHz, CDCl_3_) δ 163.6, 163.3, 152.9, 148.0, 134.3, 133.0, 127.5, 127.4, 123.9, 120.0, 117.8, 115.6; IR ν_max_/cm^–1^ 2231, 1601, 1493, 1410, 1273, 852, 741, 718, 702; (ESI-TOF) *m*/*z* [M + H]^+^ calcd for C_14_H_9_ON_4_ 249.0771, found 249.0770.

#### (*E*)-4-(5-Styryl-1,3,4-oxadiazol-2-yl)benzonitrile (**14**)

Following general procedure A (using cinnamic acid (29.6 mg, 0.20 mmol, 1.0 equiv), and 4-iodobenzonitrile (115 mg, 0.50 mmol, 2.5 equiv)): after FCC (20% → 30% EtOAc/Pentane), **14** (43.8 mg, 0.160 mmol, 80%) was afforded as a beige powder. mp 184–186 °C; ^1^H NMR (400 MHz, CDCl_3_) δ 8.26–8.18 (m, 2H), 7.85–7.77 (m, 2H), 7.66 (d, *J* = 16.5, 1H), 7.60–7.54 (m, 2H), 7.47–7.37 (m, 3H), 7.08 (d, *J* = 16.5, 1H); ^13^C{^1^H} NMR (101 MHz, CDCl_3_) δ 165.0, 162.5, 140.1, 134.5, 132.9, 130.4, 129.1, 127.73, 127.67, 127.4, 117.9, 115.2, 109.4; IR ν_max_/cm^–1^ 3062, 2228, 1642, 1520, 1490, 1087, 1015, 966, 842, 751, 694, 684; (ESI-TOF) *m*/*z* [M + H]^+^ calcd for C_17_H_12_ON_3_ 274.0975, found 274.0975.

#### 2-(3,5-Dimethylphenyl)-5-(1-phenylcyclopropyl)-1,3,4-oxadiazole (**15**)

Following general procedure A (using 1-phenyl-1-cyclopropanecarboxylic acid (32.4 mg, 0.20 mmol, 1.0 equiv), and 1-iodo-3,5-dimethylbenzene (72 μL, 0.50 mmol, 2.5 equiv)): after FCC (30% Et_2_O/Pentane), **15** (41.3 mg, 0.142 mmol, 71%) was afforded as a yellow oil. ^1^H NMR (400 MHz, CDCl_3_) δ 7.59–7.55 (m, 2H), 7.50–7.44 (m, 2H), 7.41–7.35 (m, 2H), 7.34–7.29 (m, 1H), 7.13–7.09 (m, 1H), 2.35 (s, 6H), 1.79–1.74 (m, 2H), 1.51–1.46 (m, 2H); ^13^C{^1^H} NMR (101 MHz, CDCl_3_) δ 169.3, 165.0, 138.9, 138.6, 133.2, 129.5, 128.7, 127.7, 124.5, 123.8, 22.4, 21.2, 16.0; IR ν_max_/cm^–1^ 2918, 1569, 1549, 1447, 1160, 1033, 1024, 857, 743, 698; (ESI-TOF) *m*/*z* [M + H]^+^ calcd for C_19_H_19_ON_2_ 291.1492, found 291.1489.

#### 2-(2-Methoxyphenyl)-5-(pyridin-3-yl)-1,3,4-oxadiazole (**16**)

Following general procedure A (using nicotinic acid (24.6 mg, 0.20 mmol, 1.0 equiv), and 1-iodo-2-methoxybenzene (65 μL, 0.50 mmol, 2.5 equiv)): after FCC (30% Acetone/Pentane) and PTLC (50% EtOAc/CHCl_3_), **16** (30.6 mg, 0.120 mmol, 60%) was afforded as a white crystalline solid. mp 114–116 °C; ^1^H NMR (400 MHz, CDCl_3_) δ 9.34 (d, *J* = 1.6, 1H), 8.76 (dd, *J* = 4.9, 1.6, 1H), 8.42 (dt, *J* = 8.0, 1.9, 1H), 8.02 (dd, *J* = 7.7, 1.7, 1H), 7.53 (ddd, *J* = 8.4, 7.5, 1.8, 1H), 7.47 (ddd, *J* = 8.0, 4.9, 0.7, 1H), 7.13–7.04 (m, 2H), 3.99 (s, 3H); ^13^C{^1^H} NMR (101 MHz, CDCl_3_) δ 164.0, 162.3, 158.0, 152.2, 147.9, 134.1, 133.4, 130.6, 123.8, 120.8, 120.7, 112.7, 112.1, 56.1; IR ν_max_/cm^–1^ 2971, 1604, 1588, 1496, 1475, 1287, 1264, 1022, 749, 721, 704; (ESI-TOF) *m*/*z* [M + H]^+^ calcd for C_14_H_12_O_2_N_3_ 254.0924, found 254.0924.

#### 2-(5-(2,5-Dimethylphenoxy)-2-methylpentan-2-yl)-5-(naphthalen-1-yl)-1,3,4-oxadiazole (**17**)

Following general procedure A (using 5-(2,5-dimethylphenoxy)-2,2-dimethylpentanoic acid (gemfibrozil) (50.1 mg, 0.20 mmol, 1.0 equiv), and 1-iodonaphthalene (73 μL, 0.50 mmol, 2.5 equiv)): after FCC (20% Et_2_O/Pentane), **17** (63.0 mg, 0.158 mmol, 79%) was afforded as a yellow oil. ^1^H NMR (400 MHz, CDCl_3_) δ 9.24 (d, *J* = 8.7, 1H), 8.15 (dd, *J* = 7.3, 1.2, 1H), 8.03 (d, *J* = 8.2, 1H), 7.94 (d, *J* = 8.1, 1H), 7.73–7.66 (m, 1H), 7.64–7.53 (m, 2H), 6.98 (d, *J* = 7.5, 1H), 6.63 (d, *J* = 7.5, 1H), 6.60 (s, 1H), 3.96 (t, *J* = 6.1, 2H), 2.28 (s, 3H), 2.17 (s, 3H), 2.10–2.02 (m, 2H), 1.91–1.81 (m, 2H), 1.60 (s, 6H); ^13^C{^1^H} NMR (101 MHz, CDCl_3_) δ 171.9, 164.8, 156.9, 136.5, 133.9, 132.4, 130.3, 130.1, 128.7, 128.2, 128.1, 126.7, 126.3, 124.8, 123.5, 120.8, 111.9, 67.6, 38.1, 35.8, 26.3, 25.0, 21.4, 15.8; IR ν_max_/cm^–1^ 2972, 1537, 1509, 1264, 1157, 1126, 1047, 805, 775; (ESI-TOF) *m*/*z* [M + H]^+^ calcd for C_26_H_29_O_2_N_2_ 401.2224, found 401.2235.

#### 2-(1-(6-Methoxynaphthalen-2-yl)ethyl)-5-(naphthalen-2-yl)-1,3,4-oxadiazole (**18**)

Following general procedure A (using 2-(6-methoxy-2-naphthyl)propionic acid (naproxen) (46.1 mg, 0.20 mmol, 1.0 equiv), and 2-iodonaphthalene (127 mg, 0.50 mmol, 2.5 equiv)): after FCC (25% EtOAc/Pentane), **18** (52.5 mg, 0.138 mmol, 69%) was afforded as a beige crystalline solid. mp 58–60 °C; ^1^H NMR (400 MHz, CDCl_3_) δ 8.46–8.44 (m, 1H), 8.08 (dd, *J* = 8.6, 1.7, 1H), 7.89 (dd, *J* = 9.1, 2.5, 2H), 7.86–7.81 (m, 1H), 7.78–7.72 (m, 3H), 7.59–7.45 (m, 3H), 7.20–7.11 (m, 2H), 4.61 (q, *J* = 7.2, 1H), 3.91 (s, 3H), 1.94 (d, *J* = 7.2, 3H); ^13^C{^1^H} NMR (101 MHz, CDCl_3_) δ 169.0, 165.3, 157.9, 135.5, 134.6, 133.9, 132.8, 129.3, 129.0, 128.9, 128.8, 127.93, 127.86, 127.6, 127.2, 127.0, 125.92, 125.89, 123.2, 121.3, 119.3, 105.7, 55.3, 37.6, 19.7; IR ν_max_/cm^–1^ 2980, 1606, 1485, 1392, 1267, 1231, 1217, 1174, 1063, 1031, 907, 730; (ESI-TOF) *m*/*z* [M + H]^+^ calcd for C_25_H_21_O_2_N_2_ 381.1598, found 381.1600.

#### 2-(1-(4-Isobutylphenyl)ethyl)-5-(3-methoxyphenyl)-1,3,4-oxadiazole (**19**)

Following general procedure A (using ibuprofen (41.3 mg, 0.20 mmol, 1.0 equiv), and 1-iodo-3-methoxybenzene (60 μL, 0.50 mmol, 2.5 equiv)): after FCC (35% Et_2_O/Pentane), **19** (40.1 mg, 0.120 mmol, 60%) was afforded as a yellow oil. ^1^H NMR (400 MHz, CDCl_3_) δ 7.60–7.53 (m, 2H), 7.37 (t, *J* = 8.0, 1H), 7.29–7.23 (m, 2H), 7.13 (d, *J* = 8.2, 2H), 7.05 (ddd, *J* = 8.3, 2.6, 0.9, 1H), 4.42 (q, *J* = 7.2, 1H), 3.87 (s, 3H), 2.46 (d, *J* = 7.2, 2H), 1.91–1.83 (m, 1H), 1.82 (d, *J* = 7.3, 3H), 0.90 (d, *J* = 6.6, 6H); ^13^C{^1^H} NMR (101 MHz, CDCl_3_) δ 169.0, 164.9, 159.9, 141.0, 137.6, 130.1, 129.6, 127.0, 125.2, 119.3, 117.9, 111.6, 55.5, 45.0, 37.2, 30.2, 22.4, 19.7; IR ν_max_/cm^–1^ 2953, 2868, 1566, 1550, 1466, 1242, 1043, 852, 727, 686; (ESI-TOF) *m*/*z* [M + H]^+^ calcd for C_21_H_25_O_2_N_2_ 337.1911, found 337.1909.

### General Procedure B: One-Pot 1,3,4-Oxadiazole Synthesis-Amination (**20**–**25**)

To a dry Schlenk tube under nitrogen was added carboxylic acid (0.20 mmol, 1.2 equiv) and NIITP (66.5 mg, 0.22 mmol, 1.32 equiv). The Schlenk tube was then evacuated and backfilled with nitrogen (×4), and the anhydrous 1,4-dioxane (0.50 mL, 0.33 M) was added. The Schlenk tube was then sealed and put into an oil bath preheated at 80 °C and stirred for 3 h. After this time the reaction was cooled to rt and *O*-benzoyl hydroxylamine (0.167 mmol, 1.0 equiv), triphenylphosphine (65.6 mg, 0.25 mmol, 1.5 equiv), lithium *tert-*butoxide (32.0 mg, 0.40 mmol, 2.4 equiv), copper(II) acetate (22.7 mg, 0.13 mmol, 0.75 equiv), and anhydrous 1,4-dioxane (0.50 mL, 0.17 M total) were added sequentially. The Schlenk tube was then sealed and put into an oil bath preheated at 40 °C and stirred for 18 h. After this time the reaction was cooled to rt and filtered through a silica plug, washing with EtOAc, and then concentrated in vacuo to afford the crude product. The crude product was purified by FCC, and subsequent PTLC when required, to afford the pure 2-amino-5-substituted 1,3,4-oxadiazole product.

#### 2-(4-Fluorophenyl)-5-(piperidin-1-yl)-1,3,4-oxadiazole (**20**)

Following general procedure B (using 4-fluorobenzoic acid (28.0 mg, 0.20 mmol, 1.2 equiv), and morpholino benzoate (34.6 mg, 0.167 mmol, 1.0 equiv)): after FCC (25% Acetone/Pentane) and PTLC (75% EtOAc/Hexane), **20** (26.7 mg, 0.106 mmol, 64%) was afforded as a white powder. mp 136–138 °C; ^1^H NMR (400 MHz, CDCl_3_) δ 7.94–7.86 (m, 2H), 7.18–7.10 (m, 2H), 3.87–3.81 (m, 4H), 3.61–3.57 (m, 4H); ^19^F NMR (377 MHz, CDCl_3_) δ −108.5; ^13^C{^1^H} NMR (101 MHz, CDCl_3_) δ 164.1 (d, *J* = 251.7), 164.1, 158.8, 128.0 (d, *J* = 8.6), 120.8 (d, *J* = 3.4), 116.2 (d, *J* = 22.3), 65.9, 46.3; IR ν_max_/cm^–1^ 2865, 1618, 1608, 1502, 1274, 1121, 912, 816, 732, 617; (ESI-TOF) *m*/*z* [M + H]^+^ calcd for C_12_H_13_O_2_N_3_F 250.0986, found 250.0987.

#### 4-(5-Morpholino-1,3,4-oxadiazol-2-yl)-*N*,*N*-dipropylbenzenesulfonamide (**21**)

Following general procedure B (using probenecid (57.1 mg, 0.20 mmol, 1.2 equiv), and morpholino benzoate (34.6 mg, 0.167 mmol, 1.0 equiv)): after FCC (30% MeCN/CH_2_Cl_2_), **21** (29.0 mg, 0.073 mmol, 44%) was afforded as a white powder. mp 108–110 °C; ^1^H NMR (400 MHz, CDCl_3_) δ 8.02 (d, *J* = 8.7, 2H), 7.87 (d, *J* = 8.7, 2H), 3.87–3.80 (m, 4H), 3.64–3.57 (m, 4H), 3.14–3.04 (m, 4H), 1.54 (h, *J* = 7.4, 4H), 0.86 (t, *J* = 7.4, 6H); ^13^C{^1^H} NMR (101 MHz, CDCl_3_) δ 164.3, 158.3, 142.0, 127.8, 127.6, 126.1, 65.9, 49.9, 46.2, 21.9, 11.2; IR ν_max_/cm^–1^ 2967, 1613, 1339, 1274, 1156, 1117, 913, 754, 729; (ESI-TOF) *m*/*z* [M + H]^+^ calcd for C_18_H_27_O_4_N_4_S 395.1748, found 395.1745.

#### 4-(5-(1-(6-Methoxynaphthalen-2-yl)ethyl)-1,3,4-oxadiazol-2-yl)morpholine (**22**)

Following general procedure B (using naproxen (41.6 mg, 0.20 mmol, 1.2 equiv), and morpholino benzoate (34.6 mg, 0.167 mmol, 1.0 equiv)): after FCC (40% Acetone/Pentane) and PTLC (70% EtOAc/CHCl_3_), **22** (27.9 mg, 0.082 mmol, 49%) was afforded as a colorless oil. ^1^H NMR (400 MHz, CDCl_3_) δ 7.70 (dd, *J* = 8.7, 3.1, 2H), 7.64 (s, 1H), 7.37 (dd, *J* = 8.5, 1.8, 1H), 7.15 (dd, *J* = 8.9, 2.5, 1H), 7.11 (d, *J* = 2.4, 1H), 4.32 (q, *J* = 7.2, 1H), 3.91 (s, 3H), 3.77–3.68 (m, 4H), 3.46–3.36 (m, 4H), 1.77 (d, *J* = 7.2, 3H); ^13^C{^1^H} NMR (101 MHz, CDCl_3_) δ 164.5, 163.5, 157.8, 135.8, 133.8, 129.3, 128.9, 127.4, 125.9, 125.7, 119.2, 105.7, 65.9, 55.3, 46.1, 37.5, 19.4; IR ν_max_/cm^–1^ 2974, 2858, 1620, 1568, 1452, 1266, 1216, 1118, 1029, 912, 853; (ESI-TOF) *m*/*z* [M + H]^+^ calcd for C_19_H_22_O_3_N_3_ 340.1656, found 340.1653

#### 4-(5-(1-(4-Isobutylphenyl)ethyl)-1,3,4-oxadiazol-2-yl)morpholine (**23**)

Following general procedure B (using ibuprofen (41.3 mg, 0.20 mmol, 1.2 equiv), and morpholino benzoate (34.6 mg, 0.167 mmol, 1.0 equiv)): after FCC (20% MeCN/CH_2_Cl_2_ → 10% Methanol/CH_2_Cl_2_) and PTLC (33% Acetone/Toluene), **23** (24.4 mg, 0.077 mmol, 46%) was afforded as a yellow oil. ^1^H NMR (400 MHz, CDCl_3_) δ 7.18 (d, *J* = 8.1, 2H), 7.10 (d, *J* = 8.1, 2H), 4.17 (q, *J* = 7.2, 1H), 3.79–3.71 (m, 4H), 3.46–3.39 (m, 4H), 2.44 (d, *J* = 7.2, 2H), 1.90–1.78 (m, 1H), 1.68 (d, *J* = 7.3, 3H), 0.89 (d, *J* = 6.6, 6H); ^13^C{^1^H} NMR (101 MHz, CDCl_3_) δ 164.4, 163.5, 140.8, 137.9, 129.5, 126.9, 65.9, 46.1, 45.0, 37.1, 30.2, 22.4, 19.5; IR ν_max_/cm^–1^ 2957, 2865, 1620, 1569, 1453, 1274, 1118, 913; (ESI-TOF) *m*/*z* [M + H]^+^ calcd for C_18_H_26_O_2_N_3_ 316.2020, found 316.2018.

#### 4-(5-(5-(2,5-Dimethylphenoxy)-2-methylpentan-2-yl)-1,3,4-oxadiazol-2-yl)morpholine (**24**)

Following general procedure B (using gemfibrozil (50.1 mg, 0.20 mmol, 1.2 equiv), and morpholino benzoate (34.6 mg, 0.167 mmol, 1.0 equiv)): after FCC (25% Acetone/Pentane) and FCC (75% EtOAc/Pentane), **24** (31.8 mg, 0.088 mmol, 53%) was afforded as a colorless oil. ^1^H NMR (400 MHz, CDCl_3_) δ 6.99 (d, *J* = 7.5, 1H), 6.65 (d, *J* = 7.5, 1H), 6.58 (s, 1H), 3.90 (t, *J* = 5.9, 2H), 3.82–3.74 (m, 4H), 3.49–3.40 (m, 4H), 2.30 (s, 3H), 2.16 (s, 3H), 1.88–1.80 (m, 2H), 1.80–1.70 (m, 2H), 1.39 (s, 6H); ^13^C{^1^H} NMR (101 MHz, CDCl_3_) δ 166.8, 164.3, 156.9, 136.5, 130.3, 123.4, 120.8, 111.9, 67.6, 65.9, 46.2, 37.6, 35.5, 25.9, 24.9, 21.4, 15.8; IR ν_max_/cm^–1^ 2970, 2921, 1615, 1565, 1509, 1453, 1263, 1157, 1119, 1046, 912; (ESI-TOF) *m*/*z* [M + H]^+^ calcd for C_20_H_30_O_3_N_3_ 360.2282, found 360.2278.

#### *N*-(3-(10,11-Dihydro-5*H*-dibenzo[*a*,*d*][7]annulen-5-ylidene)propyl)-5-(5-(2,5-dimethylphenoxy)-2-methylpentan-2-yl)-*N*-methyl-1,3,4-oxadiazol-2-amine (**25**)

Following general procedure B (using gemfibrozil (50.1 mg, 0.20 mmol, 1.2 equiv), and *O*-benzoyl-*N*-(3-(10,11-dihydro-5*H*-dibenzo[*a*,*d*][7]annulen-5-ylidene)propyl)-*N*-methylhydroxylamine^18^ (64.0 mg, 0.167 mmol, 1.0 equiv)): after FCC (30% → 60% EtOAc/Pentane), **25** (62.3 mg, 0.117 mmol, 70%) was afforded as a yellow oil. ^1^H NMR (400 MHz, CDCl_3_) δ 7.27–7.06 (m, 7H), 7.04–6.96 (m, 2H), 6.66 (d, *J* = 7.5, 1H), 6.58 (s, 1H), 5.83 (t, *J* = 7.5, 1H), 3.83 (t, *J* = 5.9, 2H), 3.55–3.18 (m, 4H), 3.00–2.86 (m, 1H), 2.90 (s, 3H), 2.82–2.68 (m, 1H), 2.53–2.35 (m, 2H), 2.31 (s, 3H), 2.17 (s, 3H), 1.83–1.75 (m, 2H), 1.74–1.65 (m, 2H), 1.31 (s, 6H); ^13^C{^1^H} NMR (151 MHz, CDCl_3_) δ 165.9, 164.4, 157.0, 145.4, 140.9, 139.7, 139.4, 137.1, 136.5, 130.3, 130.1, 128.5, 128.2, 128.0, 127.7, 127.3, 127.1, 126.1, 125.8, 123.5, 120.7, 111.9, 67.7, 50.6, 37.6, 35.7, 35.4, 33.7, 32.0, 27.6, 25.9, 24.9, 21.5, 15.8; IR ν_max_/cm^–1^ 2973, 1633, 1572, 1508, 1265, 1128, 1042, 909, 730; (ESI-TOF) *m*/*z* [M + H]^+^ calcd for C_35_H_42_O_2_N_3_ 536.3272, found 536.3264.

## Supporting Information Available

General synthetic procedures, additional experimental details, and NMR spectra (PDF)
