# Supplementary material for: A One-Pot Synthesis-Functionalization Strategy for Streamlined Access to 2,5-Disubstituted 1,3,4-Oxadiazoles from Carboxylic Acids
Source: J Org Chem. 2022 Sep 2;87(18):12498–505. doi: 10.1021/acs.joc.2c01669 (PMC9486941; doi:10.1021/acs.joc.2c01669)
Supplement: Supplementary file 1 — jo2c01669_si_001.pdf [file jo2c01669_si_001.pdf]

Supporting information

For

A One-Pot Synthesis-Functionalization Strategy for Streamlined Access to 2,5-Disubstituted 1,3,4-Oxadiazoles from Carboxylic Acids

Daniel Matheau-Raven and Darren J. Dixon*

Department of Chemistry, Chemistry Research Laboratory, University of Oxford, 12 Mansfield Road, Oxford, OX1 3TA, UK.

Table of Contents

General information....3

General procedures....5

General Procedure A: One-pot 1,3,4-oxadiazole synthesis-arylation....5

General Procedure B: One-pot 1,3,4-oxadiazole synthesis-amination....6

Synthesis and characterization of starting materials....7

NMR data....8

References....37

General information

General Techniques: Reactions were carried out under a nitrogen atmosphere unless stated otherwise. Glassware was oven-dried and cooled under vacuum then purged with nitrogen before use. Room temperature refers to 22 ± 2 °C. Reactions carried out at high temperatures were heated using an oil bath. Reaction temperatures refer to external temperatures of an oil bath.

Nomenclature and Numbering: Compounds are named following IUPAC nomenclature as generated by ChemDraw.

Solvents and Reagents: Anhydrous solvents were either obtained from Sure/Seal™ bottles purchased from Sigma-Aldrich, an MBRAUN-SPS solvent purification system in which solvent is passed through an activated alumina column under nitrogen. Reagents were used as obtained without further purification unless stated otherwise.

Chromatography: Thin layer chromatography (TLC) was carried out using Merck aluminium backed DC60 F254 plates (particle size 0.2 mm). TLC sheets were visualised by UV light, then developed by staining with potassium permanganate. Purification by flash column chromatography (FCC) was carried out using Merck silicagel 60 F254 (particle size 43–60 μm).

Characterisation: Proton (^{1}H), Fluorine (^{19}F), and carbon (^{13}C) spectra were recorded on Bruker AVANCE *NEO600,* Bruker AVG400, Bruker AVH400, Bruker AVF400, Bruker AVB500, Bruker AVC500, and Bruker DPX200 NMR spectrometers. Spectra are referenced to the residual solvent peak. Chemical shifts (δ) are given in parts per million (ppm, ± 0.01) and coupling constants (*J*) are given in Hertz (Hz, ± 0.1 as measured on Mestrenova, without rounding). The following convention is used to report chemical shifts: δ (multiplicity, coupling constant(s), number of protons), with chemical shifts reported in descending order. Peak multiplicities are described as singlet (s), doublet (d), triplet (t), quartet (q), pentet (p), heptet (h), nonuplet (n), a combination e.g. doublet of doublets (dd), or as a multiplet (m) over a peak range. Infrared spectra were recorded using a Bruker Tensor 27 FT-IR spectrometer. Selected diagnostic absorption maxima (ν_{max}) are reported in wavenumbers (cm^{-1}). High resolution mass spectra were recorded by Chemistry Research Laboratory staff using a Bruker Daltronics MicroTOF spectrometer (ESI). Mass to charge ratios (m/z) are reported in Daltons. Melting points were recorded using a Leica Galen III hot-stage microscope apparatus and are reported uncorrected in degrees Celsius (°C).

Starting materials: Carboxylic acids, and aryl iodides were obtained from commercial chemical suppliers and used as received. Copper(I) iodide (43153, Puratronic®, 99.998% metal basis), and copper(II) acetate (44355, 99.999% metal basis) were purchased from Alfa Aesar and used as

received. (*N*-isocyanimino) triphenylphosphorane (NIITP) was synthesised according to Bio's method.[1] Morpholino benzoate was synthesised according to a literature procedure.[2]

General procedures

General Procedure A: One-pot 1,3,4-oxadiazole synthesis-arylation

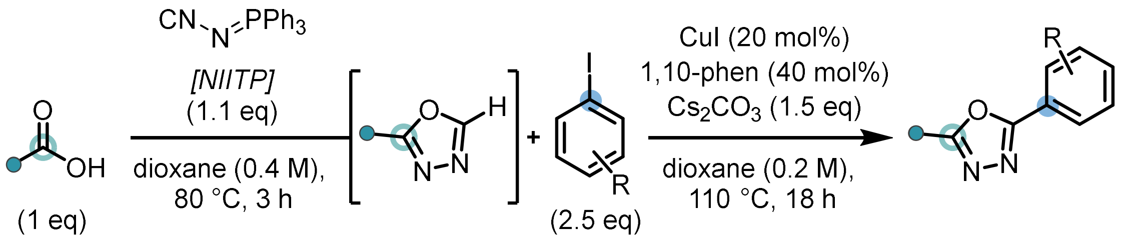

To a dry Schlenk tube under nitrogen was added carboxylic acid (0.20 mmol, 1.0 eq), and NIITP (66.5 mg, 0.22 mmol, 1.1 eq). The Schlenk tube was then evacuated and backfilled with nitrogen (x4) and then anhydrous 1,4-dioxane (0.50 mL, 0.40 M) was added. The Schlenk tube was then sealed and put into an oil bath preheated at 80 °C and stirred for 3 h. After this time the reaction was cooled to rt and aryl iodide (0.50 mmol, 2.5 eq), 1,10-phenanthroline (14.4 mg, 0.08 mmol, 40 mol%), cesium carbonate (97.7 mg, 0.30 mmol, 1.5 eq), copper(I) iodide (7.6 mg, 0.04 mmol, 20 mol%), and anhydrous 1,4-dioxane (0.50 mL, 0.20 M total) were added sequentially. The Schlenk tube was then sealed and put into an oil bath preheated at 110 °C and stirred for 18 h. After this time the reaction was cooled to rt and filtered through a silica plug, washing with EtOAc, and then concentrated *in vacuo* to afford the crude product. The crude product was purified by flash column chromatography (FCC), and subsequent preparative thin-layer chromatography (PTLC) when required, to afford the pure 2,5-disubstituted 1,3,4-oxadiazole product.

General Procedure B: One-pot 1,3,4-oxadiazole synthesis-amination

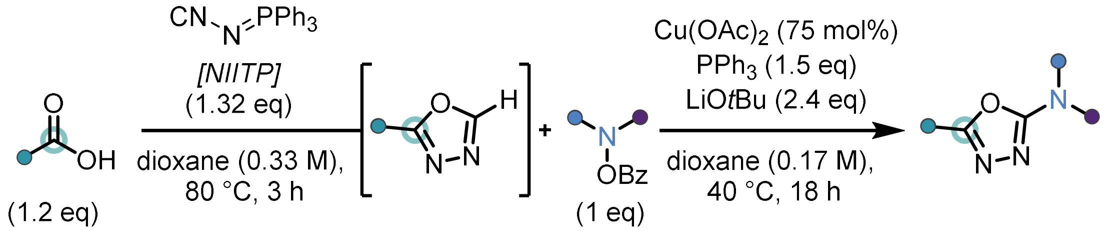

To a dry Schlenk tube under nitrogen was added carboxylic acid (0.20 mmol, 1.2 eq), and NIITP (66.5 mg, 0.22 mmol, 1.32 eq). The Schlenk tube was then evacuated and backfilled with nitrogen (x4) and the anhydrous 1,4-dioxane (0.50 mL, 0.33 M) added. The Schlenk tube was then sealed and put into an oil bath preheated at 80 °C and stirred for 3 h. After this time the reaction was cooled to rt and *O*-benzoyl hydroxylamine (0.167 mmol, 1.0 eq), triphenylphosphine (65.6 mg, 0.25 mmol, 1.5 eq), lithium *tert*-butoxide (32.0 mg, 0.40 mmol, 2.4 eq), copper(II) acetate (22.7 mg, 0.13 mmol, 0.75 eq), and anhydrous 1,4-dioxane (0.50 mL, 0.17 M total) were added sequentially. The Schlenk tube was then sealed and put into an oil bath preheated at 40 °C and stirred for 18 h. After this time the reaction was cooled to rt and filtered through a silica plug, washing with EtOAc, and then concentrated *in vacuo* to afford the crude product. The crude product was purified by FCC, and subsequent PTLC when required, to afford the pure 2-amino-5-substituted 1,3,4-oxadiazole product.

Synthesis and characterization of starting materials

O-benzoyl-N-(3-(10,11-dihydro-5H-dibenzo[a,d][7]annulen-5-ylidene)propyl)-N-methylhydroxylamine – S1

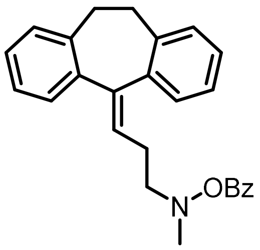

To an oven-dried round-bottomed flask was added nortriptyline hydrochloride (1.00 g, 3.34 mmol, 1.1 eq), benzoyl peroxide (75% with 25% H_2O, 970 mg, 3.0 mmol, 1.0 eq), dipotassium phosphate (1.59 g, 9.1 mmol, 3.0 eq), and anhydrous DMF (15 mL, 0.22 M). The reaction was allowed to stir at rt for 19.5 h, and then was diluted with water (150 mL) and extracted with EtOAc (3 x 50 mL). The combined organics were washed with water (2 x 50 mL), and brine (50 mL), then dried ($MgSO_4$), filtered, and concentrated *in vacuo* to afford the crude product. The crude was purified by FCC (pentane → 30% Et_2O/pentane), and subsequently triturated with pentane and filtered to afford the *title compound* (730 mg, 63%) as a white powder. Data was consistent with that found in the literature.[3]

^{1}H NMR (400 MHz, $CDCl_3$) δ 7.94 (d, *J* = 7.2, 2H), 7.59 (t, *J* = 7.4, 1H), 7.43 (t, *J* = 7.8, 2H), 7.31 (dd, *J* = 7.0, 2.1, 1H), 7.22 – 7.09 (m, 6H), 7.06 (dd, *J* = 6.9, 1.8, 1H), 5.98 (t, *J* = 7.5, 1H), 3.47 – 3.23 (m, 2H), 3.22 – 3.04 (m, 2H), 3.04 – 2.94 (m, 1H), 2.91 (s, 3H), 2.76 (d, *J* = 13.4, 1H), 2.54 (q, *J* = 7.0, 2H).

^{13}C{^{1}H} NMR (101 MHz, $CDCl_3$) δ 165.1, 144.4, 141.0, 139.9, 139.3, 137.1, 133.0, 130.0, 129.4, 129.3, 128.6, 128.4, 128.1, 128.0, 127.4, 127.1, 126.0, 125.8, 60.8, 46.9, 33.8, 32.0, 27.4.

NMR data

O-benzoyl-N-(3-(10,11-dihydro-5H-dibenzo[a,d][7]annulen-5-ylidene)propyl)-N-methylhydroxylamine – S1

Compound **S1** – ^{1}H, $CDCl_3$, 400 MHz

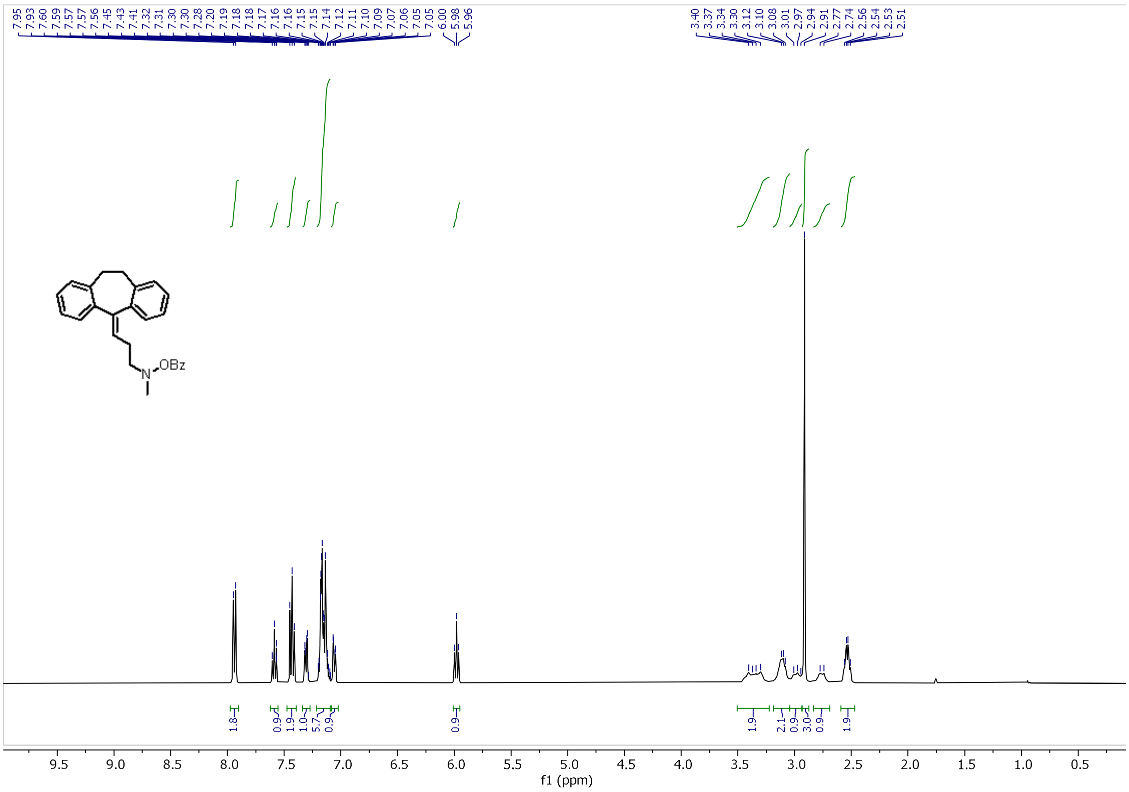

Compound **S1** – $^{13}C\{^{1}H\}$, $CDCl_3$, 101 MHz

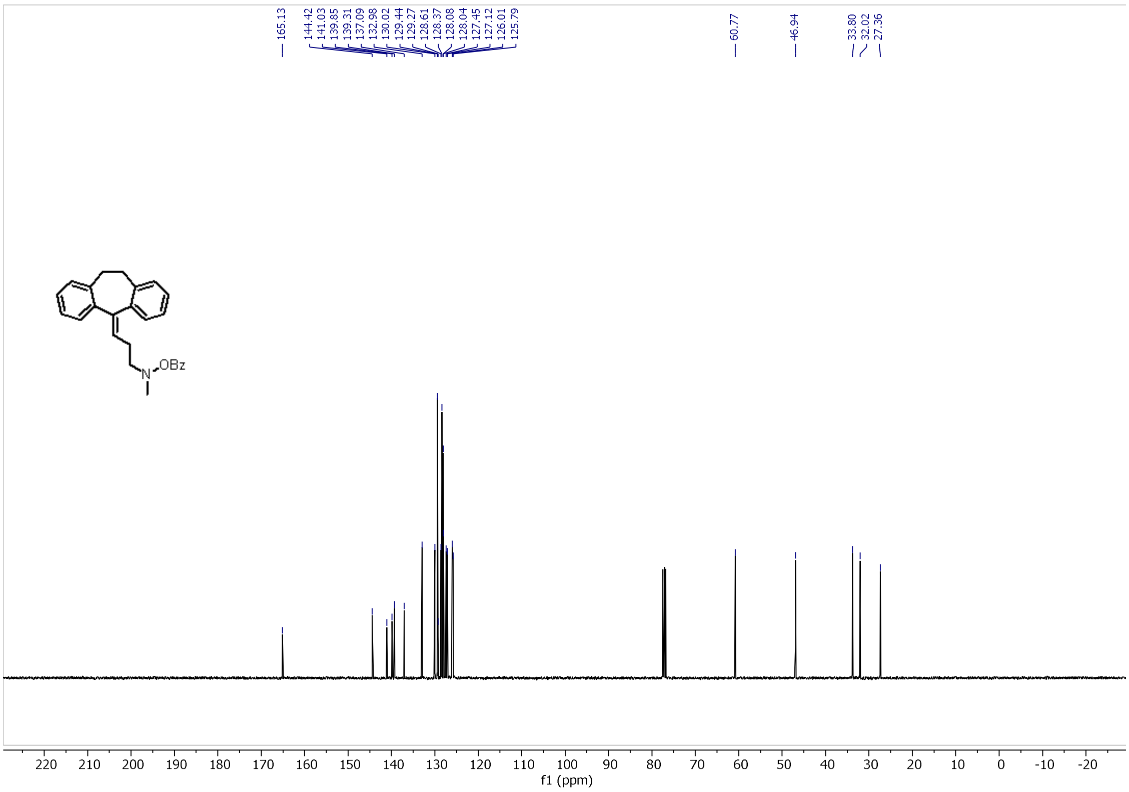

2-(4-fluorophenyl)-5-phenyl-1,3,4-oxadiazole – 2

Compound **2** – ^{1}H, $CDCl_3$, 400 MHz

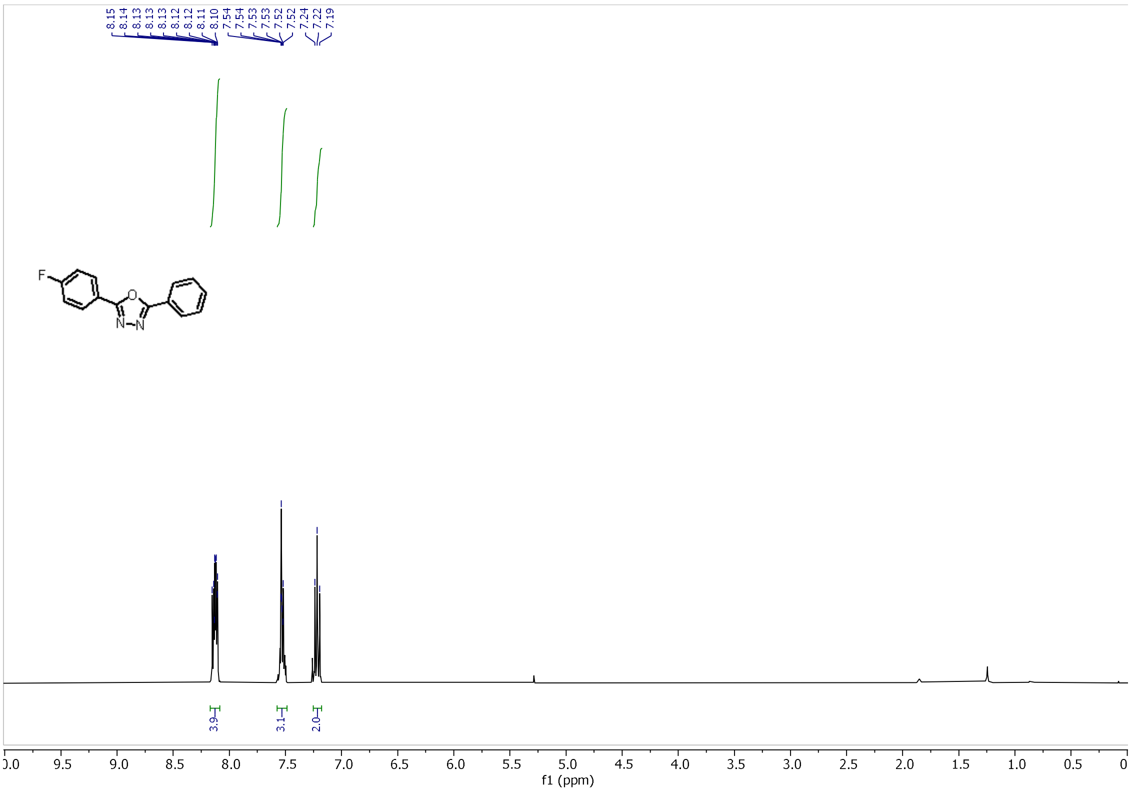

Compound **2** – ^{19}F, $CDCl_3$, 376 MHz

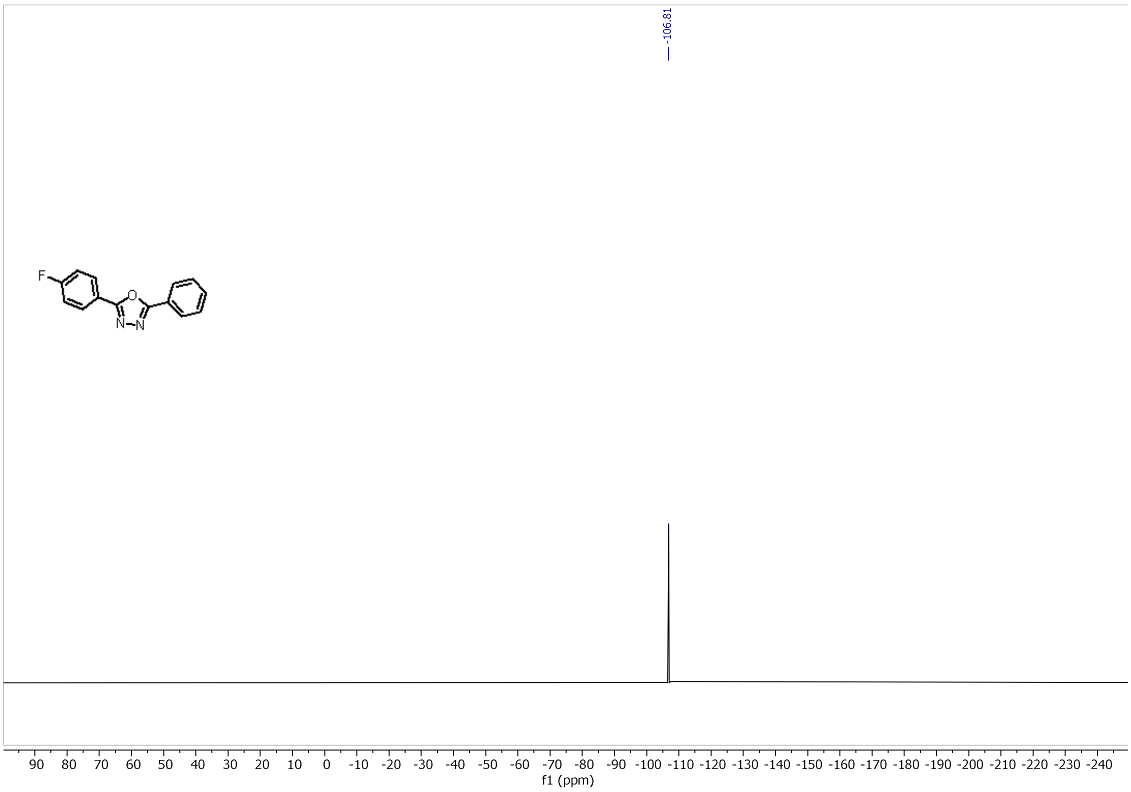

Compound **2** – $^{13}C\{^1H\}$, $CDCl_3$, 101 MHz

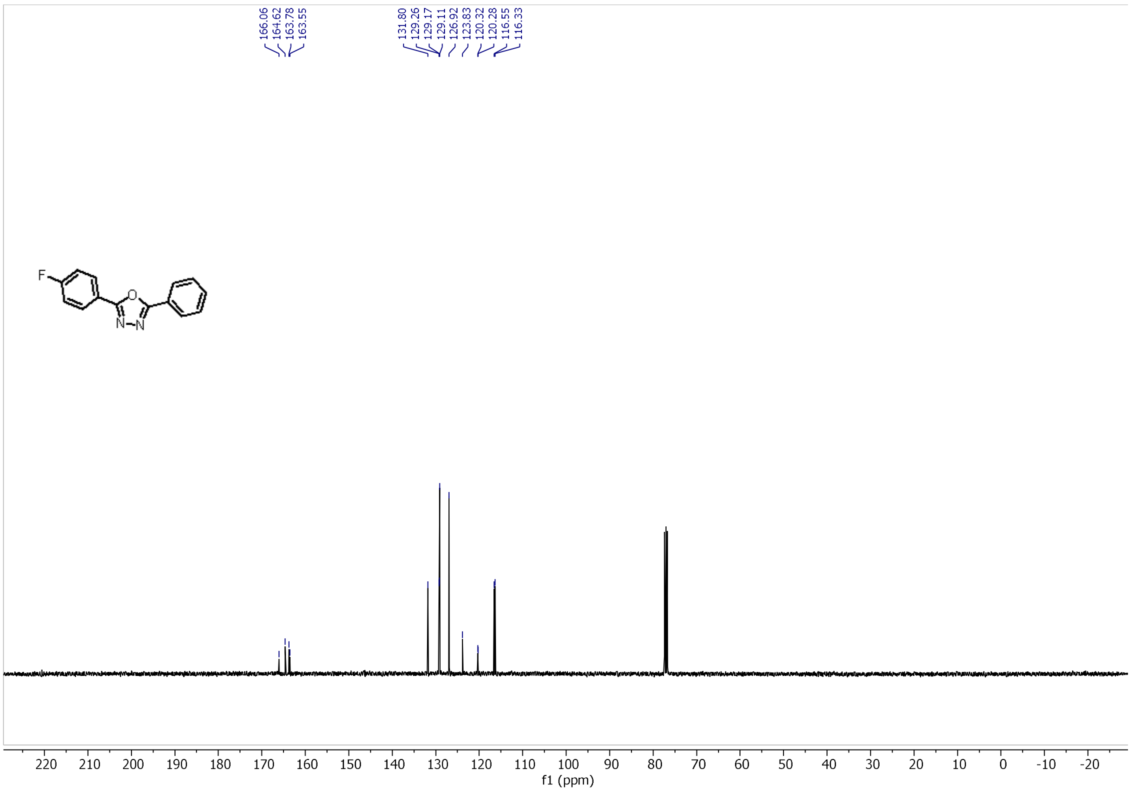

2-(4-(tert-butyl)phenyl)-5-(3-chlorophenyl)-1,3,4-oxadiazole – 3

Compound **3** – ^{1}H, $CDCl_3$, 400 MHz

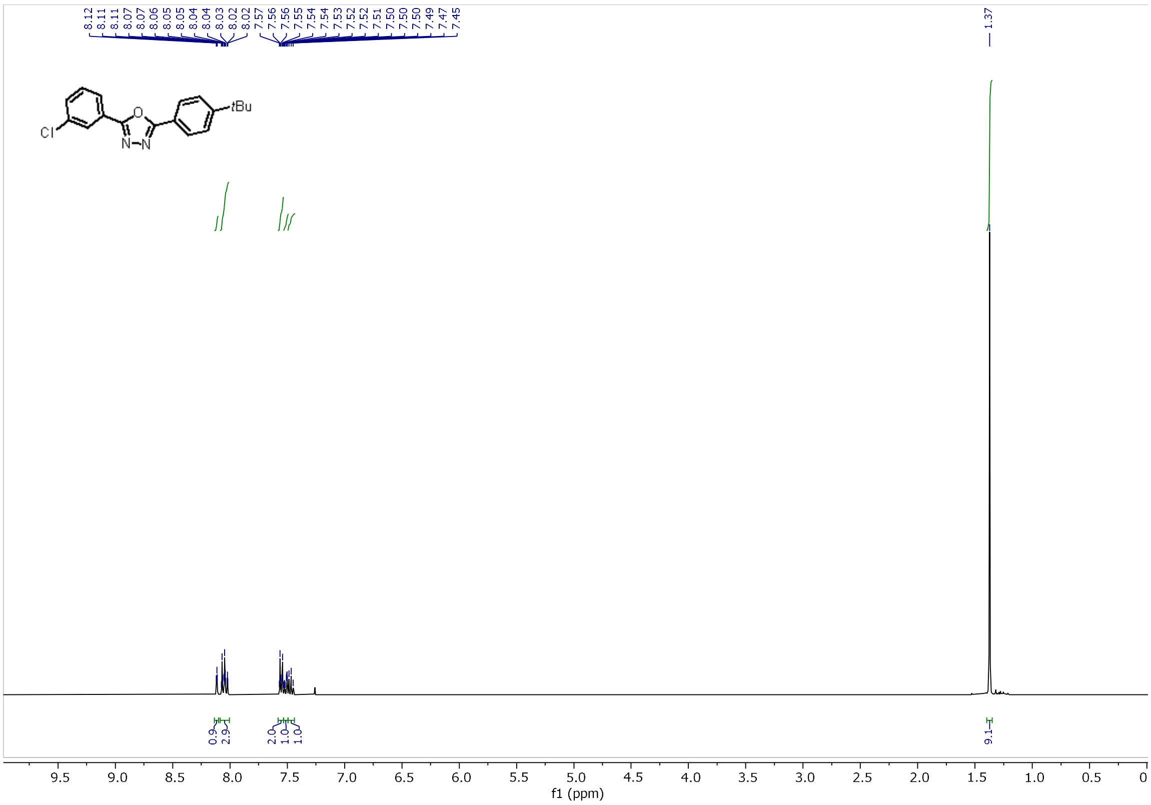

Compound **3** – $^{13}C\{^{1}H\}$, $CDCl_3$, 101 MHz

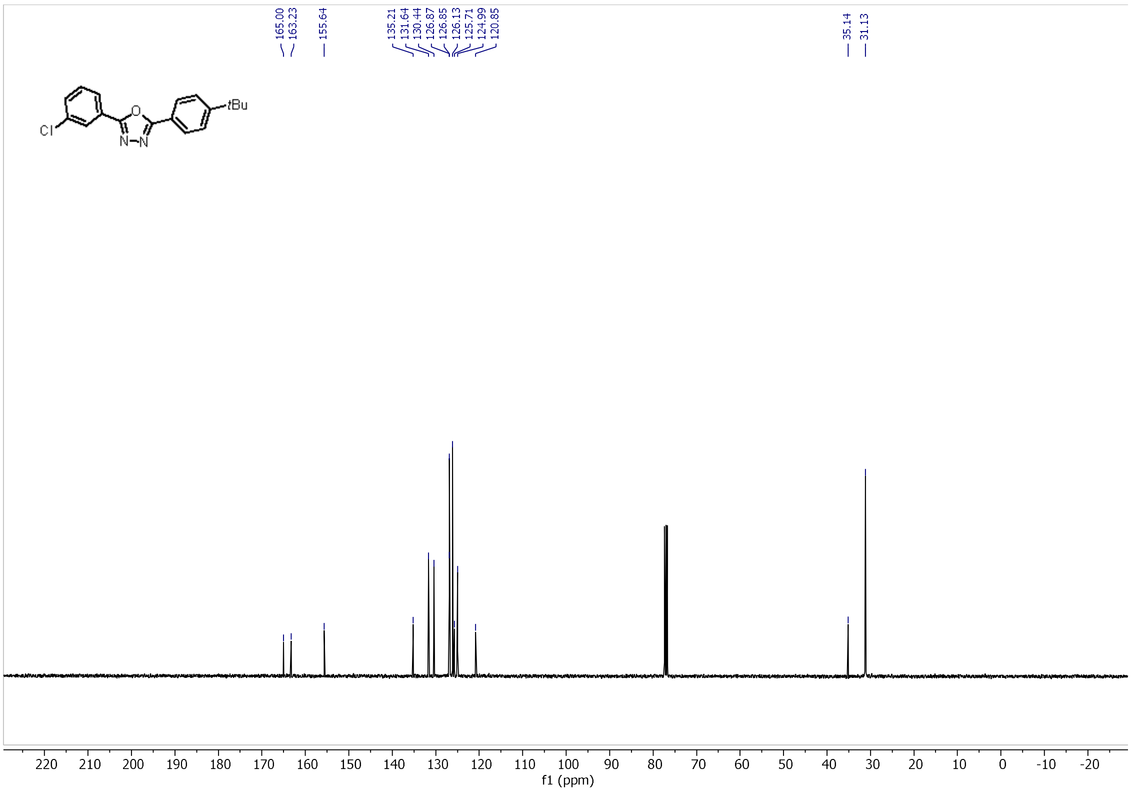

2-(4-bromophenyl)-5-(4-methoxyphenyl)-1,3,4-oxadiazole – 4

Compound **4** – ^{1}H, $CDCl_3$, 400 MHz

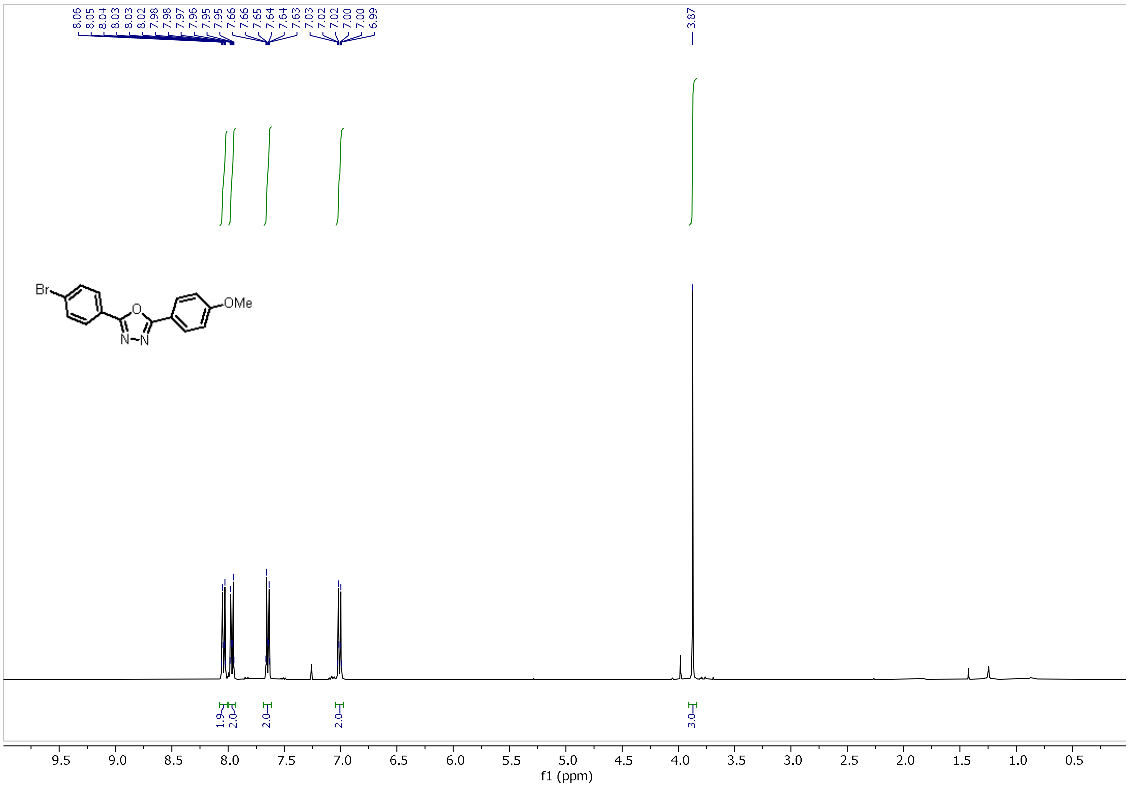

Compound **4** – ^{13}C{^{1}H}, $CDCl_3$, 101 MHz

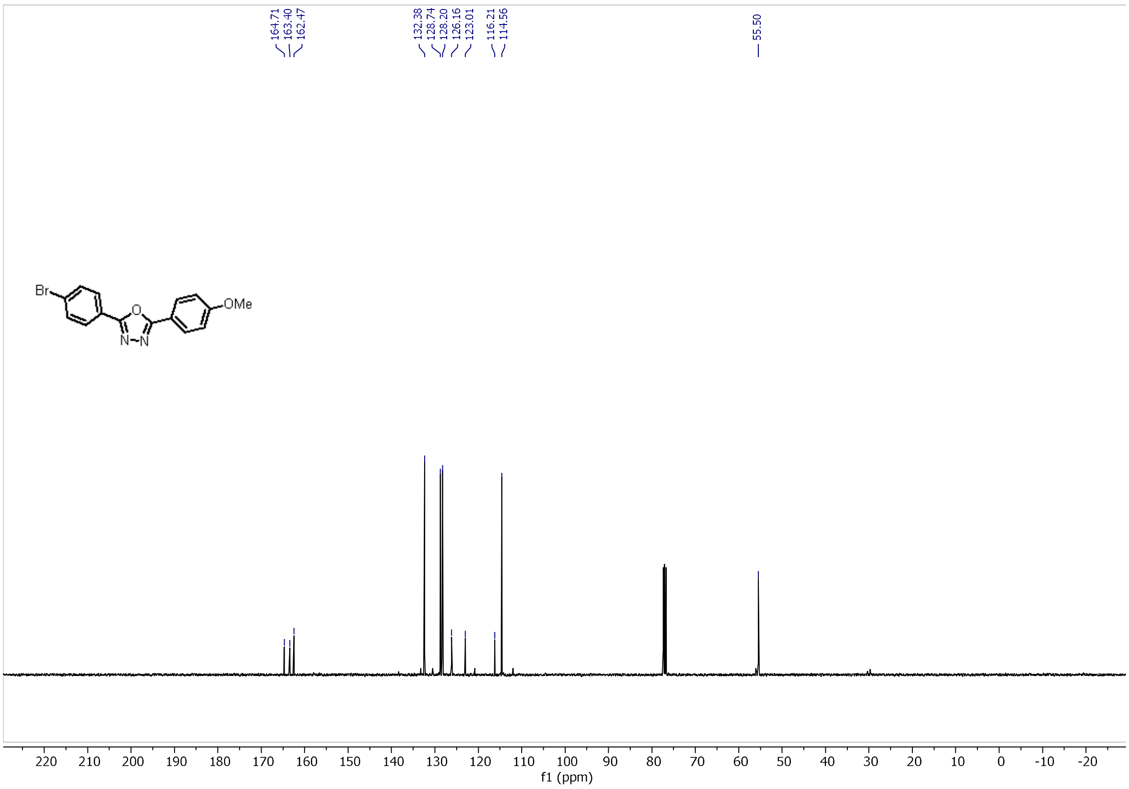

2-(4-fluorophenyl)-5-(p-tolyl)-1,3,4-oxadiazole – 5

Compound **5** – ^{1}H, CDCl$_3$, 400 MHz

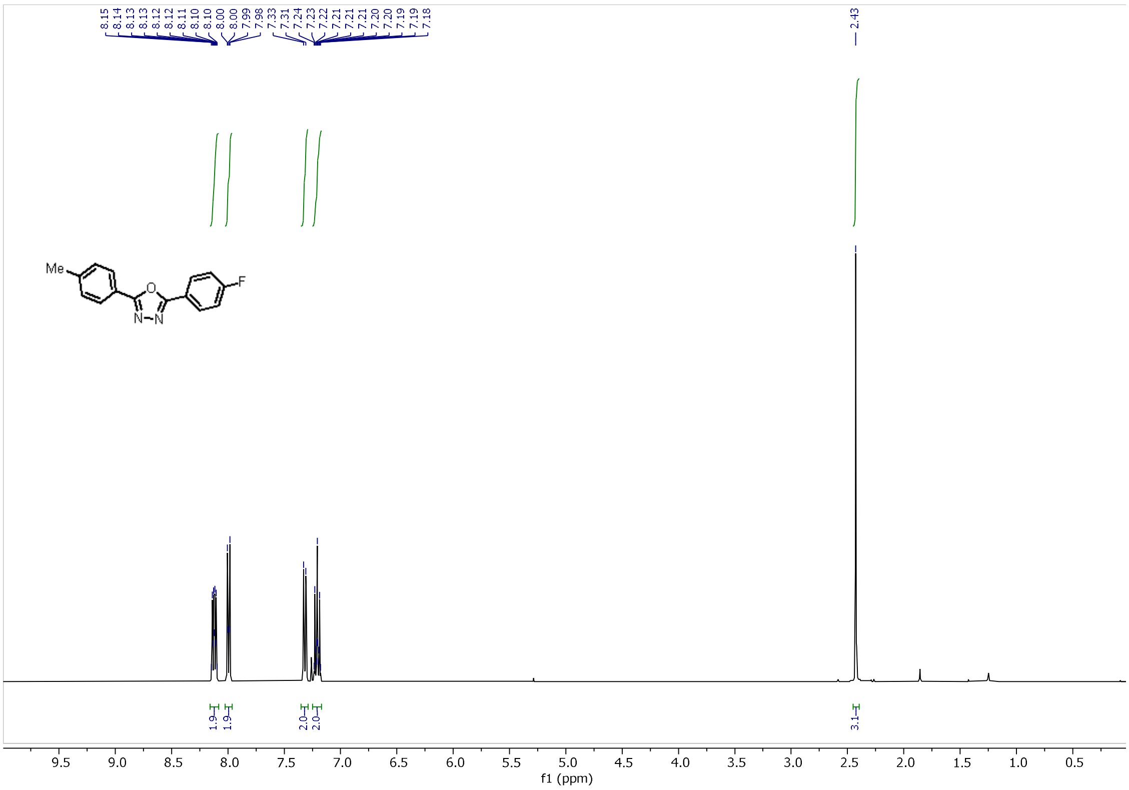

Compound **5** – ^{19}F, CDCl$_3$, 376 MHz

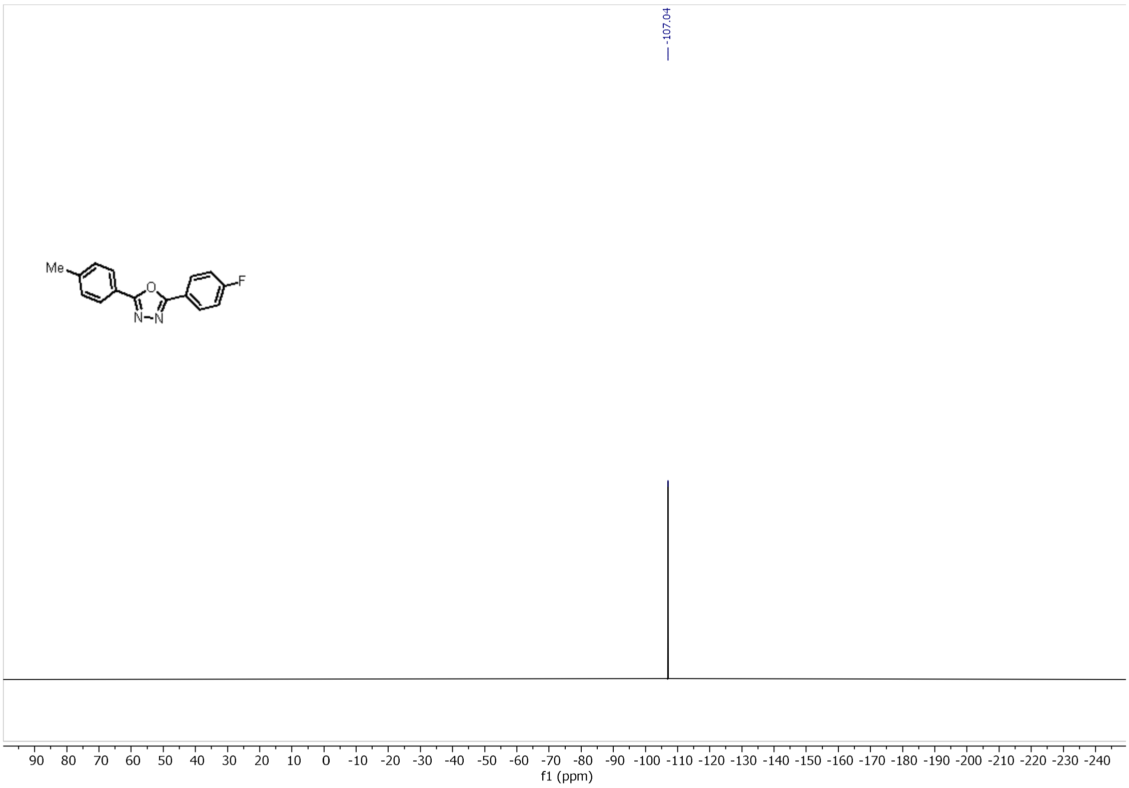

Compound **5** – $^{13}C\{^{1}H\}$, $CDCl_3$, 101 MHz

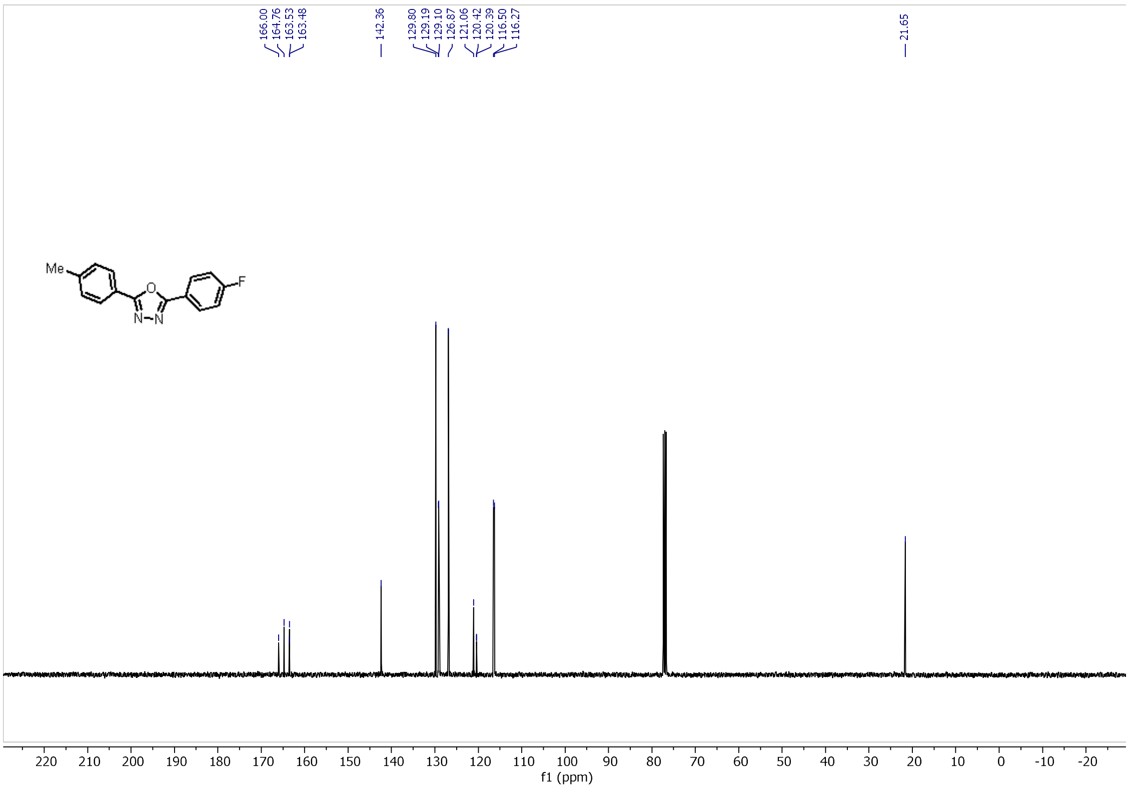

2-(4-chlorophenyl)-5-(m-tolyl)-1,3,4-oxadiazole – 6

Compound **6** – ^{1}H, $CDCl_3$, 400 MHz

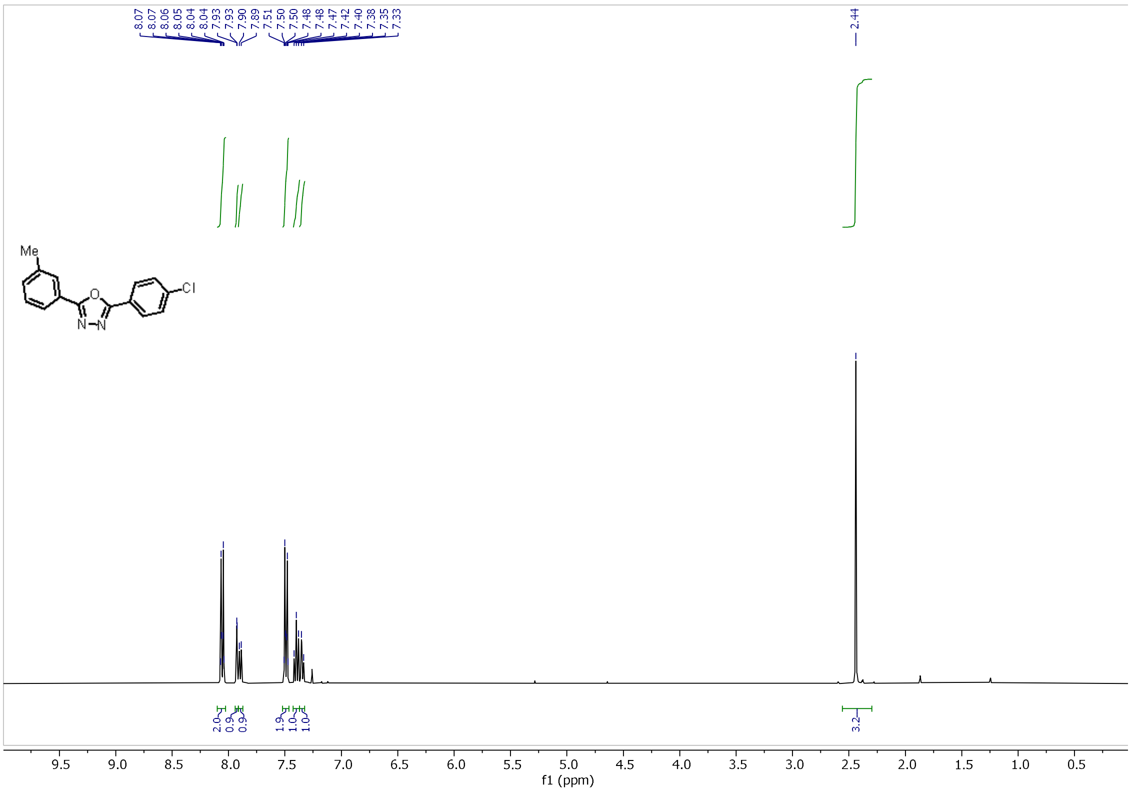

Compound **6** – $^{13}C\{^1H\}$, $CDCl_3$, 101 MHz

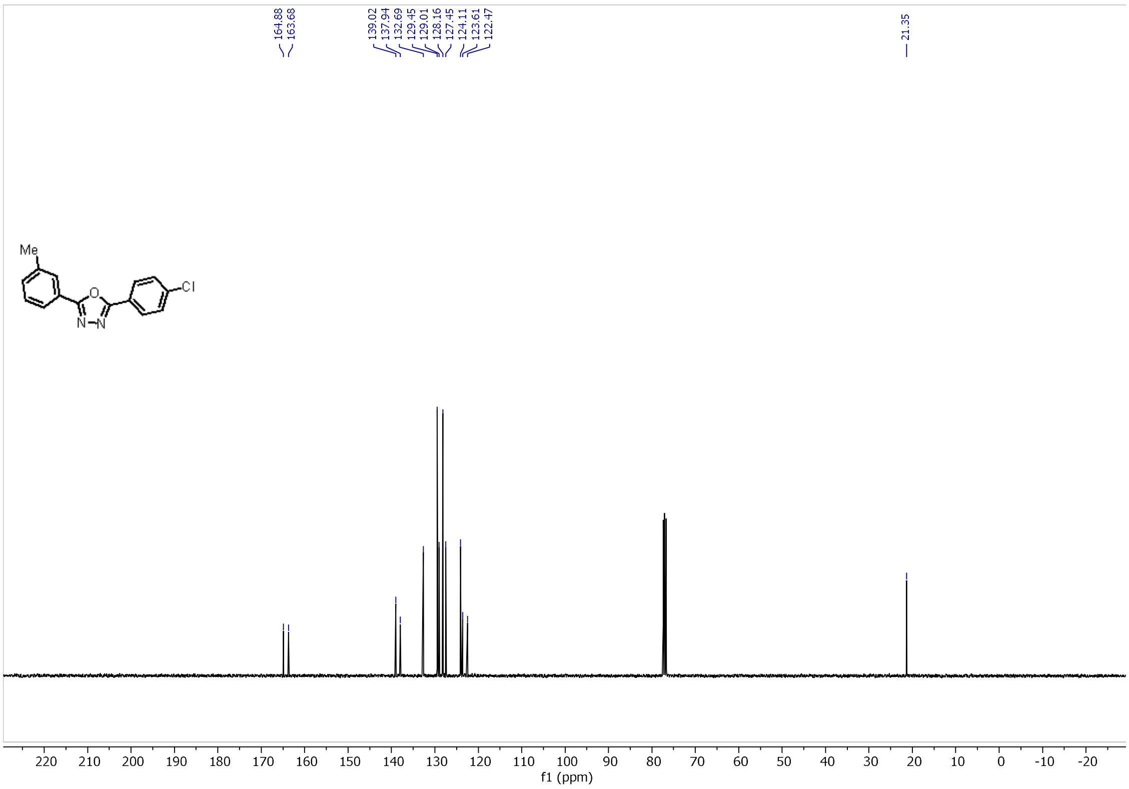

2-(4-bromophenyl)-5-(o-tolyl)-1,3,4-oxadiazole – 7

Compound **7** – ^1H, $CDCl_3$, 400 MHz

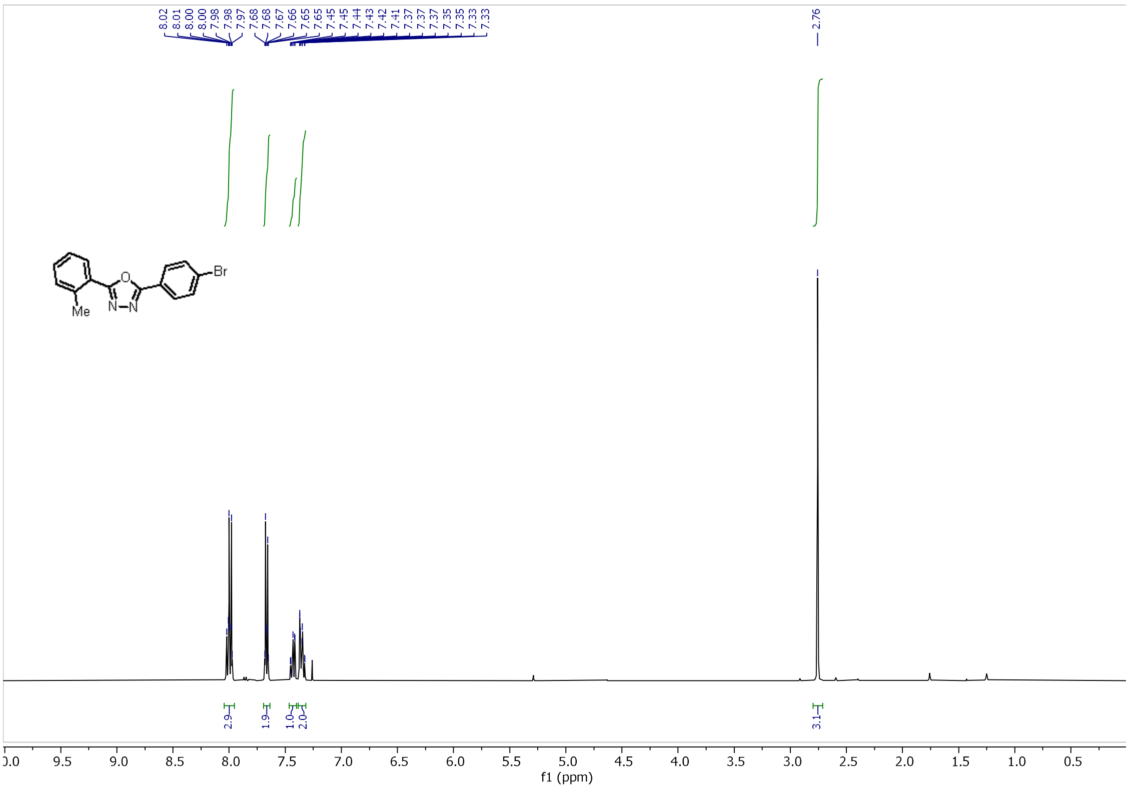

Compound **7** – $^{13}C\{^{1}H\}$, $CDCl_3$, 101 MHz

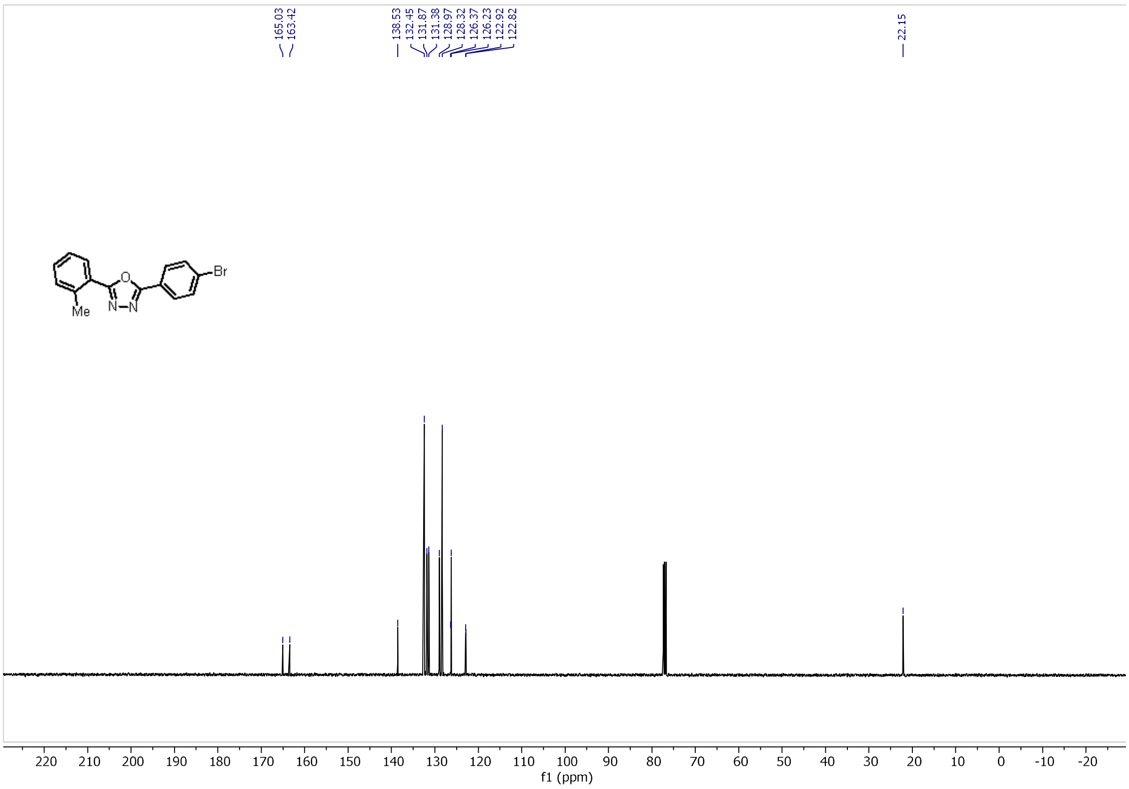

2-(4-methoxyphenyl)-5-(o-tolyl)-1,3,4-oxadiazole – 8

Compound **8** – ^{1}H, $CDCl_3$, 400 MHz

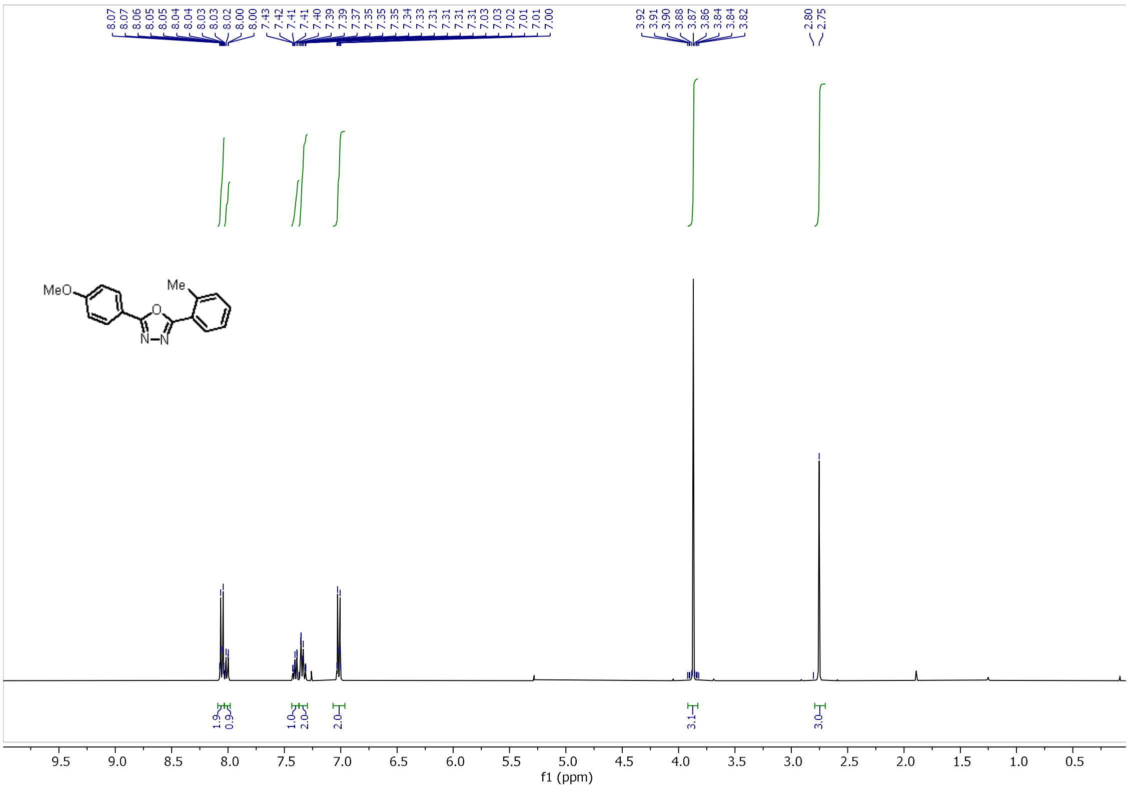

Compound **8** – $^{13}C\{^1H\}$, $CDCl_3$, 101 MHz

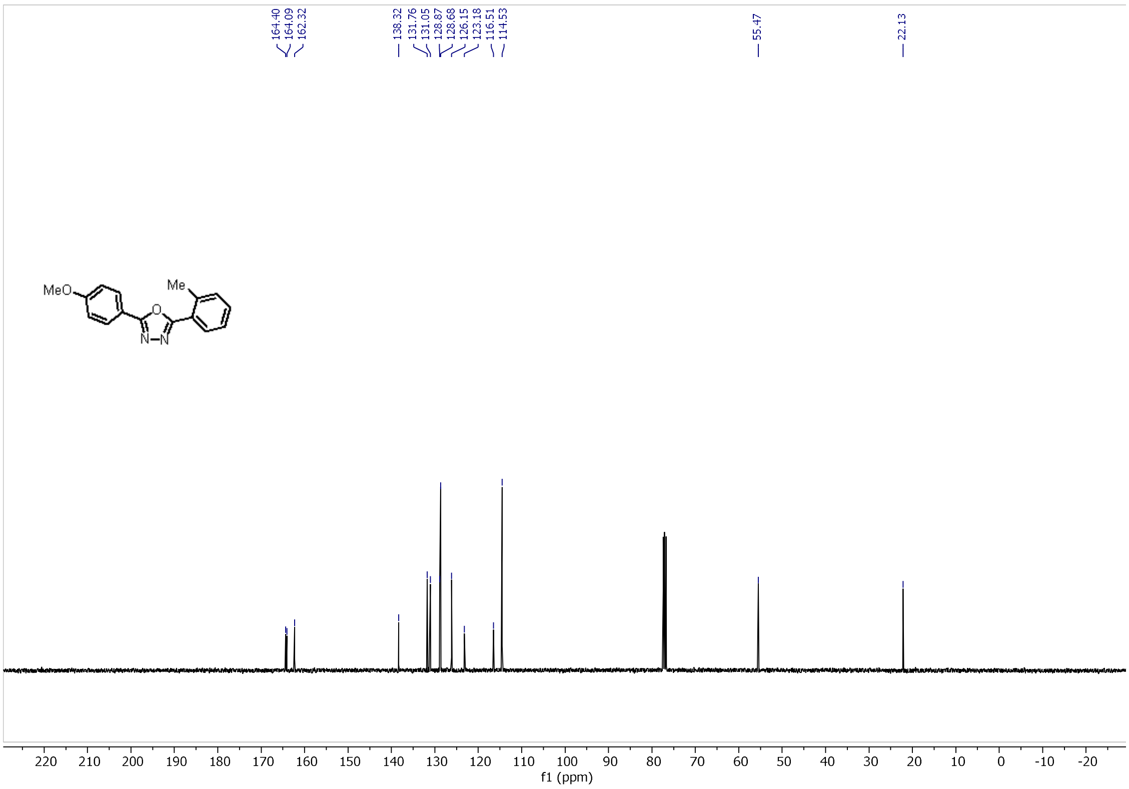

2,5-bis(4-(trifluoromethyl)phenyl)-1,3,4-oxadiazole – 9

Compound **S9** – ^1H, $CDCl_3$, 400 MHz

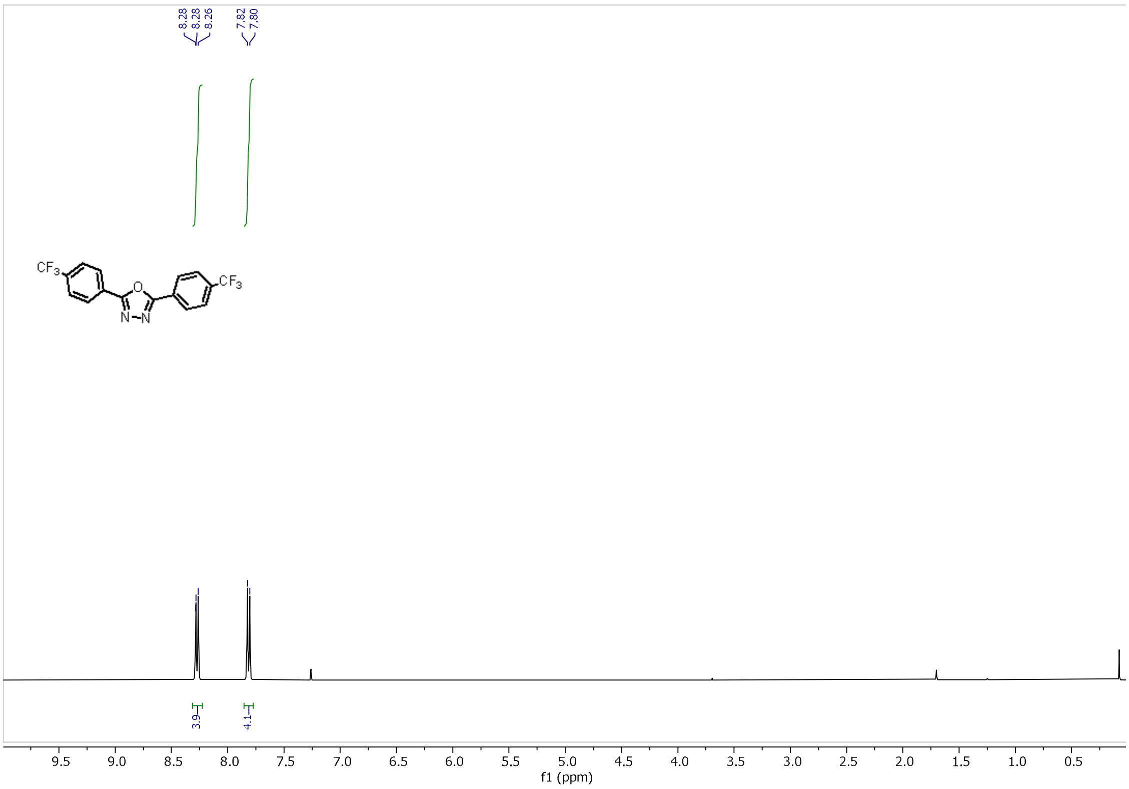

Compound **9** – ^{19}F, $CDCl_3$, 376 MHz

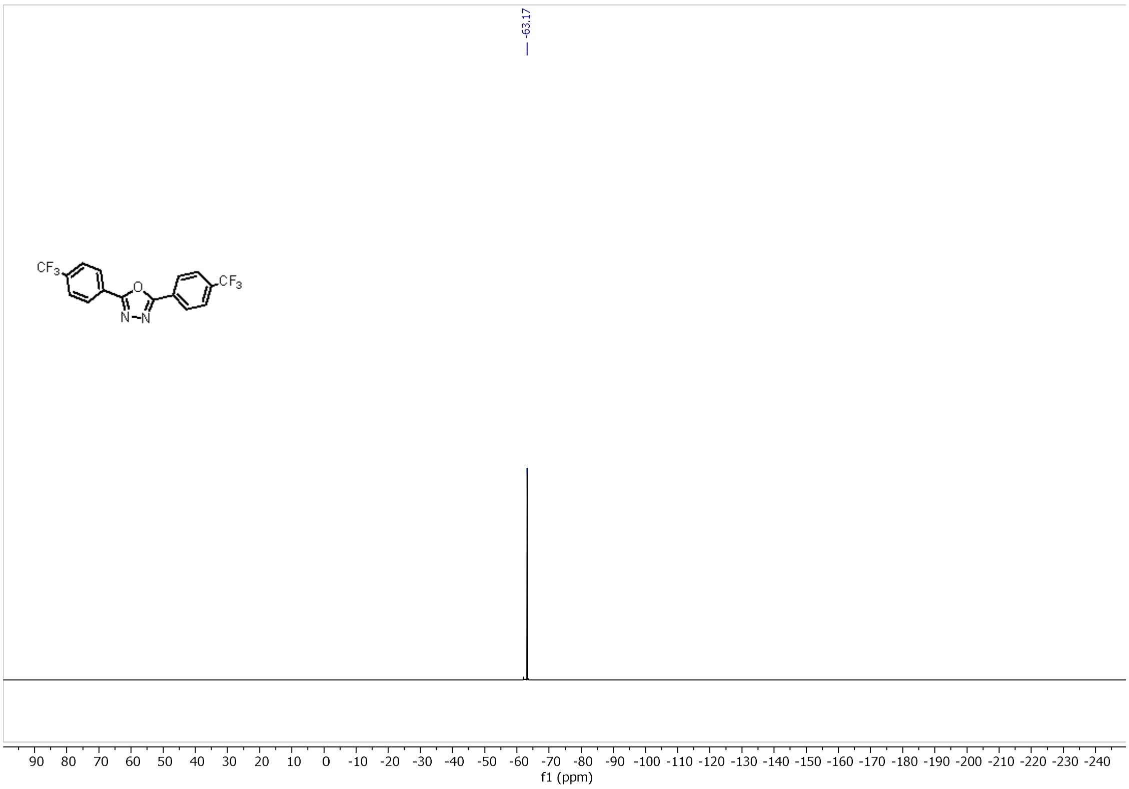

Compound **9** – $^{13}C\{^1H\}$, $CDCl_3$, 101 MHz

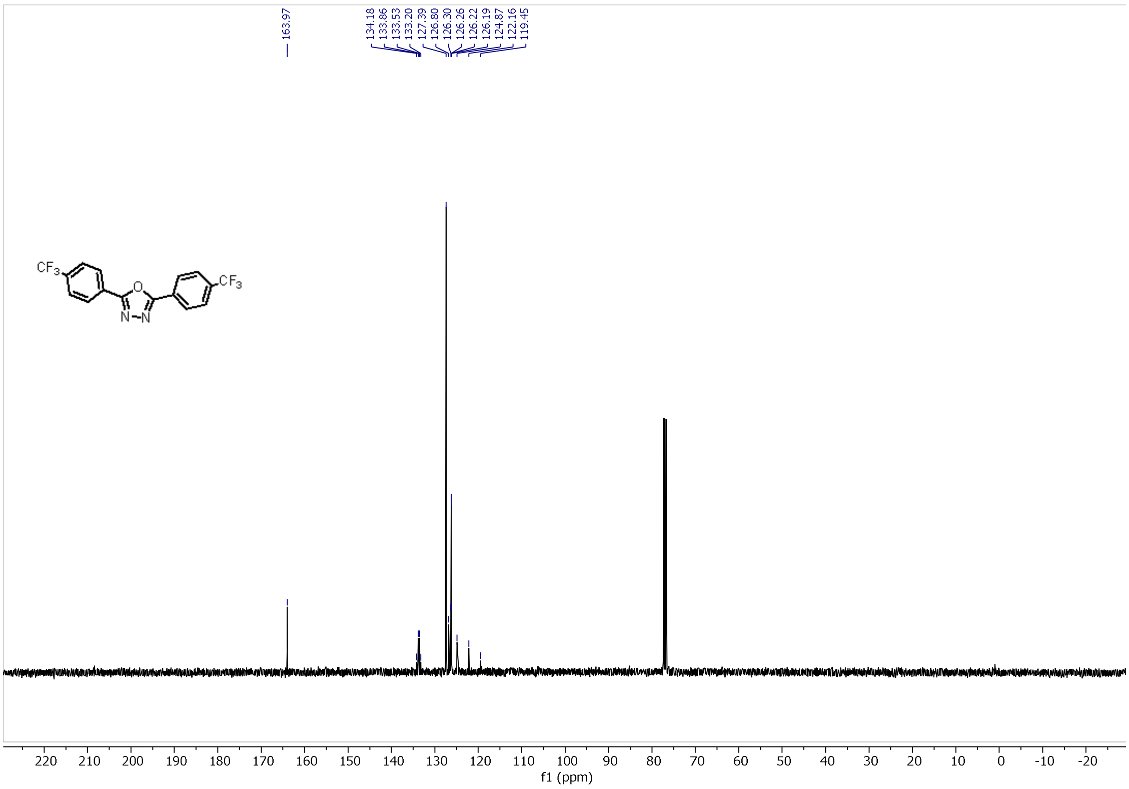

ethyl 4-(5-(4-(N,N-dipropylsulfamoyl)phenyl)-1,3,4-oxadiazol-2-yl)benzoate – 10

Compound **10** – ^{1}H, $CDCl_3$, 400 MHz

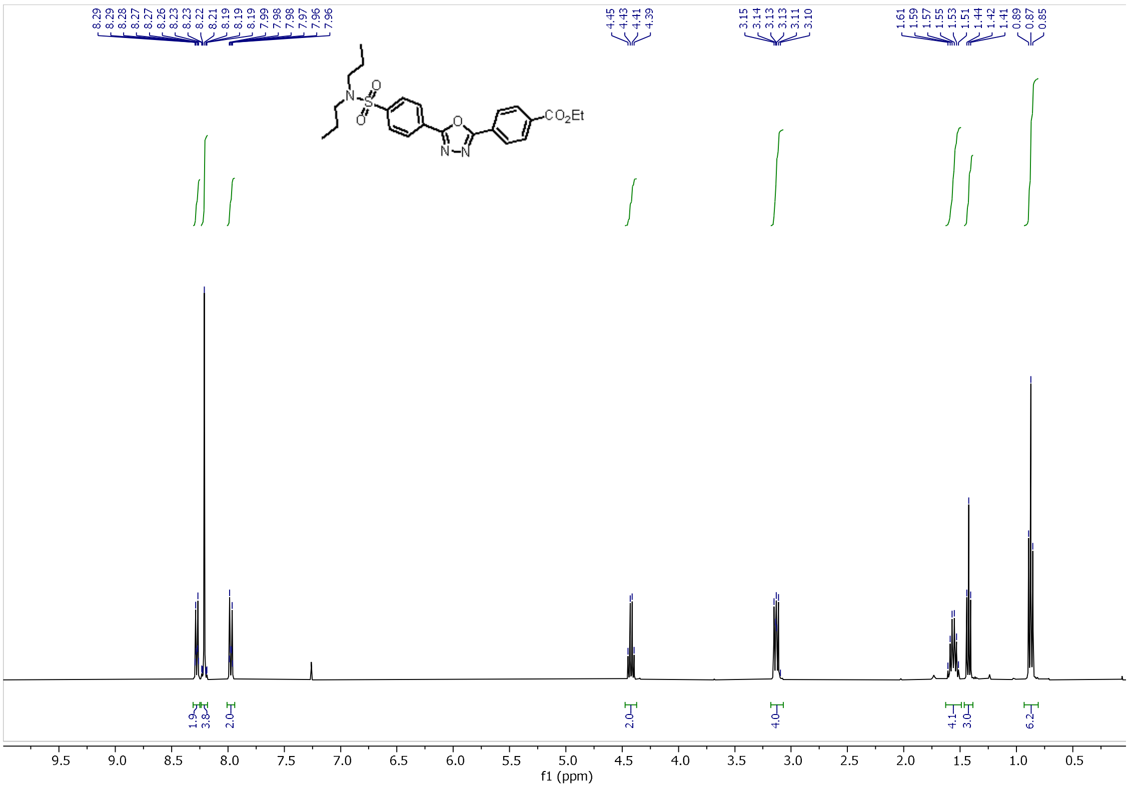

Compound **10** – ^{13}C{^{1}H}, $CDCl_3$, 101 MHz

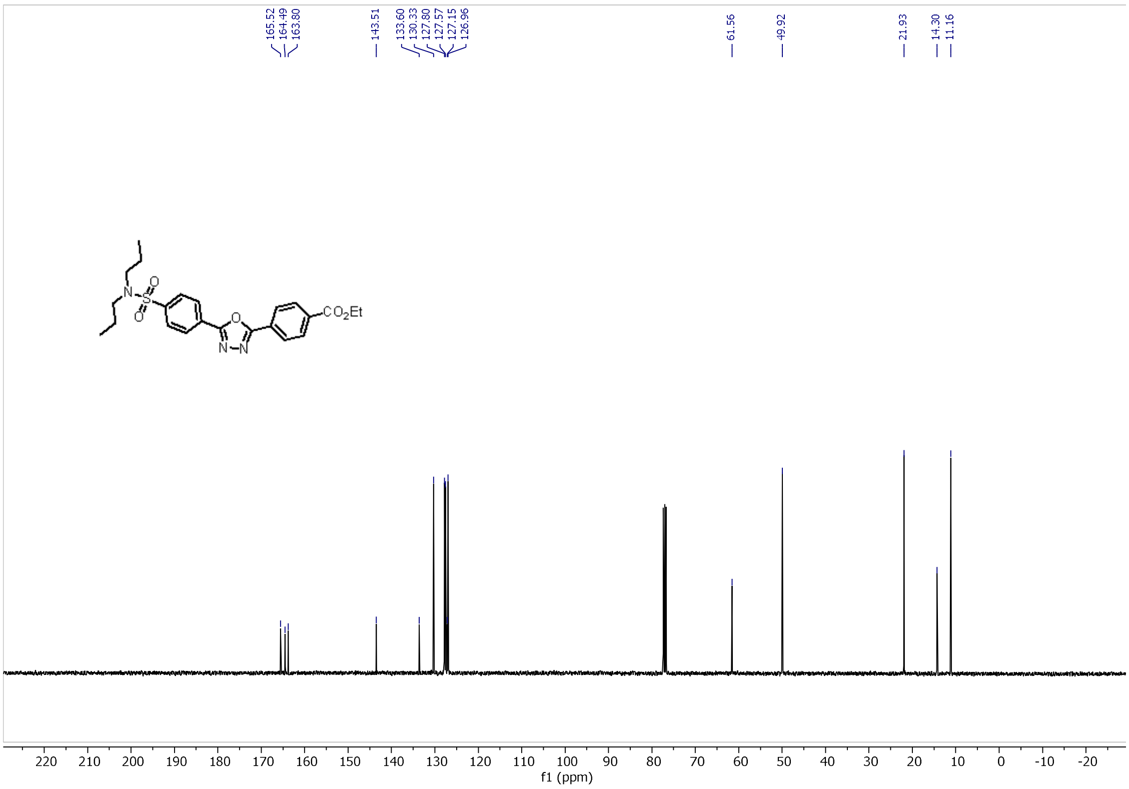

2-(4-bromo-2-methylphenyl)-5-(pyridin-4-yl)-1,3,4-oxadiazole – 11

Compound **11** – ^{1}H, CDCl$_3$, 400 MHz

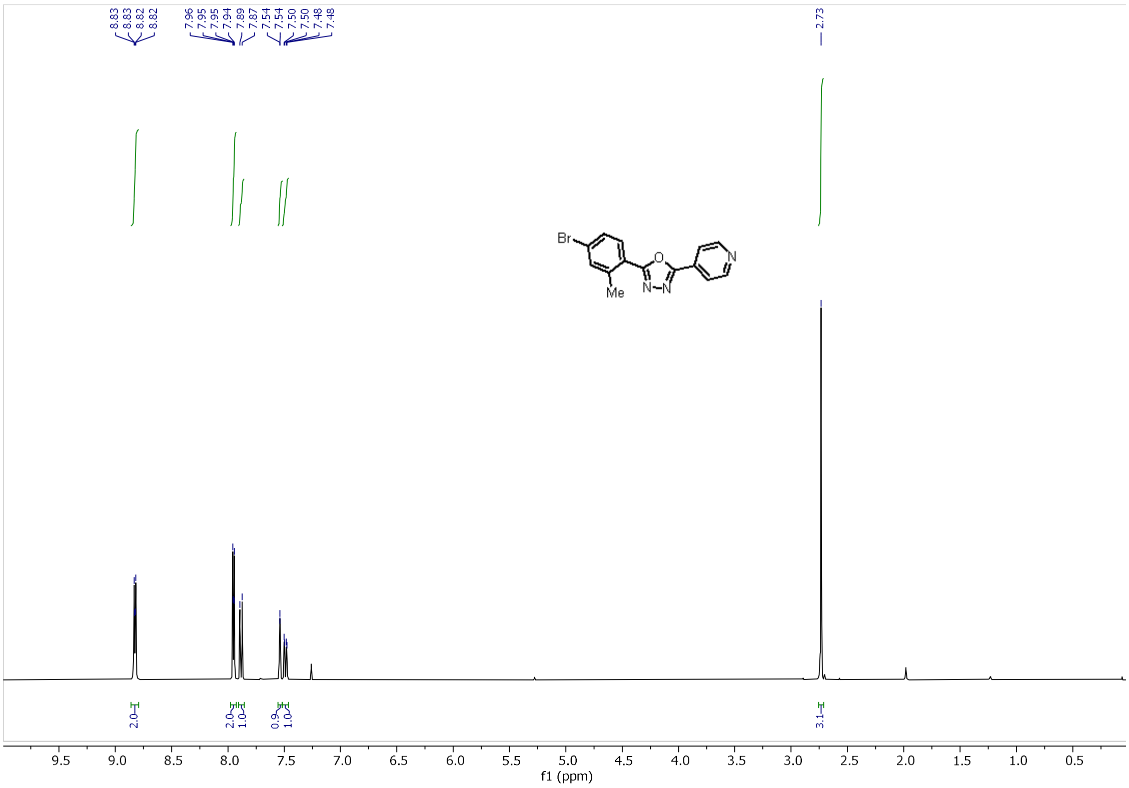

Compound **11** – ^{13}C{^{1}H}, CDCl$_3$, 101 MHz

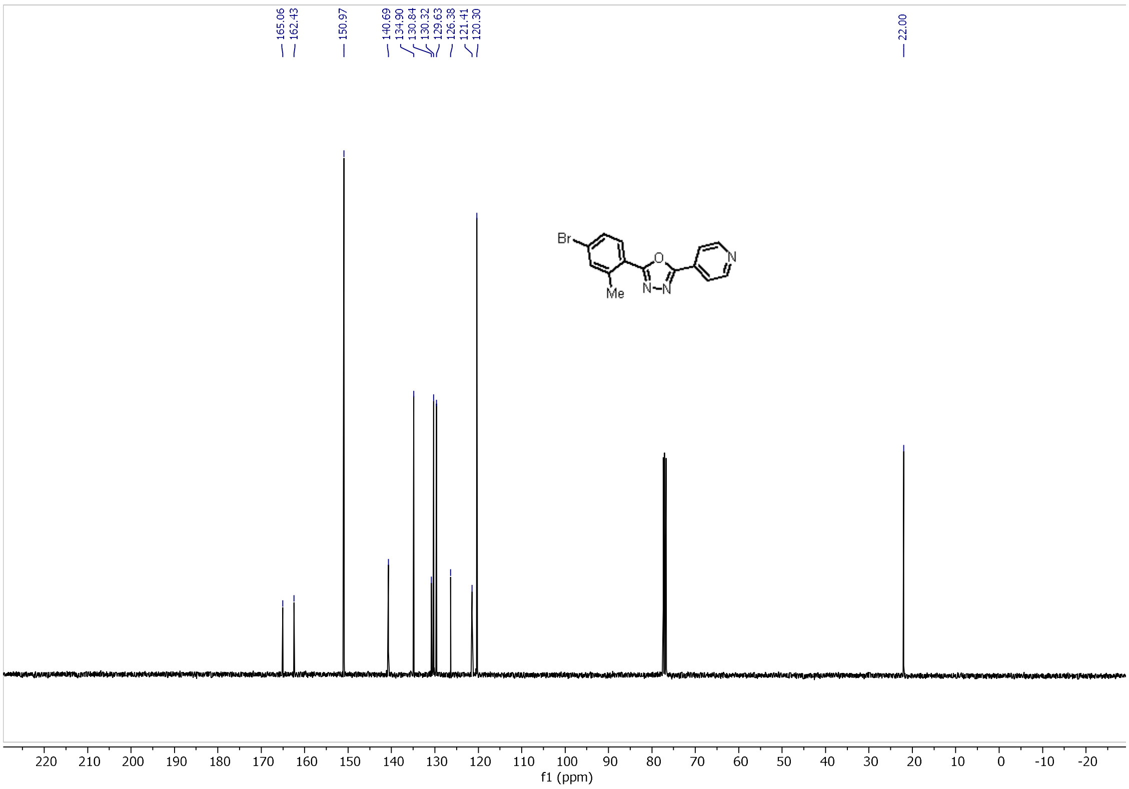

2-(3-fluoro-4-(trifluoromethyl)phenyl)-5-(pyridin-2-yl)-1,3,4-oxadiazole – 12

Compound **12** – ^{1}H, $CDCl_3$, 400 MHz

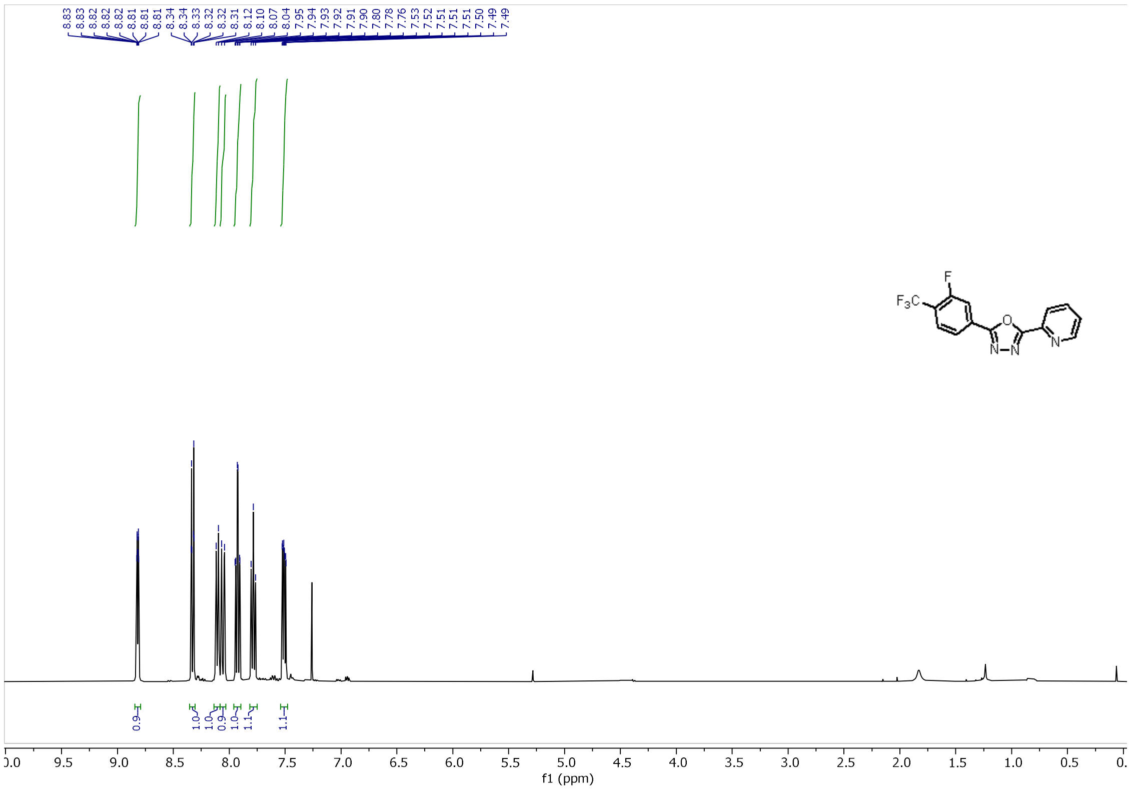

Compound **12** – ^{19}F, $CDCl_3$, 376 MHz

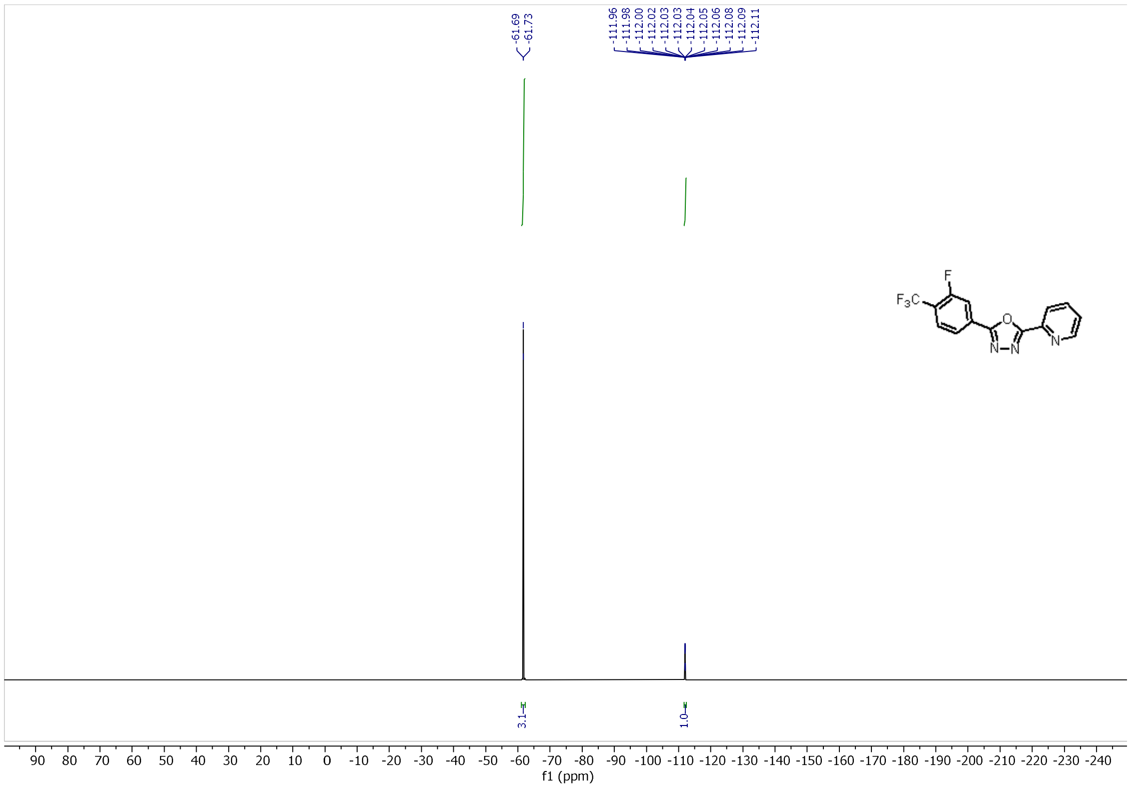

Compound **12** – $^{13}C\{^{1}H\}$, $CDCl_3$, 101 MHz

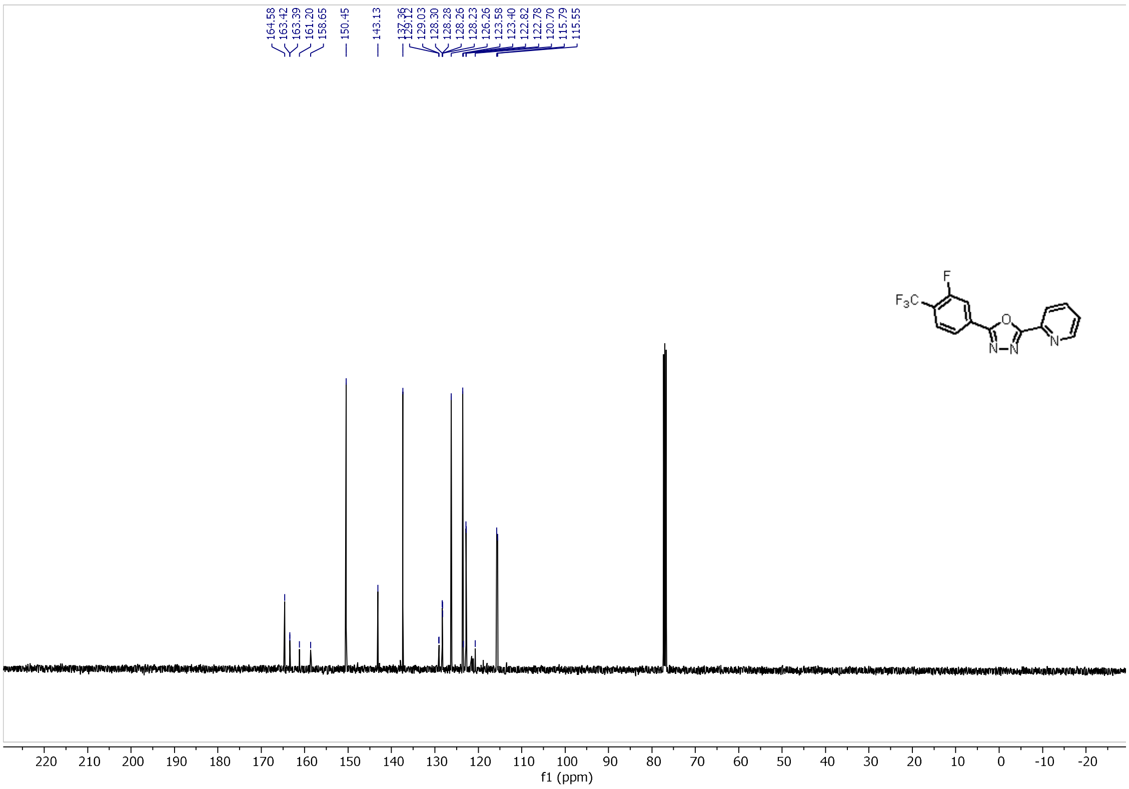

4-(5-(pyridin-3-yl)-1,3,4-oxadiazol-2-yl)benzonitrile – 13

Compound **13** – ^{1}H, $CDCl_3$, 400 MHz

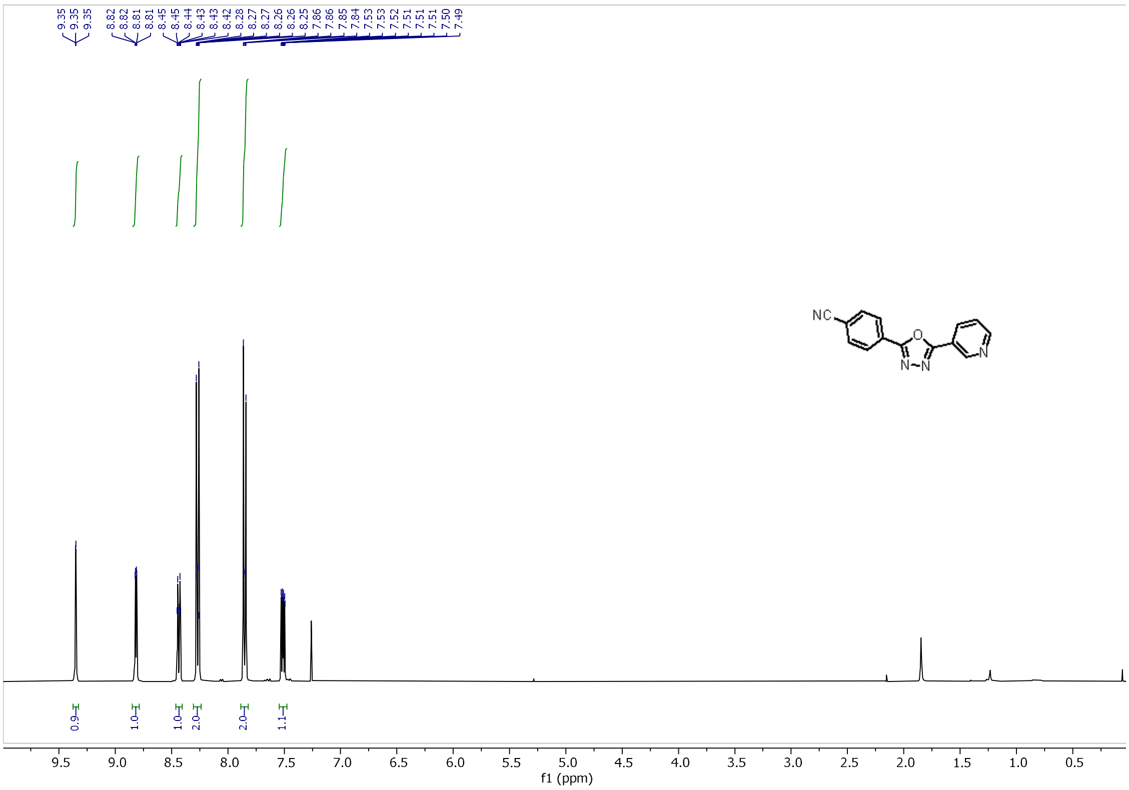

Compound **13** – $^{13}C\{^{1}H\}$, $CDCl_3$, 101 MHz

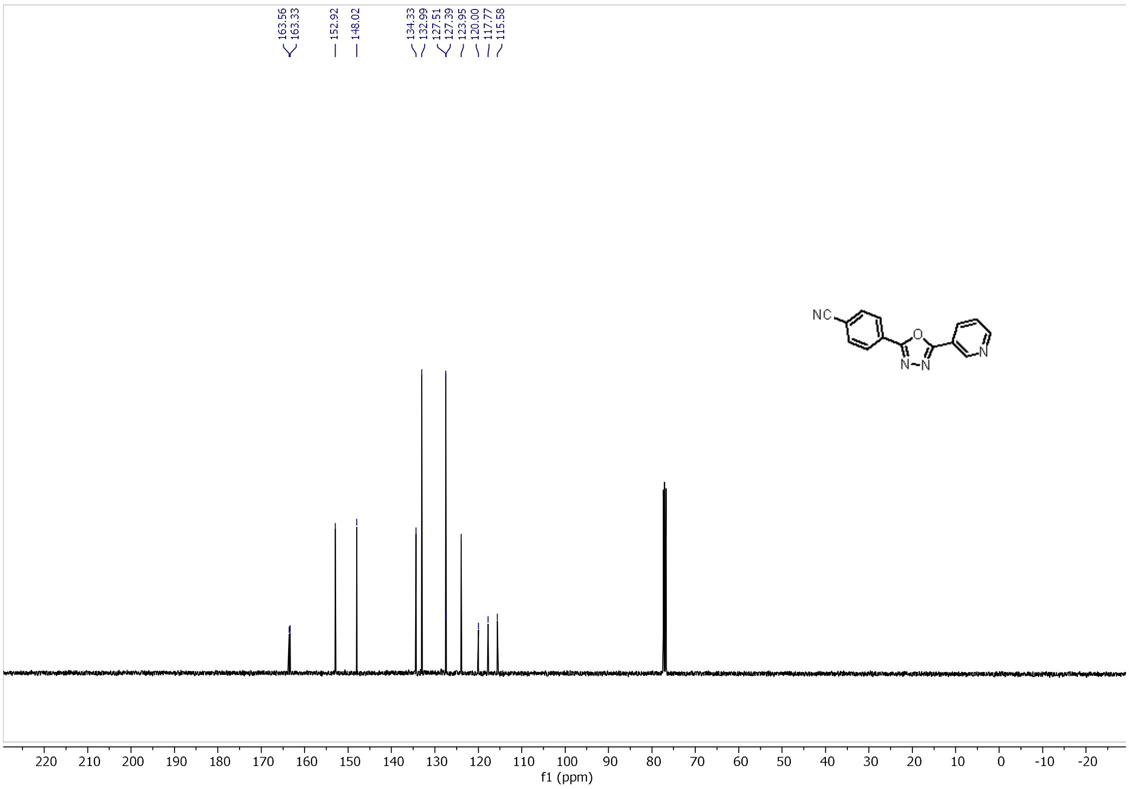

(E)-4-(5-styryl-1,3,4-oxadiazol-2-yl)benzonitrile – 14

Compound **14** – ^{1}H, $CDCl_3$, 400 MHz

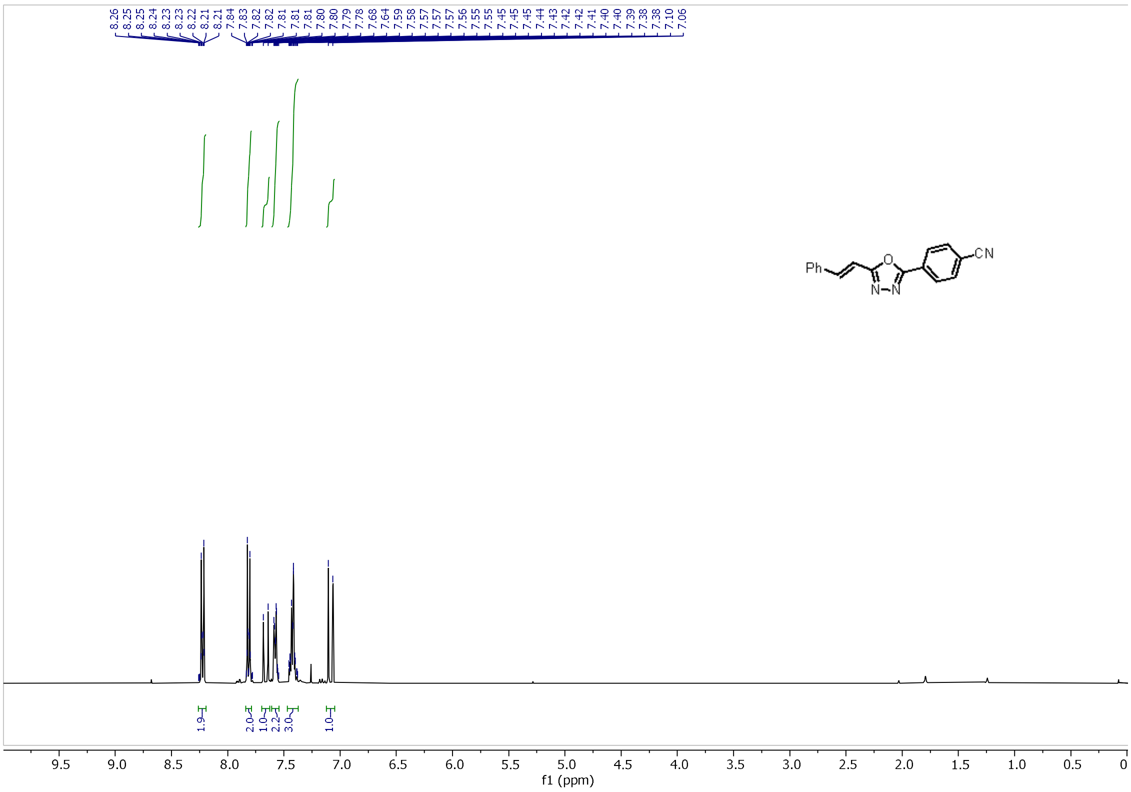

Compound **14** – $^{13}C\{^1H\}$, $CDCl_3$, 101 MHz

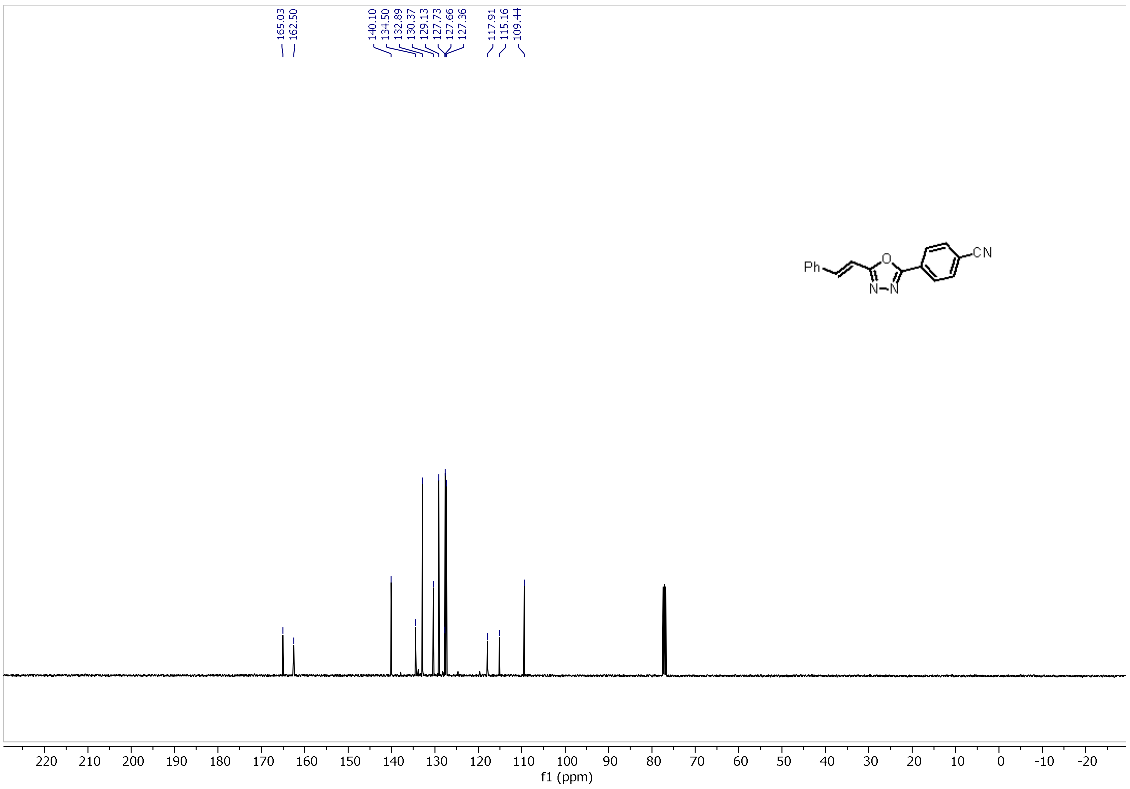

2-(3,5-dimethylphenyl)-5-(1-phenylcyclopropyl)-1,3,4-oxadiazole – 15

Compound **15** – ^1H, $CDCl_3$, 400 MHz

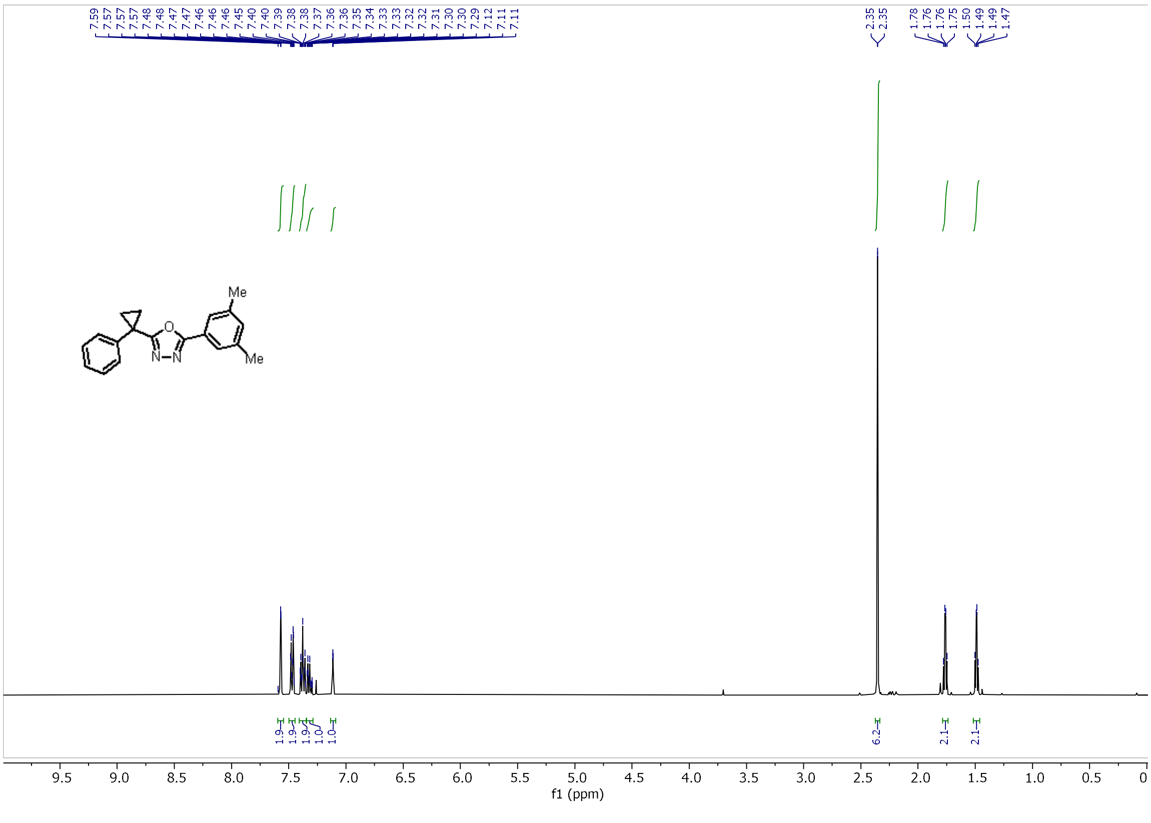

Compound **15** – $^{13}C\{^{1}H\}$, $CDCl_3$, 101 MHz

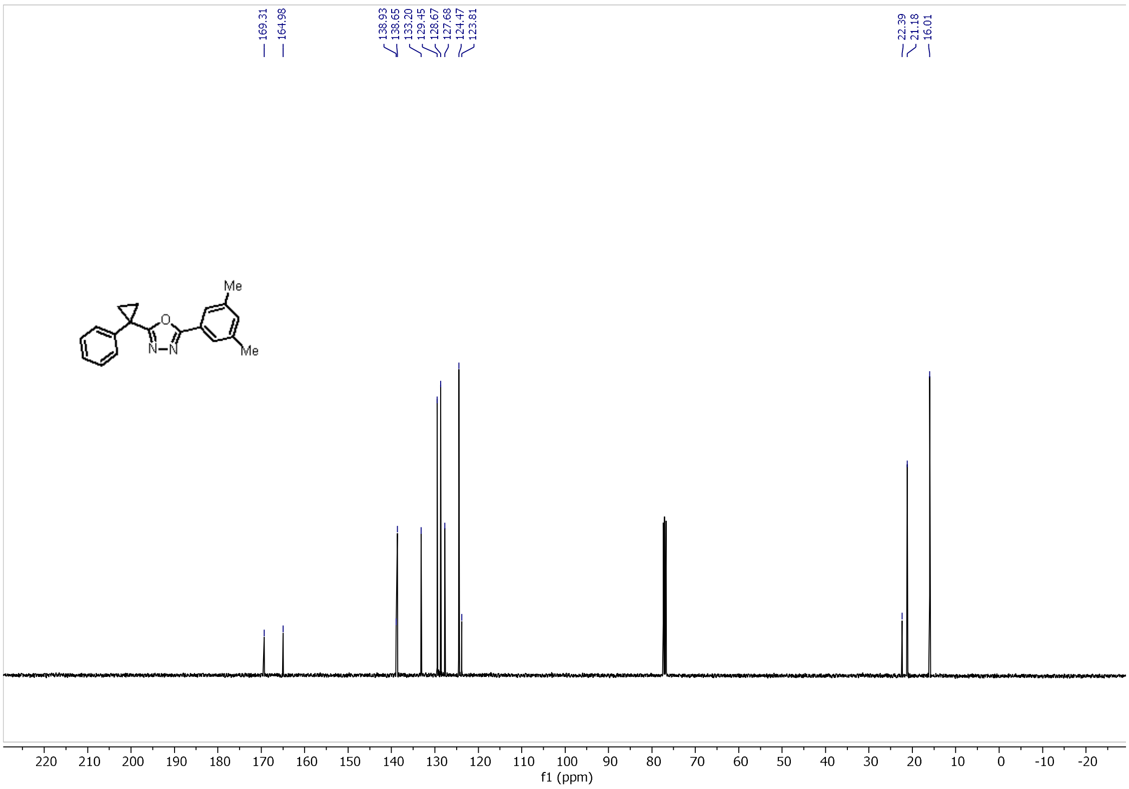

2-(2-methoxyphenyl)-5-(pyridin-3-yl)-1,3,4-oxadiazole – 16

Compound **16** – ^{1}H, $CDCl_3$, 400 MHz

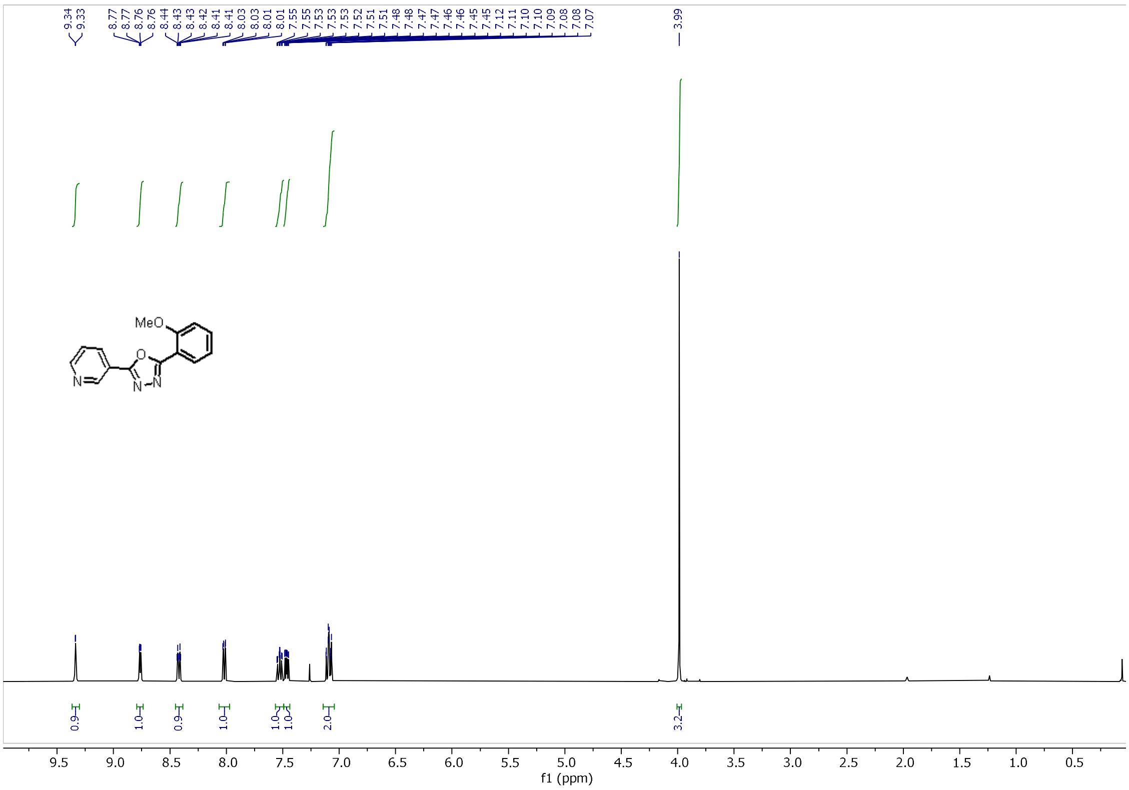

Compound **16** – $^{13}C\{^{1}H\}$, $CDCl_3$, 101 MHz

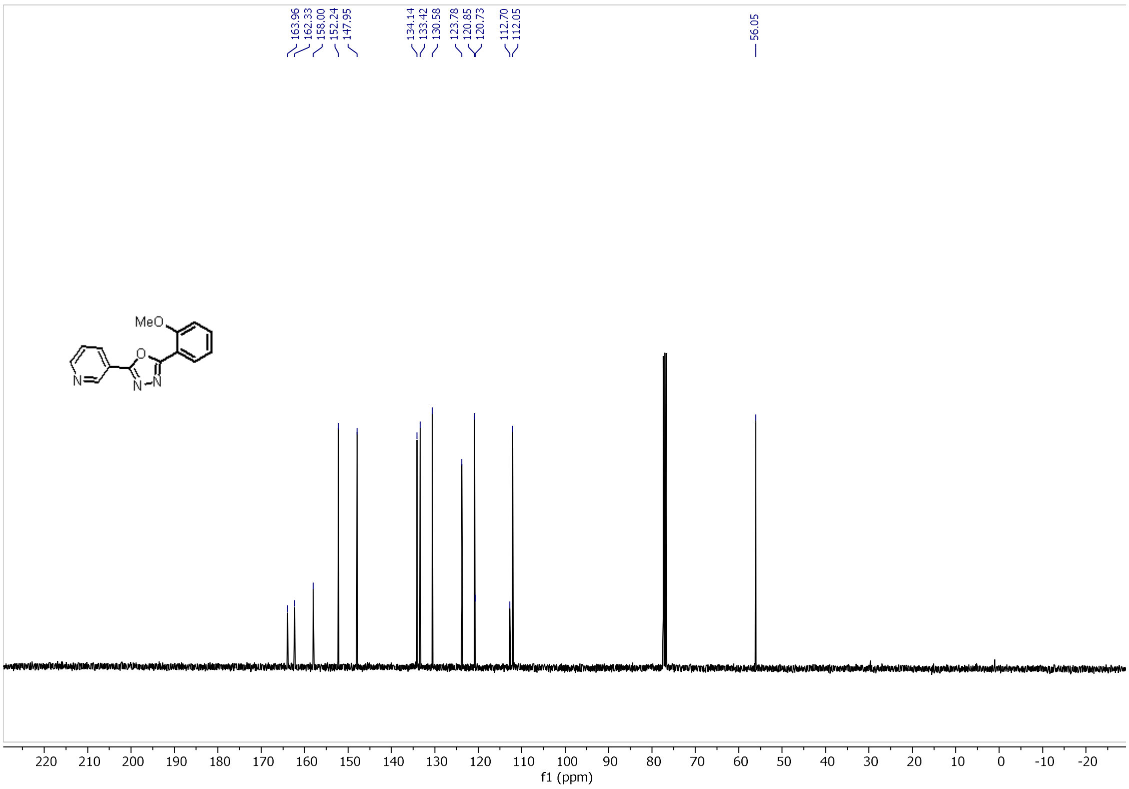

2-(5-(2,5-dimethylphenoxy)-2-methylpentan-2-yl)-5-(naphthalen-1-yl)-1,3,4-oxadiazole – 17

Compound **17** – ^{1}H, $CDCl_3$, 400 MHz

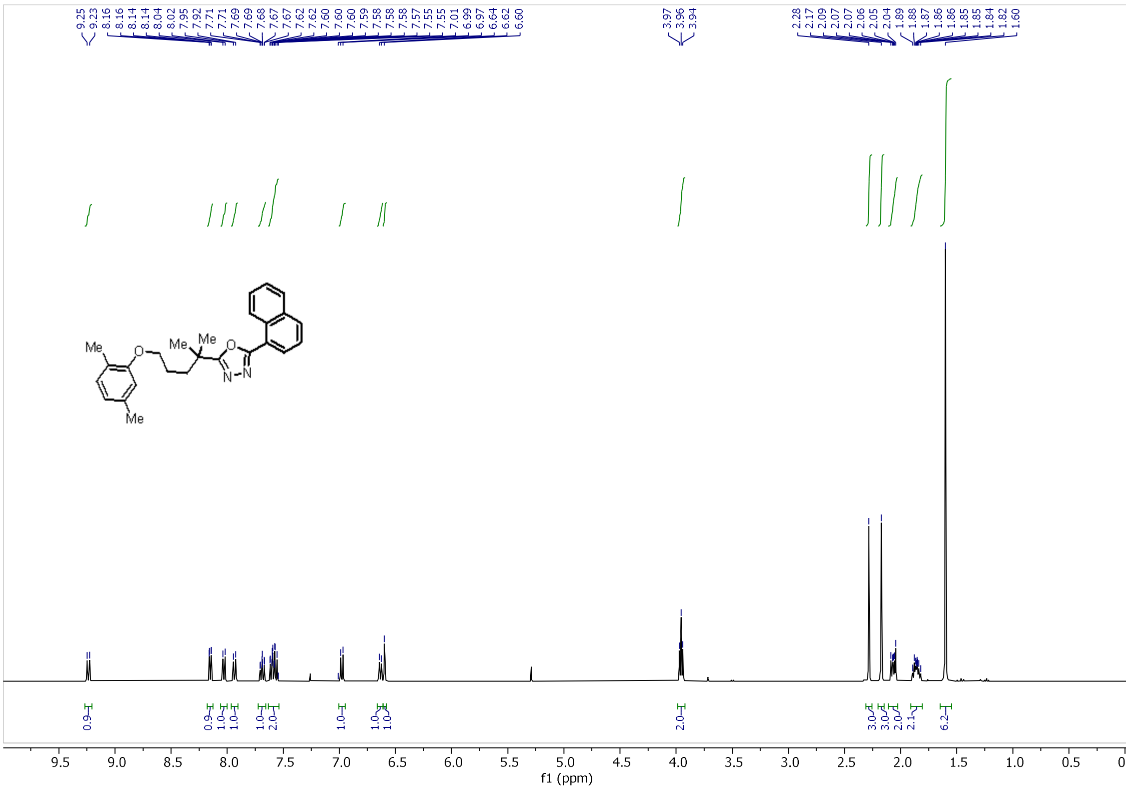

Compound **17** – $^{13}C\{^{1}H\}$, $CDCl_3$, 101 MHz

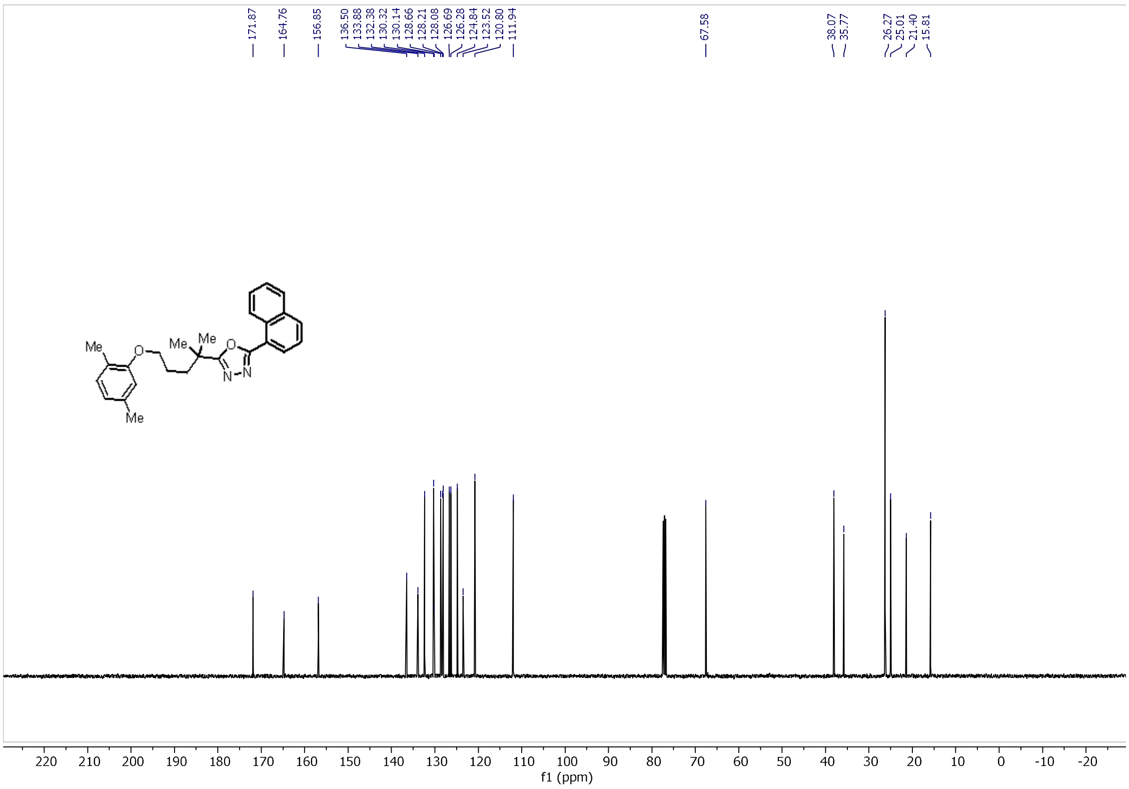

2-(1-(6-methoxynaphthalen-2-yl)ethyl)-5-(naphthalen-2-yl)-1,3,4-oxadiazole – 18

Compound **18** – ^{1}H, $CDCl_3$, 400 MHz

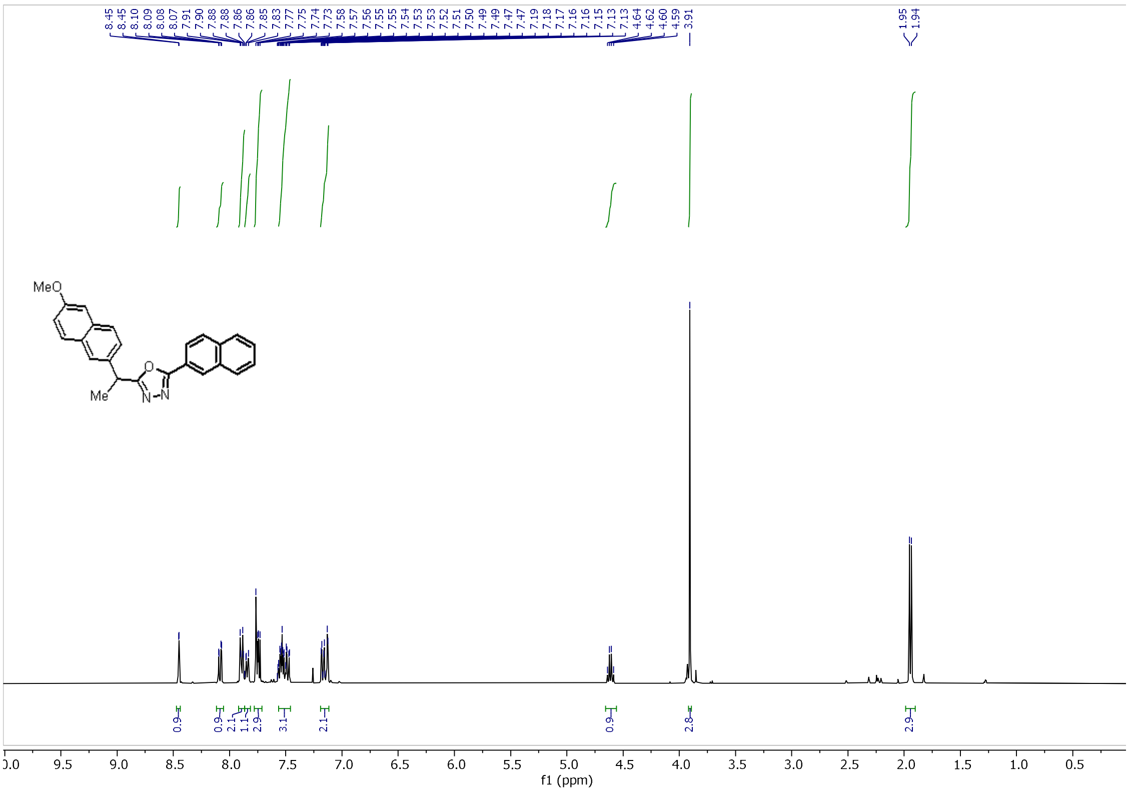

Compound **18** – $^{13}C\{^{1}H\}$, $CDCl_3$, 101 MHz

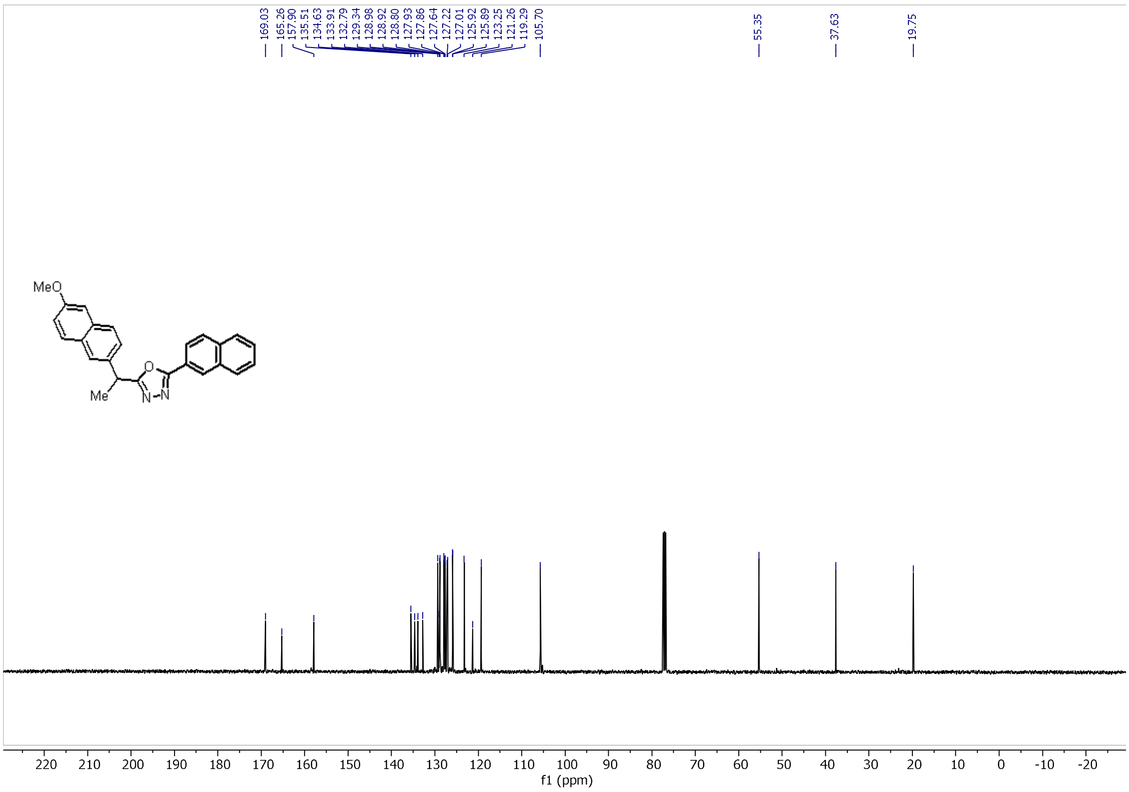

2-(1-(4-isobutylphenyl)ethyl)-5-(3-methoxyphenyl)-1,3,4-oxadiazole – 19

Compound **19** – ^{1}H, $CDCl_3$, 400 MHz

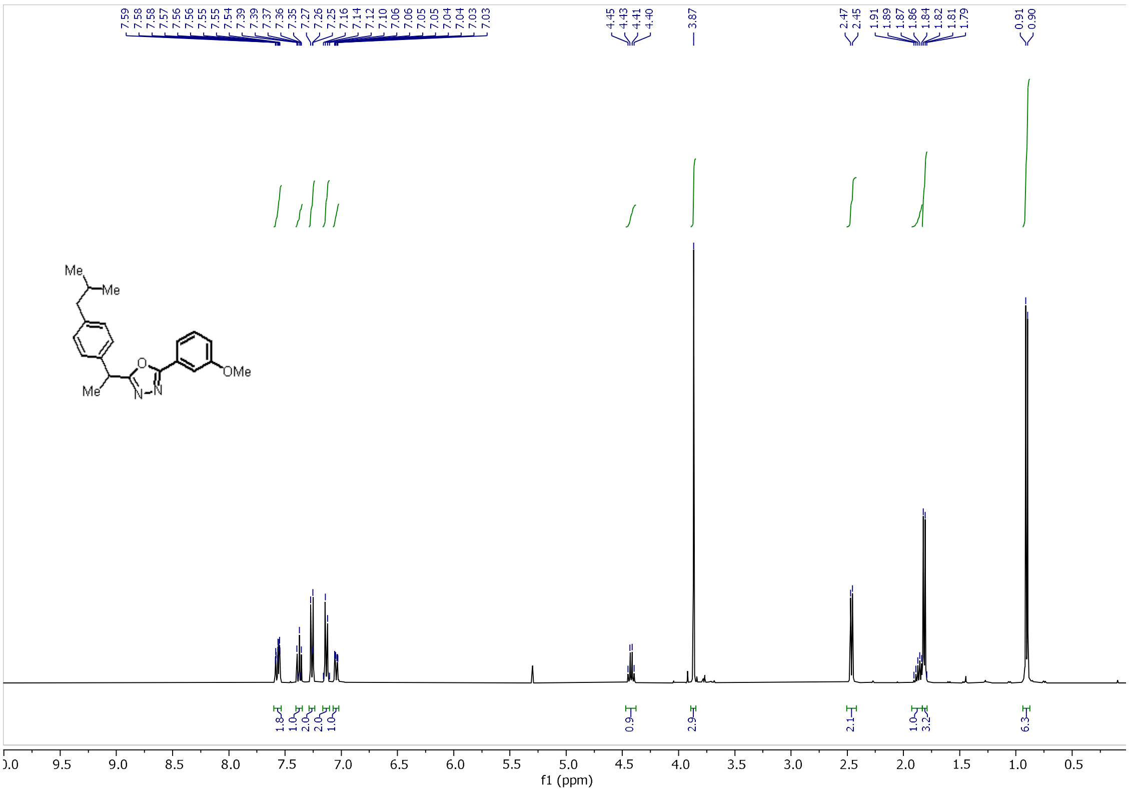

Compound **19** – $^{13}C\{^{1}H\}$, $CDCl_3$, 101 MHz

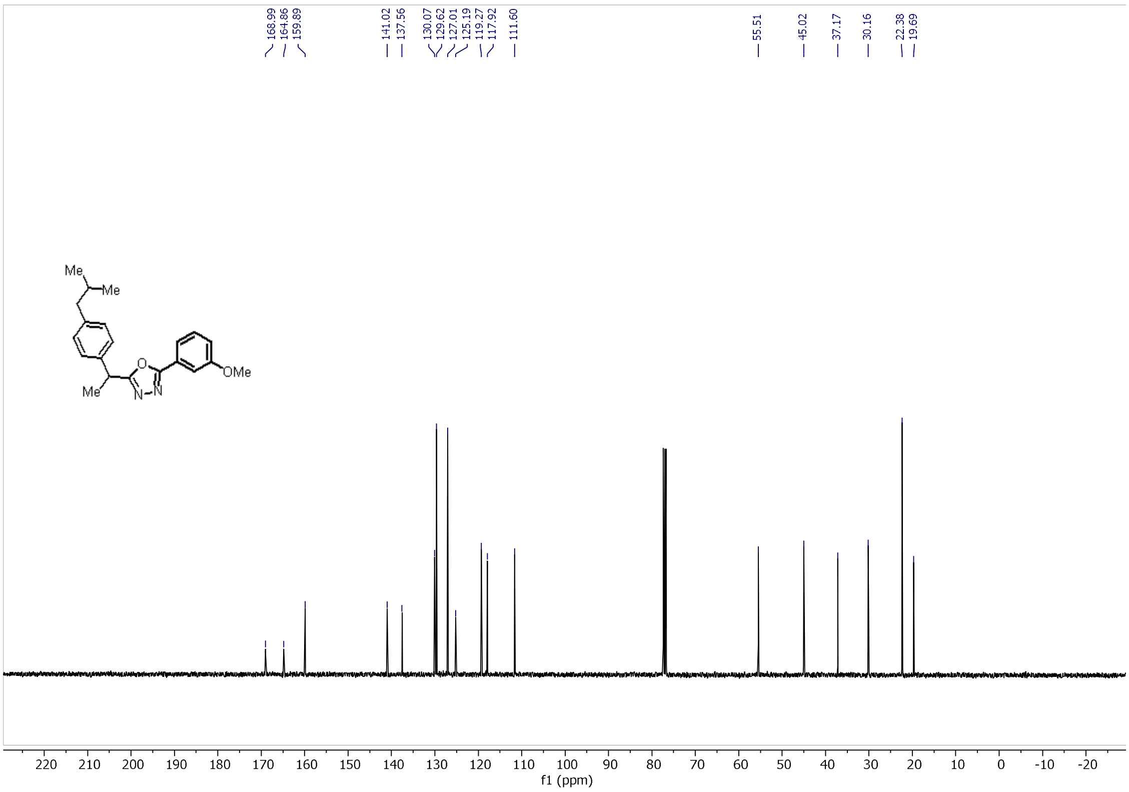

2-(4-fluorophenyl)-5-(piperidin-1-yl)-1,3,4-oxadiazole – 20

Compound **20** – ^{1}H, $CDCl_3$, 400 MHz

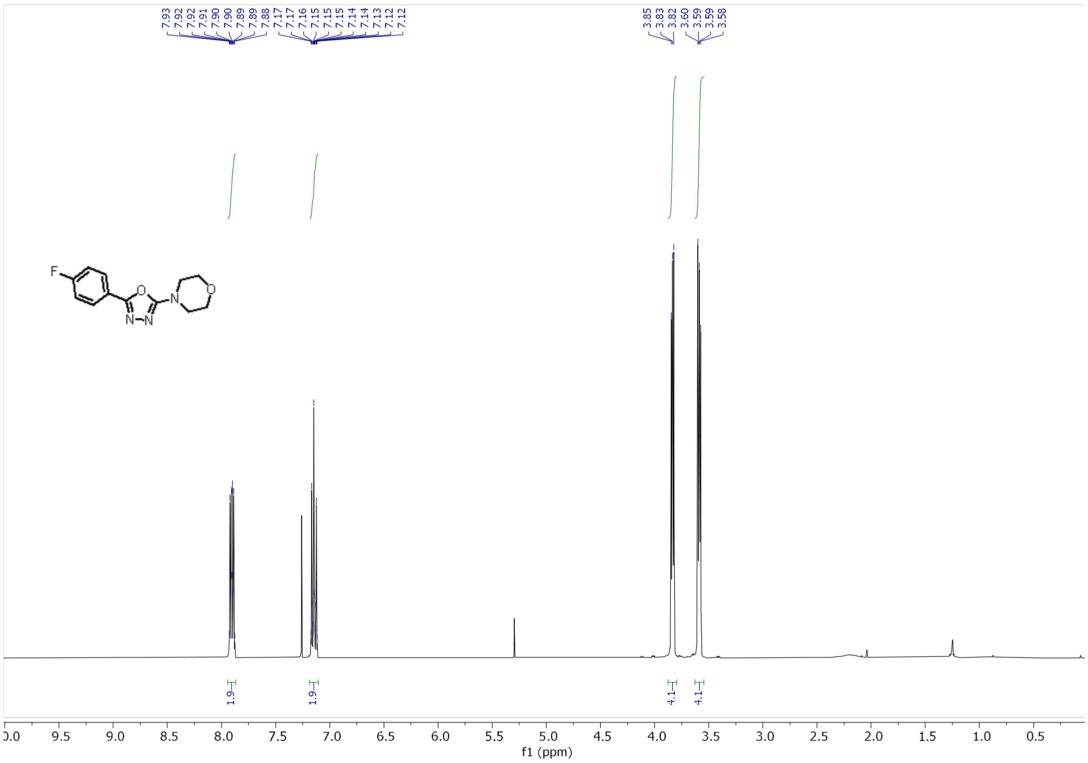

Compound **20** – ^{19}F, $CDCl_3$, 376 MHz

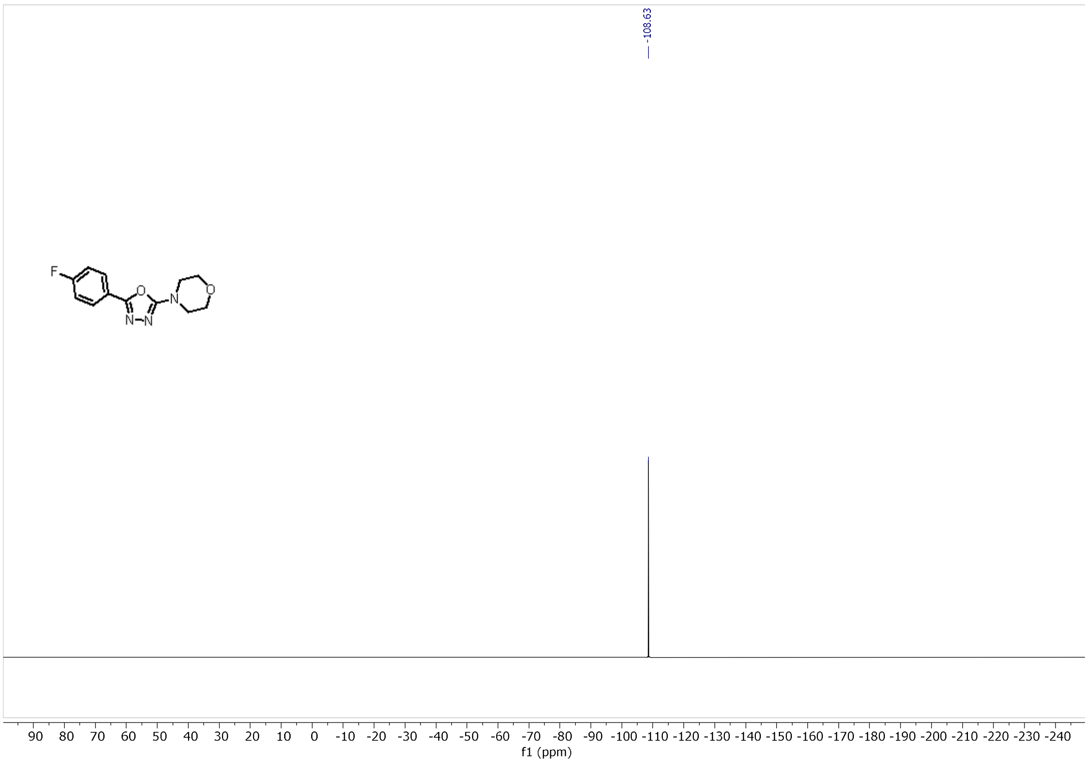

Compound **20** – $^{13}C\{^1H\}$, $CDCl_3$, 101 MHz

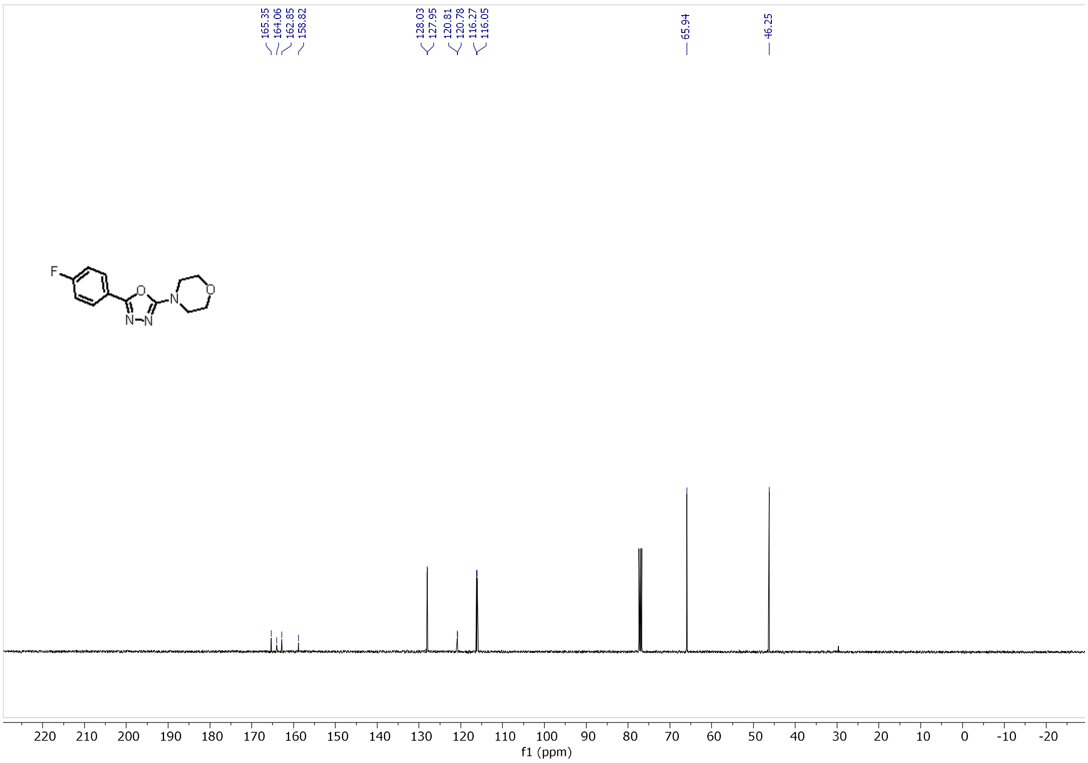

4-(5-morpholino-1,3,4-oxadiazol-2-yl)-N,N-dipropylbenzenesulfonamide – 21

Compound **21** – ^{1}H, $CDCl_3$, 400 MHz

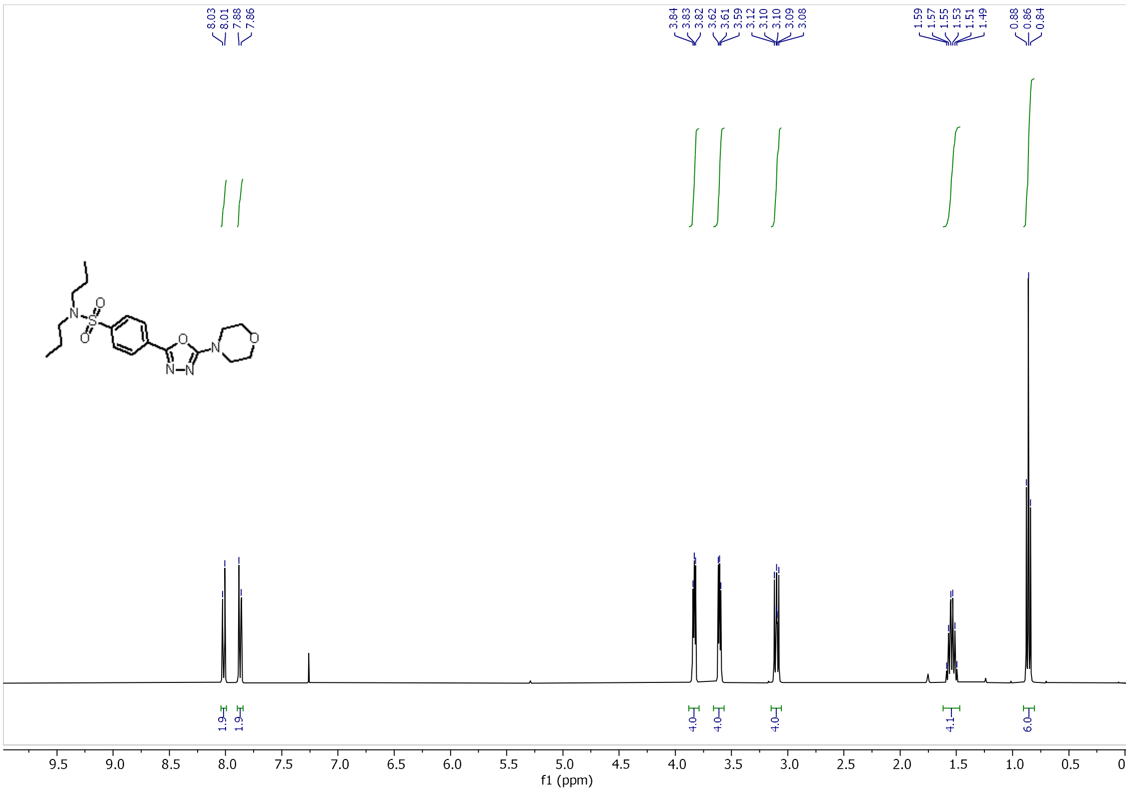

Compound **21** – $^{13}C\{^{1}H\}$, $CDCl_3$, 101 MHz

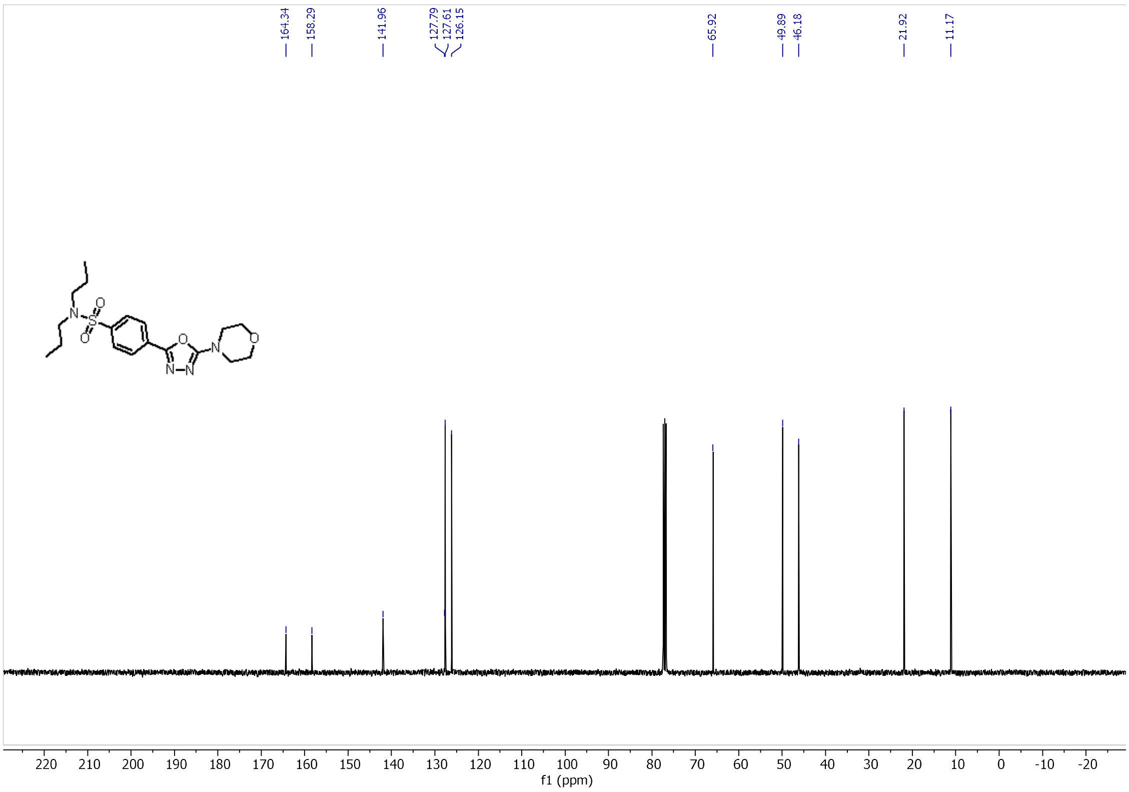

4-(5-(1-(6-methoxynaphthalen-2-yl)ethyl)-1,3,4-oxadiazol-2-yl)morpholine – 22

Compound **22** – ^{1}H, CDCl$_3$, 400 MHz

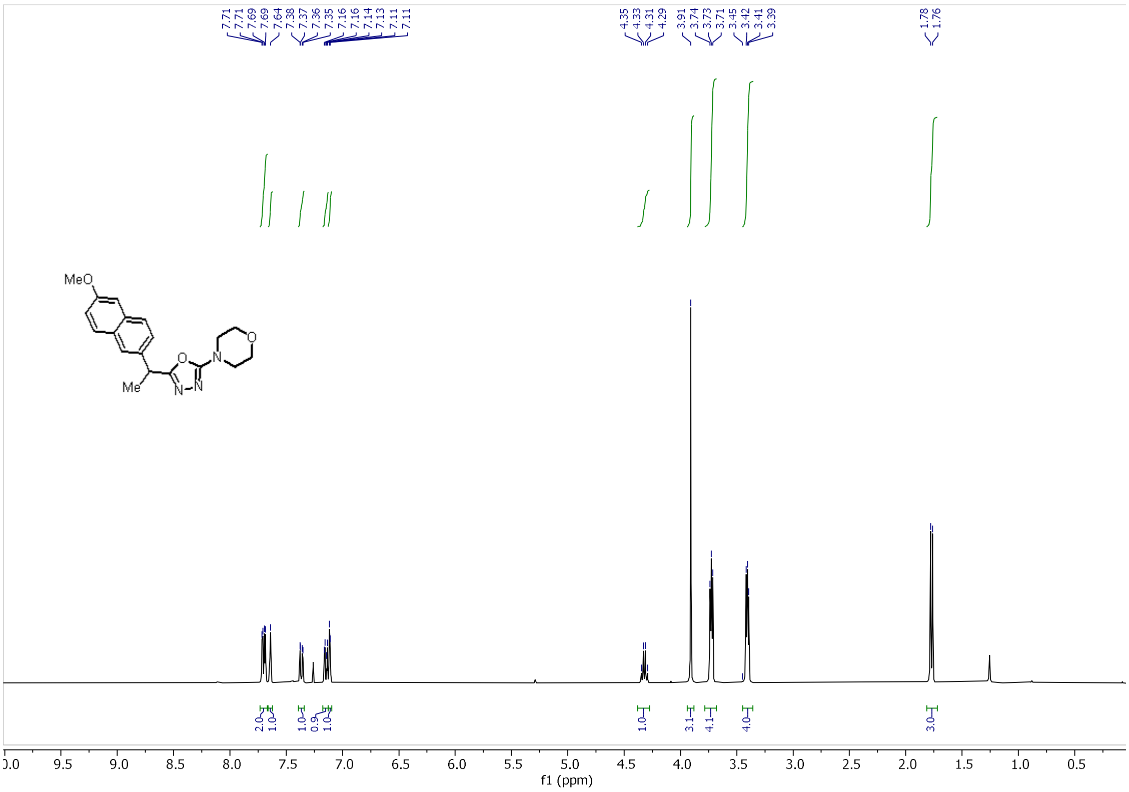

Compound **22** – ^{13}C{^{1}H}, CDCl$_3$, 101 MHz

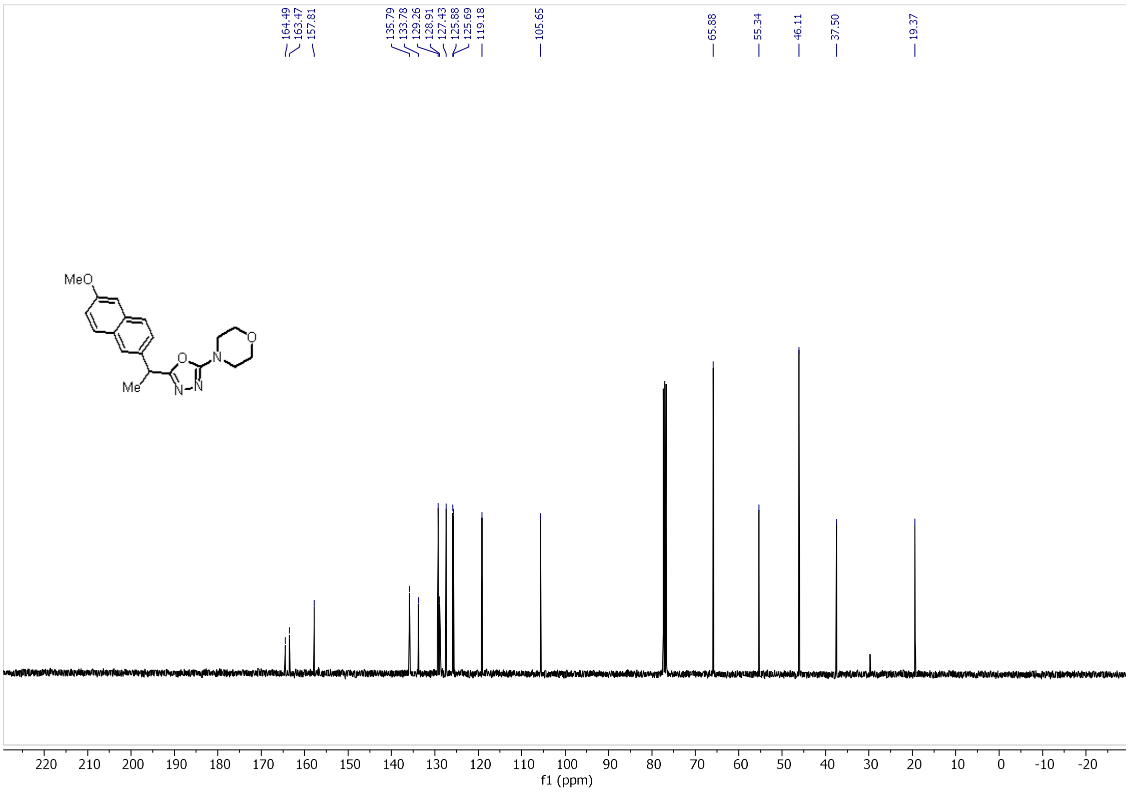

4-(5-(1-(4-isobutylphenyl)ethyl)-1,3,4-oxadiazol-2-yl)morpholine – 23

Compound **23** – ^{1}H, $CDCl_3$, 400 MHz

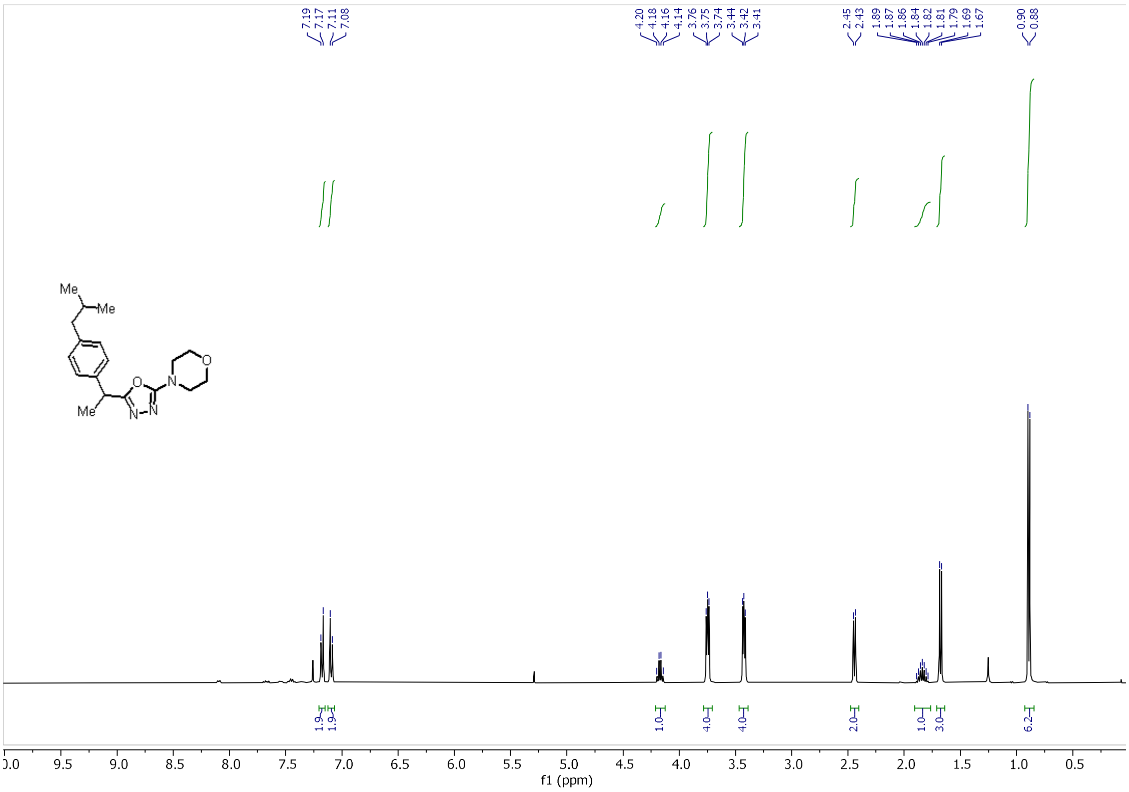

Compound **23** – $^{13}C\{^{1}H\}$, $CDCl_3$, 101 MHz

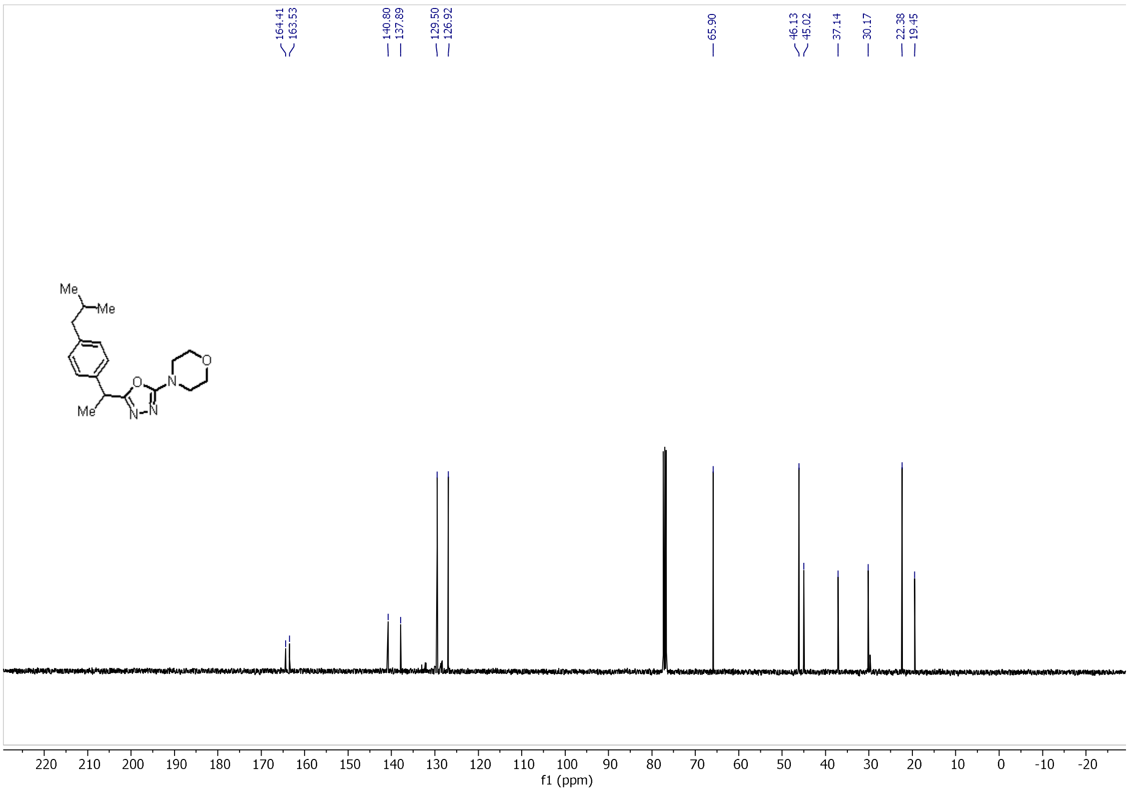

4-(5-(5-(2,5-dimethylphenoxy)-2-methylpentan-2-yl)-1,3,4-oxadiazol-2-yl)morpholine – 24

Compound **24** – ^{1}H, $CDCl_3$, 400 MHz

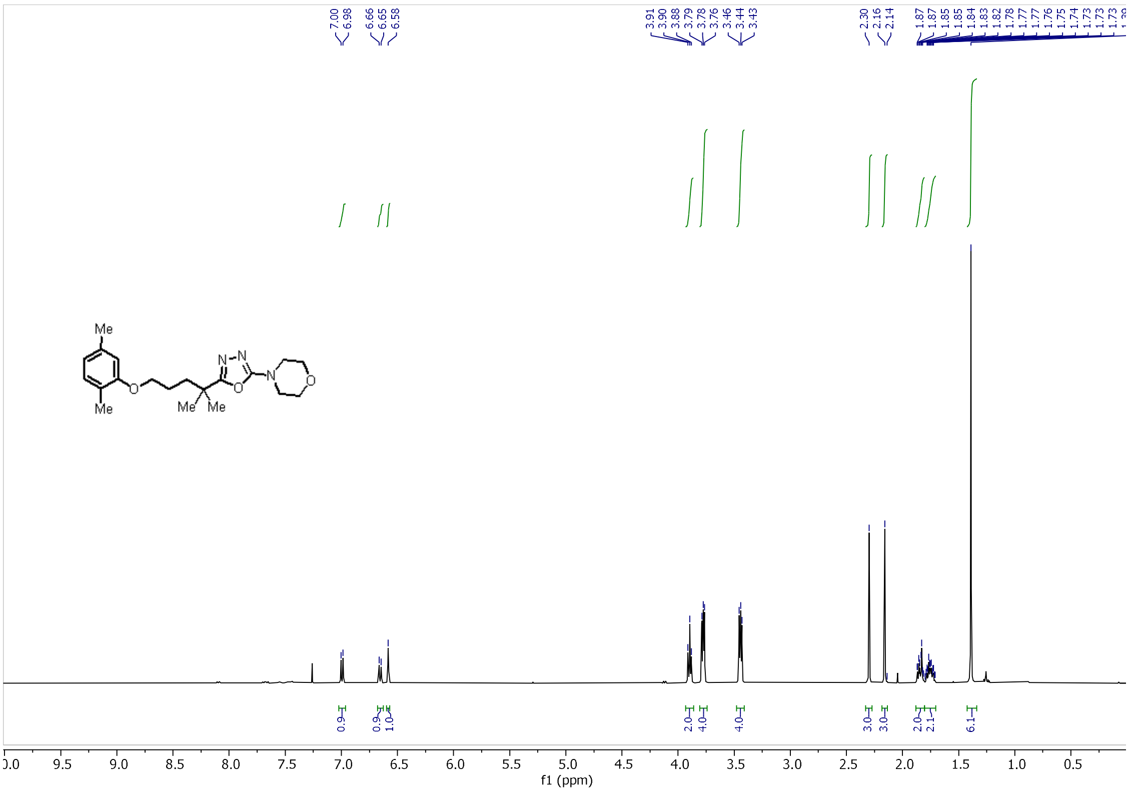

Compound **24** – $^{13}C\{^{1}H\}$, $CDCl_3$, 101 MHz

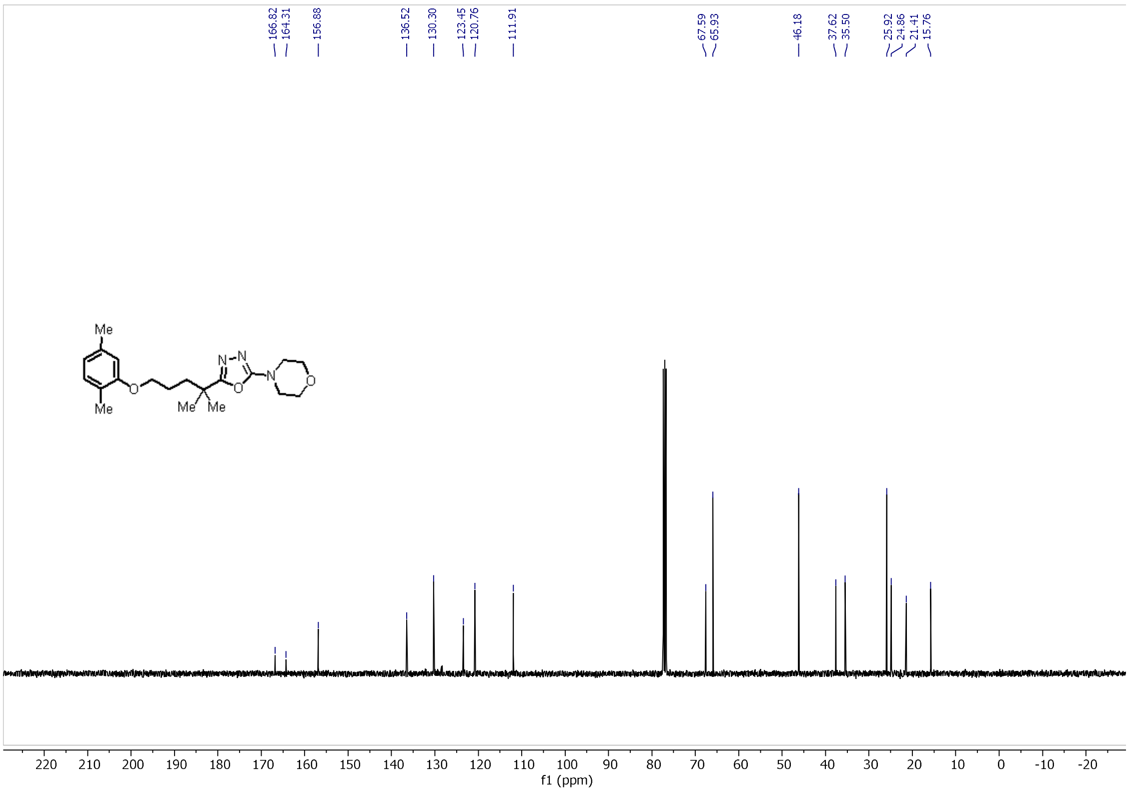

N-(3-(10,11-dihydro-5H-dibenzo[a,d][7]annulen-5-ylidene)propyl)-5-(5-(2,5-dimethylphenoxy)-2-methylpentan-2-yl)-N-methyl-1,3,4-oxadiazol-2-amine – 25

Compound **25** – ^{1}H, $CDCl_3$, 400 MHz

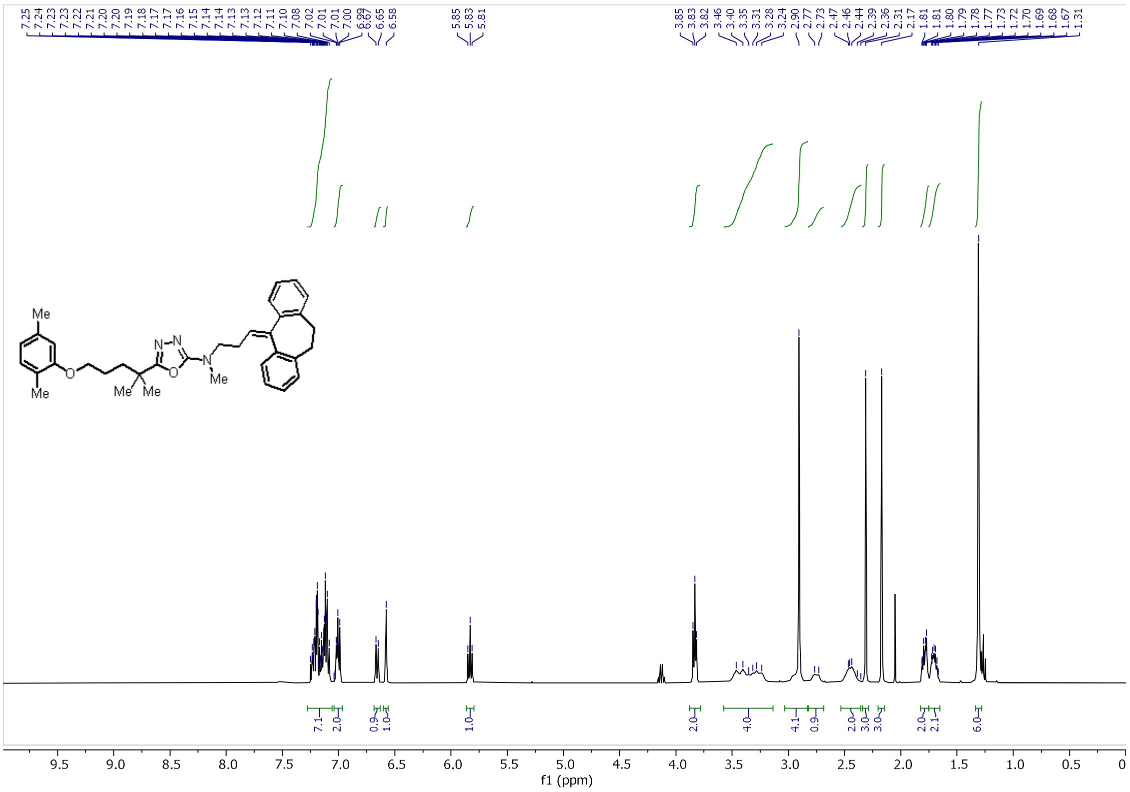

Compound **25** – $^{13}C\{^{1}H\}$, $CDCl_3$, 151 MHz

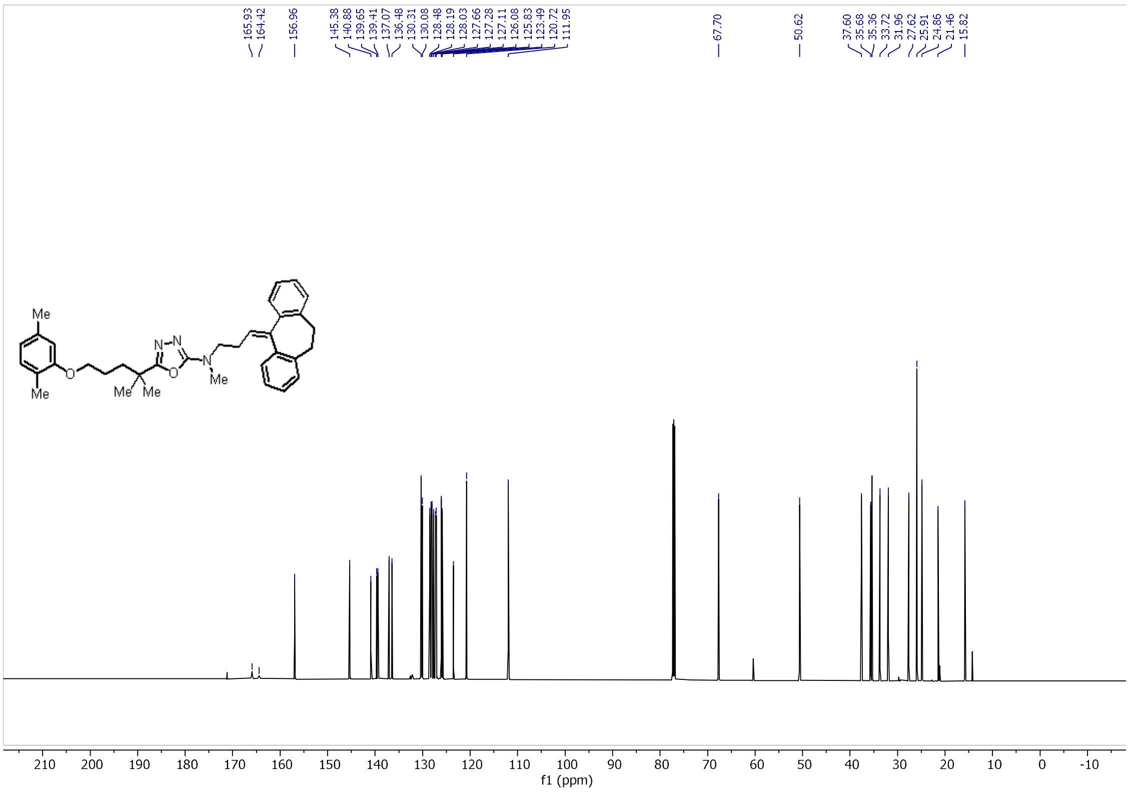

References

(1) M. M. Bio, G. Javadi and Z. J. Song, An Improved Synthesis of N-Isocyanoiminotriphenylphosphorane and Its Use in the Preparation of Diazoketones. *Synthesis.,* 2005, 19-2

(2) Q. Gou, G, Liu, Z.-N. Liu and J. Qin, Pd^{II}-Catalyzed Intermolecular Amination of Unactivated $C(sp^3)$-H Bonds. *Chem. - Eur. J.,* 2015, *21*, 15491-15495.

(3) S. Graßl, Y.-H. Chen, C. Hamze, C. P. Tüllmann and P. Knochel, Late Stage Functionalization of Secondary Amines via a Cobalt-Catalyzed Electrophilic Amination of Organozinc Reagents. *Org. Lett.,* 2019, *21*, 494-497.

(4) T. Kawano, T. Yoshizumi, K. Hirano, T. Satoh and M. Miura, Copper-Mediated Direct Arylation of 1,3,4-Oxadiazoles and 1,2,4-Triazoles with Aryl Iodides. *Org. Lett.,* 2009, *11*, 3072-3075.
